# Global, regional, and national burden of Alzheimer's disease and other dementias, 1990–2019

**DOI:** 10.3389/fnagi.2022.937486

**Published:** 2022-10-10

**Authors:** Xue Li, Xiaojin Feng, Xiaodong Sun, Ningning Hou, Fang Han, Yongping Liu

**Affiliations:** ^1^Department of Endocrinology and Metabolism, Affiliated Hospital of Weifang Medical University, Weifang, China; ^2^Department of Pathology, Affiliated Hospital of Weifang Medical University, Weifang, China; ^3^Department of Clinical Research Center, Affiliated Hospital of Weifang Medical University, Weifang, China

**Keywords:** Alzheimer's disease and other dementias, Global Burden of Disease, estimated annual percentage change, risk factors, age-standardized rate (ASR)

## Abstract

**Background:**

With the increase in the aging population worldwide, Alzheimer's disease has become a rapidly increasing public health concern. Monitoring the dementia disease burden will support health development strategies by providing scientific data.

**Methods:**

Based on the data obtained from the 2019 Global Burden of Disease (GBD) database, the numbers and age-standardized rates (ASRs) of incidence, prevalence, death, and disability-adjusted life-years (DALYs) of Alzheimer's disease and other dementias from 1990 to 2019 were analyzed. Calculated estimated annual percentage changes (EAPCs) and Joinpoint regression analyses were performed to evaluate the trends during this period. We also evaluated the correlations between the epidemiology and the sociodemographic index (SDI), an indicator to evaluate the level of social development in a country or region considering the education rate, economic situation, and total fertility rate.

**Results:**

From 1990 to 2019, the incidence and prevalence of Alzheimer's disease and other dementias increased by 147.95 and 160.84%, respectively. The ASR of incidence, prevalence, death, and DALYs in both men and women consistently increased over the study period. All the ASRs in women were consistently higher than those in men, but the increases were more pronounced in men. In addition, the ASRs of incidence, prevalence, and DALYs were positively correlated with the SDI. Moreover, the proportion of patients over 70 years old with dementia was also positively correlated with the SDI level. Smoking was a major risk factor for the disease burden of dementia in men, while obesity was the major risk factor for women.

**Conclusion:**

From 1990 to 2019, the Alzheimer's disease burden increased worldwide. This trend was more serious in high-SDI areas, especially among elderly populations in high-SDI areas, who should receive additional attention. Policy-makers should take steps to reverse this situation. Notably, women were at a higher risk for the disease, but the risk in men showed a faster increase. We should give attention to the aging population, attach importance to interventions targeting dementia risk factors, and formulate action plans to address the increasing incidence of dementia.

## Introduction

Dementia is a syndrome, with the most common type being Alzheimer's disease (Oh and Rabins, 2019). Clinically, Alzheimer's disease is mainly characterized by comprehensive dementia, including memory disorder, cognitive disorder, executive dysfunction, and personality and behavior changes, and is accompanied by mental disorder symptoms in most patients (Lyketsos et al., 2011). Although these symptoms can be temporarily relieved by detailed care and medication, there are no specific measures to prevent or cure Alzheimer's disease (Srivastava et al., 2021). Dementia mainly occurs in older age groups, and the incidence and prevalence rates increase with increasing age; this trend is more common in low- and middle-income countries and regions (Gao and Liu, 2021). It has become an increasingly serious global public health problem, imposing serious economic and disease burdens on societies and families (GBD 2016 Dementia Collaborators, 2019).

It has been reported that the global number of people with dementia has increased to 43.8 million in 2016, an increase of 117% compared with 20.3 million in 1990 (GBD 2016 Dementia Collaborators, 2019). It is estimated that, by 2050, there will be 152 million people with Alzheimer's disease and other dementias (GBD 2019 Dementia Forecasting Collaborators, 2022). Given that the onset is closely related to age, the disease burden is expected to increase with the aging population and increased life expectancy. In addition to age, a recent study in the Lancet Commission has reported other risk factors for dementia, including hypertension, obesity, diabetes, physical inactivity, hearing loss, smoking, depression, low level of education, and a low socialization frequency (Livingston et al., 2017). With progressive studies, excessive alcohol intake, brain damage, and air pollution were also added to the list of risk factors for Alzheimer's disease and dementia by 2020 (Livingston et al., 2020). Although no effective measures exist to prevent or cure the onset of dementia, growing evidence has demonstrated that more than a third of dementia cases could be prevented or delayed by managing controllable risk factors (Gauthier et al., 2016; Livingston et al., 2017). Monitoring the risk factors and the epidemiological trends of Alzheimer's disease and dementia over time is critical for the global prevention of dementia (GBD 2016 Dementia Collaborators, 2019). According to the data from 1990 to 2016 in the GBD database, the most recently published study in Lancet Neurology demonstrated a growing challenge to healthcare systems worldwide (GBD 2016 Dementia Collaborators, 2019). However, the temporal trends of the following 3 years (from 2017 to 2019) were absent. In the present study, we tracked the epidemiology trends of the past three decades from 1990 to 2019 based on the data in GBD 2019, hoping to potentially inform efforts toward preventing dementia.

The Global Burden of Disease, Injury, and Risk Factors Study (GBD) 2019 leveraged available data from the literature, medical institution records, and publicly available databases and used Bayesian regression models to perform a systematic and scientific analysis of the epidemiological data of 204 countries and regions; analyses were performed for both genders, and the GBD 2019 covered 369 diseases, with corresponding age-standardized rates of incidence, prevalence, death, and DALYs (GBD 2019 Diseases Injuries Collaborators, 2020). DALYs refer to the number of healthy years of life lost from the development of morbidity to death and are the most representative and widely used indicators of disease burden (Murray et al., 2012). The GBD 2019 also included 87 common disease risks, contributing to the understanding of dementia as a complex disease (GBD 2019 Risk Factors Collaborators, 2020).

We analyzed the changing trends of Alzheimer's disease and other dementias using data from the GBD 2019; described the global distribution characteristics of dementia by gender, age, country, and region; and comprehensively analyzed the risks of dementia in different countries and regions. We hope that this study will aid in the development of targeted policies and control of dementia to improve people's health and guide the rational allocation of medical resources.

## Materials and methods

### Data sources

The specific data analyzed in the current study were obtained from the most recently updated online Global Health Data Exchange (GHDx) Query Tool (http://ghdx.healthdata.org/gbd-results-tool) on 20 March 2020. The detailed methods followed those of the GBD 2019 (GBD 2017 DALYs HALE Collaborators, 2018; GBD 2017 Disease Injury Incidence Prevalence Collaborators, 2018; GBD 2017 Mortality Collaborators, 2018). The GBD 2019 analysis of risk factors followed the general framework of comparative risk assessment (CRA), including the inclusion of risk-outcome pairs, relative risk estimates, estimation of exposure levels and distributions, determination of counterfactual levels of exposure, calculation of population attribution scores and attribution burdens, and estimation of the mediating role of different risk factors through other risk factors (GBD 2019 Risk Factors Collaborators, 2020). The detailed methods refer to previous studies (GBD 2019 Risk Factors Collaborators, 2020). We analyzed the trends of incident cases, prevalent cases, deaths, and DALYs due to Alzheimer's disease and other dementias from 1990 to 2019 at the global, regional, and state levels. Differences among sexes and age ranges were also assessed. Age-standardized rates (ASRs) were used to prevent confounding due to age in the analysis to ensure consistency among most rates.

The sociodemographic index (SDI), which ranges from 0 to 1, is a comprehensive indicator and is calculated considering the education rate, economic situation, and total fertility rate of a country or region (HUMANOSPHERE, 2016). The larger the value of the SDI, the higher the level of social development. All 204 countries and regions were separated into five SDI groups: the high-SDI group (> 0.81), medium-high-SDI group (0.70–0.81), medium-SDI group (0.61–0.70), medium-low-SDI group (0.46–0.61), and low-SDI group (<0.46). Based on the geographic location, all the countries were also divided into 21 regions for analysis in the current study. This study was conducted using data obtained from publicly available databases, and hence no ethical approval was required.

### Statistical analysis

We described the numbers [with the associated 95% uncertainty intervals (UIs)] and the trends of incident cases, prevalent cases, deaths, and DALYs, and their corresponding ASRs (and the associated 95% UIs) for dementia by sex, age, year, SDI subregion, GBD regions, and 204 countries and regions. Since there were few cases of dementia in people under the age of 40 years, they were not included in this research, and only cases in people over 40 years of age were analyzed.

The time trends of ASRs in a specific time period are represented by the estimated annual percentage change (EAPC) (Ding et al., 2022). In short, we used the y = α + βx + ϵ regression model for these calculations, where y is ln (ASR), x is the time variable, and ε is the error term. The natural logarithm of the ASR was assumed to be linear with time; therefore, EAPC = 100 × [exp(β)−1] (Ding et al., 2022). We also used linear models to calculate 95% confidence intervals (95% CIs) for the EAPCs. If both the EAPC and its 95% CI lower bound were greater than 0, the ASR was considered to show an increasing trend. Conversely, if both the EAPC and its 95% CI upper limit were less than 0, the ASR was considered to show a decreasing trend.

Joinpoint regression analysis, performed by fitting different time periods using the simplest logarithmic model, was used to describe the changing trend over a specific time period. It allows for a more detailed assessment of different interval-specific disease variability characteristics on a global time scale. The Joinpoint regression model was mainly implemented by comparing the annual percent change (APC) and the average annual percent change (AAPC) with 0 to evaluate whether the trends of the disease rates in different segments were statistically significant. Joinpoint version 4.9.0.1 software, developed by the National Cancer Institute, was used for the Joinpoint regression analysis. All the other analyses and visualizations were carried out with GraphPad Prism 8 software and R software (version 4.1.2). A *P-*value < 0.05 was considered statistically significant.

## Results

### Incidence trends from 1990 to 2019

**Figure 1:**
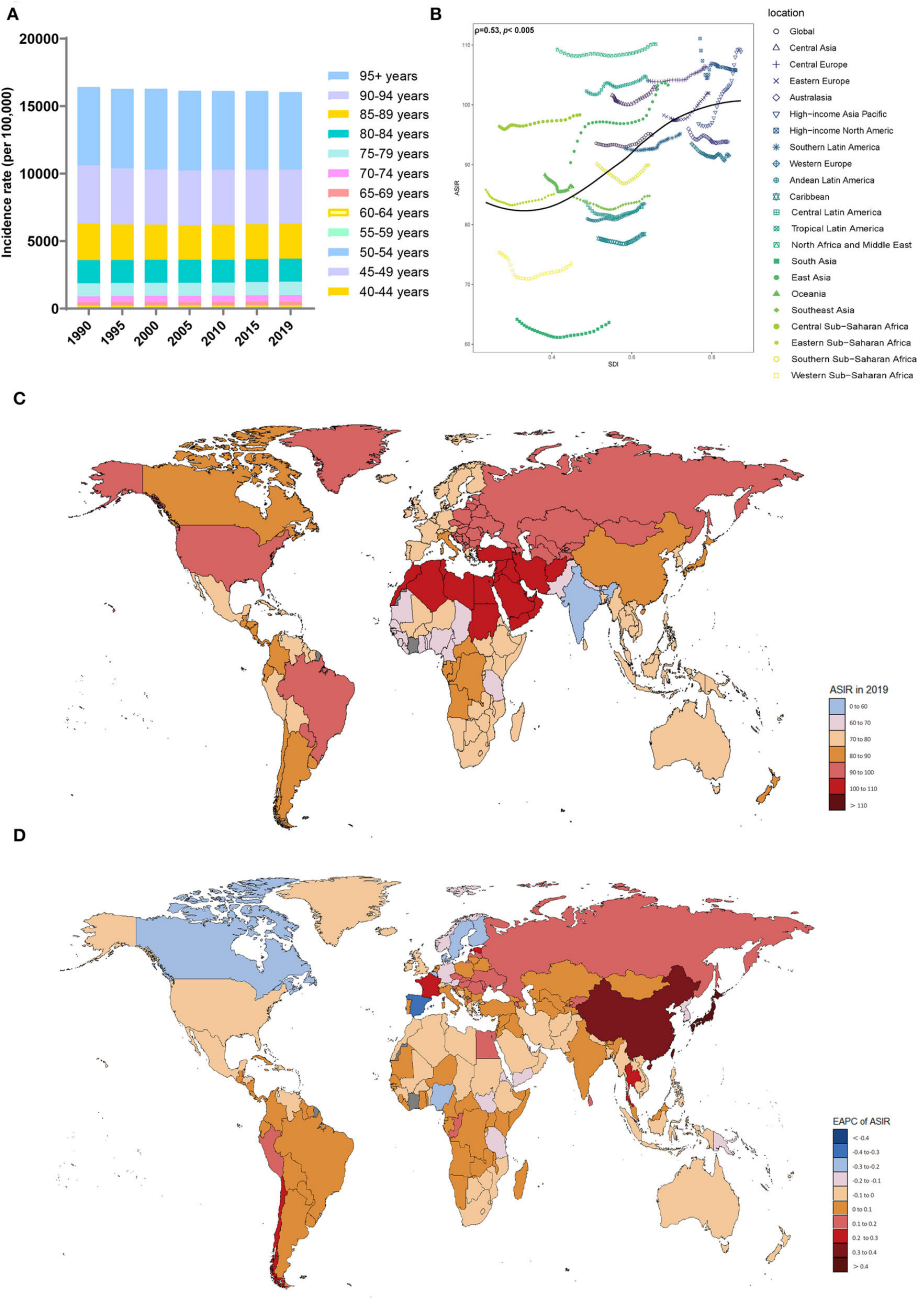
Distribution of the incidence of Alzheimer's disease and other dementias in 204 countries and territories from 1990 to 2019 by age group and SDI region. **(A)** Incidence rate in each age group. **(B)** Correlations between the ASIRs of Alzheimer's disease and other dementias and SDI regions in 2019. Associations were calculated with Pearson correlation analysis. **(C)** The ASIRs of 204 countries and territories. **(D)** EAPCs in the ASIRs in 204 countries and territories. SDI, sociodemographic index; ASIR, age-standardized incidence rate; EAPC, estimated annual percent change.

Globally, the incidence of Alzheimer's disease and other dementias increased by 147.95% from 1990 to 2019, with 2.92 million cases (95% UI: 2.49 to 3.37) in 1990 and 7.24 million cases (95% UI, 6.22 to 8.23) in 2019 (Table 1). Regarding sex, the age-standardized incidence rate (ASIR, per 100,000 population) of dementia increased in both men and women over this period (EAPC, men: 0.13; 95% CI, 0.1 to 0.16; women: 0.06; 95% CI, 0.04 to 0.08) (Table 1, Supplement Figure 1A). The overall ASIR (per 100,000 population) in men increased sharply. It is worth noting that the incidence rate of dementia increased with age (Figure 1A). From 1990 to 2019, the incidence rate in the 70–74 age group was the fastest growing over time (Supplement Figure 1B).

**Table 1:** The number of incidence cases and ASIR of Alzheimer's disease and other dementias in 1990 and 2019 and its EAPC.

	**1990**		**2019**		**1990–2019**
**Characteristics**	Incidence cases (*10^∧^6) **(95% UI)**	**ASIR (per 100,000)** **(95% UI)**	Incidence cases (*10^∧^6) **(95% UI)**	**ASIR (per 100,000)** **(95% UI)**	**EAPC of ASIR** **(95% CI)**
Global	2.92 (2.49–3.37)	93.58 (80.12–106.7)	7.24 (6.22–8.23)	94.99 (81.59–107.86)	0.06 (0.03 to 0.08)
**Gender**					
Males	1.01 (0.86–1.17)	81.03 (68.55–93)	2.69 (2.27–3.09)	83.67 (71.06–95.65)	0.13 (0.1 to 0.16)
Females	1.91 (1.63–2.2)	101.64 (87.34–115.71)	4.55 (3.93–5.16)	103.45 (89.24–117.15)	0.06 (0.04 to 0.08)
**Sociodemographic index**					
Low	0.11 (0.1–0.13)	78.7 (67.02–90.5)	0.27 (0.24–0.31)	76.97 (66.01–87.9)	−0.07 (−0.1 to −0.05)
Low-middle	0.3 (0.25–0.34)	77.31 (65.89–88.63)	0.82 (0.7–0.94)	77.07 (65.7–88.14)	−0.03 (−0.06 to −0.01)
Middle	0.61 (0.52–0.71)	88.38 (74.76–101.4)	1.9 (1.62–2.18)	93.29 (79.52–106.76)	0.11 (0.08 to 0.15)
High-middle	0.84 (0.71–0.99)	97.55 (82.44–111.71)	2.01 (1.71–2.31)	101.68 (86.58–116.12)	0.13 (0.11 to 0.16)
High	1.05 (0.9–1.21)	99.64 (86.19–113.09)	2.23 (1.93–2.51)	100.57 (87.24–113.37)	0.11 (0.08 to 0.14)
**21 GBD regions**					
Central Asia	0.04 (0.03–0.04)	101.63 (86–116.8)	0.05 (0.04–0.06)	102.74 (87.44–117.42)	0.05 (0.02 to 0.08)
Central Europe	0.13 (0.11–0.16)	104.04 (87.55–119.69)	0.24 (0.2–0.28)	106.31 (89.8–121.85)	0.09 (0.08 to 0.1)
Eastern Europe	0.23 (0.19–0.28)	98.28 (81.88–113.72)	0.36 (0.3–0.42)	101.93 (85.52–116.99)	0.17 (0.13 to 0.2)
Australasia	0.02 (0.02–0.02)	96.01 (82.52–109.28)	0.05 (0.04–0.06)	93.84 (80.46–106.76)	−0.06 (−0.07 to −0.04)
High-income Asia Pacific	0.17 (0.14–0.2)	96.86 (83.48–110.97)	0.63 (0.54–0.71)	108.9 (93.99–123.75)	0.48 (0.45 to 0.52)
High-income North America	0.42 (0.35–0.48)	111.1 (94.6–126.89)	0.74 (0.66–0.82)	105.77 (93.77–116.93)	−0.04 (−0.09 to 0)
Southern Latin America	0.04 (0.03–0.04)	92.71 (78.34–106.93)	0.08 (0.07–0.09)	95.18 (80.96–109.17)	0.1 (0.09 to 0.12)
Western Europe	0.57 (0.49–0.65)	93.49 (81.02–105.5)	1.01 (0.86–1.16)	91.52 (78.13–104.43)	−0.05 (−0.07 to −0.03)
Andean Latin America	0.01 (0.01–0.02)	80.97 (68.97–93.44)	0.04 (0.04–0.05)	83.48 (71.56–95.52)	0.11 (0.08 to 0.13)
Caribbean	0.02 (0.01–0.02)	77.69 (66.39–89.43)	0.04 (0.04–0.05)	78.42 (67.22–89.79)	0.03 (0 to 0.05)
Central Latin America	0.05 (0.05–0.06)	83.83 (71.35–96.9)	0.18 (0.16–0.21)	82.9 (70.84–95.18)	0 (−0.03 to 0.03)
Tropical Latin America	0.07 (0.06–0.08)	102.3 (88.03–116.99)	0.24 (0.21–0.27)	104.09 (90.49–118.13)	0.09 (0.07 to 0.1)
North Africa and Middle East	0.13 (0.11–0.15)	109.25 (92.75–125.03)	0.36 (0.31–0.41)	110.17 (93.94–125.62)	0.04 (0.02 to 0.06)
South Asia	0.22 (0.19–0.25)	64.17 (54.61–73.81)	0.68 (0.58–0.78)	63.63 (54.2–72.95)	−0.04 (−0.1 to 0.01)
East Asia	0.53 (0.45–0.62)	90.36 (76.22–104.02)	1.86 (1.57–2.15)	103.32 (87.6–118.29)	0.33 (0.27 to 0.39)
Oceania	0 (0–0)	88.33 (74.28–101.85)	0 (0–0)	86.26 (73.29–98.98)	−0.09 (−0.12 to −0.06)
Southeast Asia	0.15 (0.13–0.17)	84.62 (72.1–97.18)	0.4 (0.35–0.46)	84.8 (72.56–96.84)	0.02 (−0.02 to 0.06)
Central Sub-Saharan Africa	0.01 (0.01–0.01)	96.41 (83.03–110.18)	0.03 (0.03–0.04)	98.35 (85.22–112.11)	0.08 (0.07 to 0.09)
Eastern Sub-Saharan Africa	0.04 (0.03–0.05)	85.81 (73.42–98.23)	0.09 (0.08–0.11)	85.08 (73.42–96.76)	−0.02 (−0.05 to 0.02)
Southern Sub-Saharan Africa	0.02 (0.02–0.02)	90.15 (77.17–103.33)	0.04 (0.03–0.04)	89.95 (77.28–102.68)	−0.01 (−0.06 to 0.04)
Western Sub-Saharan Africa	0.04 (0.04–0.05)	75.34 (64.05–86.53)	0.09 (0.08–0.11)	73.47 (62.61–83.94)	−0.11 (−0.18 to −0.03)

ASIR, age-standardized incidence rate; EAPC, estimated annual percent change; SDI, sociodemographic index.

The ASIRs of dementia were positively related to SDI levels (ρ = 0.53, *p* < 0.005) (Figure 1B), with the ASIRs (per 100,000 population) in the high-middle-SDI (ASIR = 101.68; 95% UI, 86.58 to 116.12) and high-SDI (ASIR = 100.57; 95% UI, 87.24 to 113.37) areas being much higher than those in other SDI areas in 2019 (Table 1, Supplement Figure 1C). The ASIRs (per 100,000 population) for dementia increased most in the high-middle-SDI regions (EAPC, 0.13; 95% CI, 0.11 to 0.16), while in areas with a low-SDI (EAPC, −0.07; 95% CI, −0.1 to −0.05) and low-middle-SDI (EAPC, −0.03; 95% CI, −0.06 to −0.01), decreasing trends were observed. In 2019, the proportion of female ASIRs in all SDI regions and GBD subregions was higher than that in men (Supplement Figure 1D). In areas with a higher SDI, the incidence of dementia tended to be higher in those over 70 years old (Supplement Figure 1E).

In the 21 regions based on geographic location, the highest ASIRs (per 100,000 population) of dementia were in North Africa and the Middle East (ASIR = 110.17; 95% UI, 93.94 to 125.62), high-income Asia-Pacific (ASIR = 108.9; 95% UI, 93.99 to 123.75), and Central Europe (ASIR = 106.31; 95% UI, 89.8 to 121.85) in 2019; the lowest ASIRs were noticed in South Asia (ASIR = 63.63; 95% UI, 54.2 to 72.95) and Western Sub-Saharan Africa (ASIR = 73.47; 95% UI, 62.61 to 83.94) (Table 1). From 1990 to 2019, the ASIR (per 100,000 population) of dementia increased the most in high-income Asia-Pacific (EAPC, 0.48; 95% CI, 0.45 to 0.52), while the largest decrease occurred in Western Sub-Saharan Africa (EAPC, −0.11; 95% CI, −0.18 to −0.03). The ASIRs in the other territories have remained relatively stable.

At the country level, Turkey, Bahrain, and Iran showed the highest ASIRs (per 100,000 population) among the 204 countries and regions, while the lowest were in India, Pakistan, and Nepal (Supplement Table 1, Figure 1C). Japan and China showed the fastest increases in ASIRs (per 100,000 population) over this period (EAPC, 0.58, 95% CI, 0.53 to 0.62 for Japan; EAPC, 0.33, 95% CI, 0.27 to 0.4 for China) (Figure 1D). In most countries and regions, the ASIRs (per 100,000 population) remained relatively stable and even showed significant downward trends, including in Luxembourg (EAPC, −0.42; 95% CI, −0.52 to −0.33), Spain (EAPC, −0.31; 95% CI, −0.37 to −0.26), and Belgium (EAPC, −0.28; 95% CI, −0.3 to −0.25).

### Prevalence trends from 1990 to 2019

Globally, in 2019, the number of Alzheimer's disease and other dementia patients was 160.84% higher than that observed in 1990, which was 51.62 million (95% UI, 44.28 to 59.02 million) (Table 2). The ASPR (per 100,000 population) was 682.48 (95% UI, 585.2 to 782.73) in 2019, indicating an upward trend (EAPC, 0.2; 95% CI, 0.18 to 0.21).

**Table 2:** The number of prevalence cases and ASPR of Alzheimer's disease and other dementias in 1990 and 2019 and its EAPC.

	**1990**		**2019**		**1990–2019**
**Characteristics**	Prevalence cases (*10^∧^6) **(95% UI)**	**ASPR (per 100,000)** **(95% UI)**	Prevalence cases (*10^∧^6) **(95% UI)**	**ASPR (per 100,000)** **(95% UI)**	**EAPC of ASPR (95% CI)**
Global	19.79 (16.93–22.79)	645.89 (552.86–743.62)	51.62 (44.28–59.02)	682.48 (585.2–782.73)	0.2 (0.18 to 0.21)
**Gender**					
Males	6.77 (5.71–7.83)	547.5 (463.19–631.56)	18.7 (15.78–21.51)	585.87 (497.37–672.86)	0.26 (0.24 to 0.28)
Females	13.02 (11.17–14.96)	703.9 (605.24–809.19)	32.92 (28.43–37.72)	748.15 (646.36–856.85)	0.21 (0.2 to 0.23)
**Sociodemographic index**					
Low	0.75 (0.64–0.87)	515.21 (440.9–594.89)	1.83 (1.56–2.1)	514.75 (440.15–591.04)	0.01 (0 to 0.03)
Low-middle	1.96 (1.68–2.26)	513.81 (439.5–593.94)	5.64 (4.8–6.5)	530.64 (453.38–611.28)	0.09 (0.08 to 0.1)
Middle	4.06 (3.45–4.7)	589.56 (504.16–680.86)	13.37 (11.32–15.46)	666.73 (565.41–768.83)	0.32 (0.28 to 0.36)
High-middle	5.75 (4.85–6.66)	682.34 (577.51–789.53)	14.56 (12.35–16.87)	739.18 (626.68–855.07)	0.26 (0.24 to 0.28)
High	7.26 (6.22–8.34)	692.75 (598.34–792.76)	16.2 (14.05–18.36)	721.43 (627.54–817.7)	0.23 (0.2 to 0.26)
**21 GBD regions**					
Central Asia	0.26 (0.22–0.31)	699.37 (589.17–811.44)	0.34 (0.29–0.4)	716.26 (605.71–834.99)	0.1 (0.08 to 0.13)
Central Europe	0.92 (0.76–1.08)	735.33 (615.6–856.54)	1.74 (1.45–2.03)	761.23 (636.83–884.61)	0.14 (0.13 to 0.14)
Eastern Europe	1.58 (1.31–1.87)	683.38 (568.49–801.74)	2.52 (2.11–2.97)	717.09 (600.77–837.07)	0.23 (0.19 to 0.26)
Australasia	0.15 (0.13–0.17)	681.73 (584.78–782.18)	0.38 (0.32–0.44)	674.23 (574.91–778.88)	−0.02 (−0.03 to −0.01)
High-income Asia Pacific	1.11 (0.96–1.29)	649.53 (556.92–750.35)	4.58 (3.88–5.31)	773.2 (662.42–891.86)	0.69 (0.64 to 0.73)
High-income North America	2.98 (2.52–3.45)	795.59 (675.14–916.53)	5.44 (4.85–5.97)	773.57 (690.32–849.66)	0.06 (0 to 0.12)
Southern Latin America	0.25 (0.21–0.3)	637.38 (535.38–739.47)	0.58 (0.49–0.68)	670.68 (567.9–776.55)	0.19 (0.18 to 0.2)
Western Europe	3.89 (3.36–4.43)	646.9 (562.24–731.98)	7.32 (6.23–8.48)	651.29 (557.74–752.35)	0.04 (0.02 to 0.05)
Andean Latin America	0.09 (0.08–0.1)	520.49 (448.14–597.87)	0.29 (0.25–0.34)	558.72 (481.3–642.17)	0.25 (0.23 to 0.26)
Caribbean	0.12 (0.1–0.14)	514.03 (442.79–590.87)	0.28 (0.24–0.32)	530.93 (457.42–607.2)	0.11 (0.1 to 0.12)
Central Latin America	0.36 (0.31–0.41)	541.69 (464.48–626.82)	1.21 (1.04–1.38)	546.85 (471.02–628.34)	0.08 (0.06 to 0.11)
Tropical Latin America	0.49 (0.42–0.56)	714.71 (617.47–818.28)	1.74 (1.5–1.98)	767.53 (663.42–876.09)	0.34 (0.29 to 0.38)
North Africa and Middle East	0.87 (0.74–1.01)	754.7 (640.49–870.78)	2.49 (2.12–2.86)	777.63 (660.8–895.96)	0.12 (0.11 to 0.13)
South Asia	1.45 (1.24–1.68)	421.73 (360.05–485.43)	4.64 (3.93–5.34)	428.42 (365.02–493.98)	0.04 (0 to 0.09)
East Asia	3.47 (2.9–4.04)	609.05 (515.11–707.65)	13.57 (11.39–15.79)	781.43 (659.97–904.1)	0.65 (0.56 to 0.73)
Oceania	0.01 (0.01–0.01)	581.21 (492–676.1)	0.02 (0.02–0.03)	575.09 (485.96–666.95)	−0.05 (−0.07 to −0.03)
Southeast Asia	1.02 (0.87–1.18)	566.26 (483.98–651.08)	2.79 (2.39–3.2)	590.55 (505.03–677.25)	0.17 (0.14 to 0.2)
Central Sub-Saharan Africa	0.08 (0.07–0.1)	665.37 (567.54–766.03)	0.22 (0.19–0.25)	695.87 (596.01–796.94)	0.17 (0.16 to 0.18)
Eastern Sub-Saharan Africa	0.26 (0.23–0.31)	570.52 (489.37–655.73)	0.62 (0.53–0.71)	574.66 (495.93–657.82)	0.04 (0.02 to 0.07)
Southern Sub-Saharan Africa	0.13 (0.11–0.15)	601.78 (515.22–692.95)	0.26 (0.22–0.3)	607.44 (520.21–699.81)	0.03 (−0.02 to 0.09)
Western Sub-Saharan Africa	0.29 (0.25–0.34)	481.44 (411.98–554.82)	0.61 (0.52–0.69)	469.82 (402.13–539.35)	−0.1 (−0.19 to −0.02)

ASPR, age-standardized prevalence rate; EAPC, estimated annual percent change; SDI, sociodemographic index.

**Figure 2:**
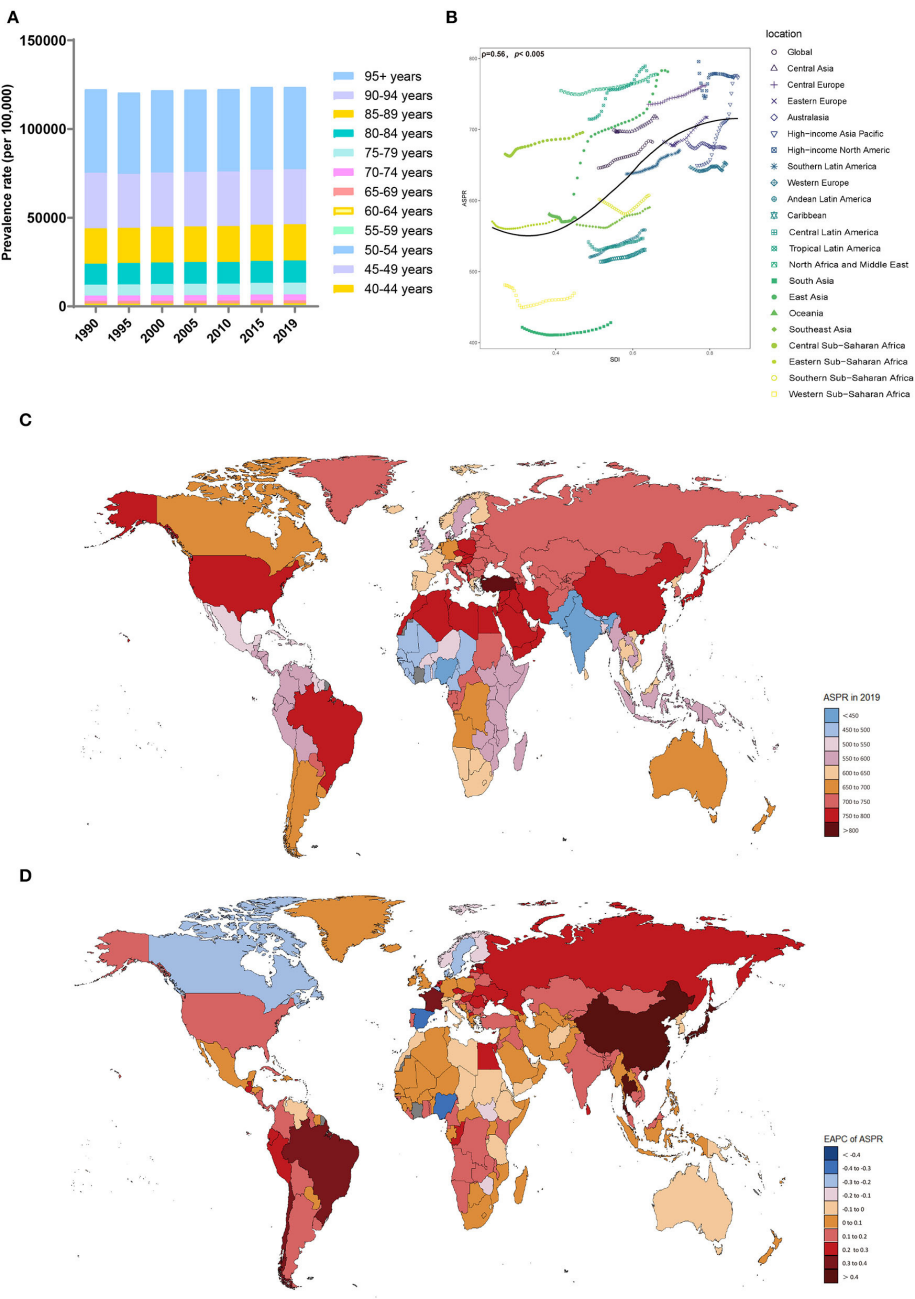
Distribution of the prevalence of Alzheimer's disease and other dementias in 204 countries and territories from 1990 to 2019 by age group and SDI region. **(A)** Prevalence rate in each age group; **(B)** Correlations between the ASPRs of Alzheimer's disease and other dementias and SDI regions in 2019; associations were calculated with Pearson correlation analysis; **(C)** The ASPRs in 204 countries and territories; **(D)** EAPCs in the ASPRs in 204 countries and territories. SDI, sociodemographic index; ASPR, age-standardized prevalence rate; EAPC, estimated annual percent change.

The ASPRs (per 100,000 population) in all of the SDI regions showed an upward trend, with the highest in the high-middle-SDI (739.18, 95% UI, 626.68 to 855.07) region, while ASPRs in the middle-SDI region showed the fastest increase (EAPC, 0.32, 95% CI, 0.28 to 0.36) (Table 2, Supplement Figure 2C). Compared with men, women had a higher ASPR (per 100,000 population) (Table 2, Supplement Figures 2A,D). However, there was a larger upward trend in the male ASPR (per 100,000 population) (EAPC, 0.26; 95% CI, 0.24 to 0.28). The prevalence rate increased with age (Figure 2A). From 1990 to 2019, the prevalence rate in the 80–84 age group was the fastest growing over time (Supplement Figure 2B). Notably, younger patients were mainly concentrated in lower SDI regions (Supplement Figure 2E).

The region with the highest ASPR (per 100,000 population) in 2019 was East Asia (781.43, 95% UI, 659.97 to 904.1), and the region with the lowest was South Asia (428.42, 95% UI, 365.02–493.98). In most territories, particularly high-income Asia-Pacific (EAPC, 0.69; 95% CI, 0.64 to 0.73), the ASPR (per 100,000 population) showed upward trends. Only Australia (EAPC, −0.02; 95% CI, −0.03 to −0.01), Oceania (EAPC, −0.05; 95% CI, −0.07 to −0.03), and Western Sub-Saharan Africa (EAPC, −0.1; 95% CI, −0.19 to −0.02) showed a slight downward trend. The ASPRs (per 100,000 population) were also positively related to the SDI values among the 21 regions in 2019 (ρ = 0.56, *p* < 0.005) (Figure 2B).

In 2019, the highest ASPRs (per 100,000 population) were noticed in Turkey, Bahrain, and Kuwait, while the lowest ASPRs (per 100,000 population) were observed in India, Nigeria, and Pakistan (Supplement Table 2, Figure 2C). The ASPRs (per 100,000 population) in Taiwan, China (EAPC, 0.78; 95% CI, 0.64 to 0.92), Japan (EAPC, 0.77; 95% CI, 0.71 to 0.83), and China (EAPC, 0.66; 95% CI, 0.57 to 0.75) showed the fastest upward trend (Figure 2D). The ASPRs (per 100,000 population) in Luxembourg (EAPC, −0.43; 95% CI, −0.54 to −0.31), Nigeria (EAPC, −0.34; 95% CI, −0.48 to −0.19), and Spain (EAPC, −0.32; 95% CI, −0.38 to −0.26) showed a significant downward trend.

### Death trends from 1990 to 2019

**Figure 3:**
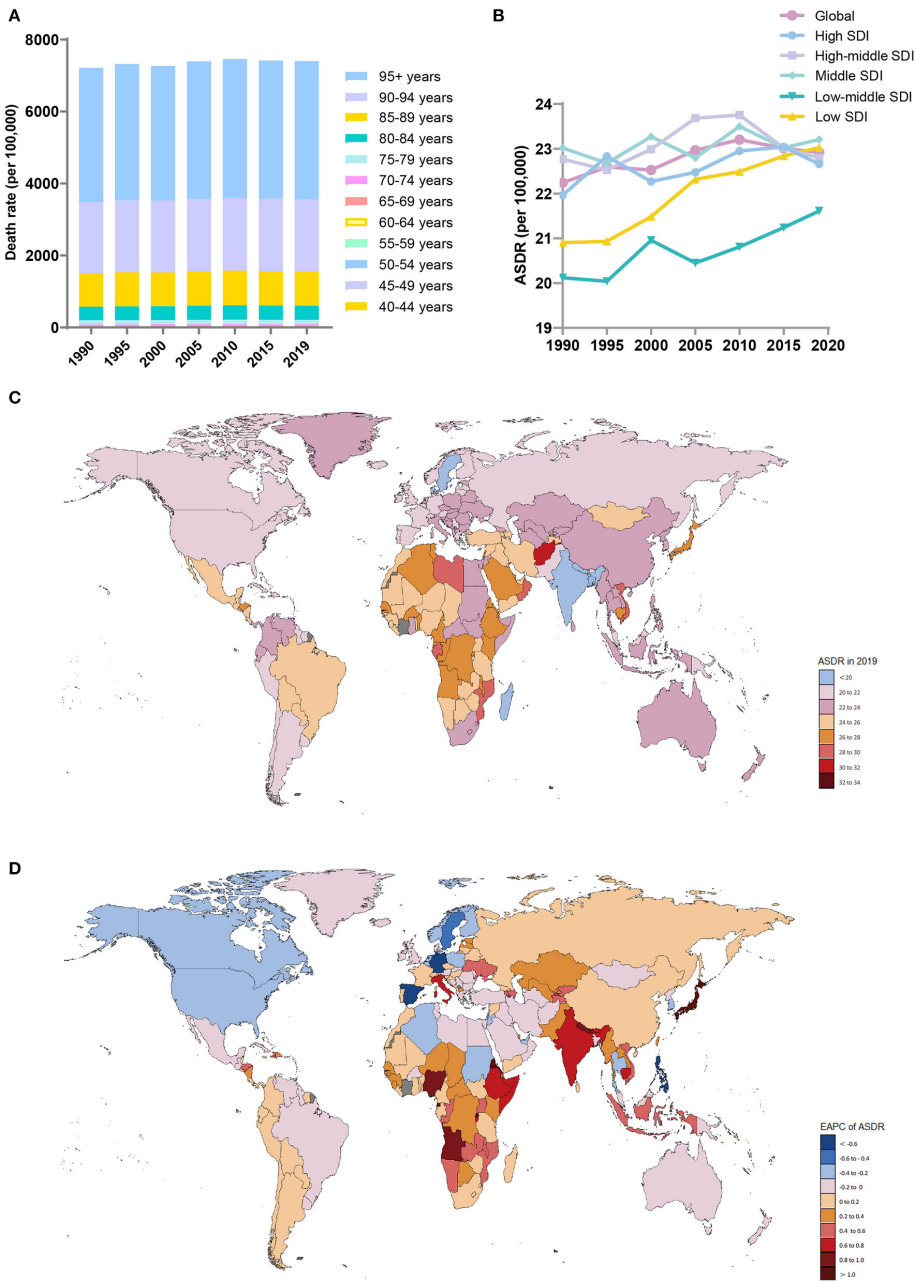
Distribution of deaths due to Alzheimer's disease and other dementias in 204 countries and territories from 1990 to 2019 by age group and SDI region. **(A)** Death rate in each age group; **(B)** Trends of the ASDRs in the SDI region; **(C)** ASDRs in 204 countries and territories; **(D)** EAPCs of the ASDRs in 204 countries and territories. SDI, sociodemographic index; ASDR, age-standardized death rate; EAPC, estimated annual percent change.

Global deaths due to Alzheimer's disease and other dementias increased from 0.56 million (95% UI, 0.14 to 1.55) in 1990 to 1.62 million (95% UI, 0.41 to 4.21) in 2019, nearly tripling in 30 years (Table 3). The age-standardized death rate (ASDR) (per 100,000 population) also increased (EAPC, 0.13, 95% CI, 0.1 to 0.15). From 1990 to 2019, the ASDRs (per 100,000 population) increased in both men and women (EAPC, men: 0.2; 95% CI, 0.18 to 0.23; women: 0.12; 95% CI, 0.09 to 0.15) (Table 3, Supplement Figure 3A). In 2019, the overall ASDR proportions (per 100,000 population) in women in each SDI region and GBD region were higher than those in men (Supplement Figure 3D). Across the age groups, the death rate increased with age (Figure 3A). From 1990 to 2019, death cases in > 95 age group showed the fastest growth over time (Supplement Figure 3B).

**Table 3:** The number of deaths and ASDR of Alzheimer's disease and other dementias in 1990 and 2019 and its EAPC.

	**1990**		**2019**		**1990–2019**
**Characteristics**	Death cases (*10^∧^6) **(95% UI)**	**ASDR (per 100,000)** **(95% UI)**	Death cases (*10^∧^6) **(95% UI)**	**ASDR (per 100,000)** **(95% UI)**	**EAPC of ASDR from 1990 to 2019** **(95% CI)**
Global	0.56 (0.14–1.55)	22.24 (5.5–59.98)	1.62 (0.41–4.21)	22.92 (5.83–59.2)	0.13 (0.1 to 0.15)
**Gender**					
Males	0.18 (0.04–0.51)	19.71 (4.7–54.97)	0.56 (0.14–1.51)	20.71 (5.03–55.21)	0.2 (0.18 to 0.23)
Females	0.38 (0.09–1.04)	23.49 (5.86–62.54)	1.06 (0.27–2.71)	24.19 (6.24–61.53)	0.12 (0.09 to 0.15)
**Sociodemographic index**					
Low	0.02 (0–0.06)	20.9 (4.93–58.1)	0.06 (0.02–0.17)	23.03 (5.64–62.49)	0.4 (0.36 to 0.43)
Low-middle	0.05 (0.01–0.15)	20.12 (4.77–55.72)	0.19 (0.05–0.5)	21.61 (5.22–56.36)	0.26 (0.23 to 0.3)
Middle	0.12 (0.03–0.33)	23.02 (5.52–63.87)	0.39 (0.09–1.03)	23.21 (5.69–60.43)	0.09 (0.06 to 0.12)
High-middle	0.15 (0.04–0.42)	22.53 (5.54–60.52)	0.42 (0.1–1.12)	22.8 (5.68–60.14)	0.09 (0.03 to 0.16)
High	0.22 (0.05–0.58)	21.96 (5.49–57.5)	0.56 (0.15–1.39)	22.66 (5.89–56.63)	0.1 (0.06 to 0.14)
**21 GBD regions**					
Central Asia	0.01 (0–0.02)	21.61 (5.35–59.48)	0.01 (0–0.02)	23.45 (5.72–64.46)	0.26 (0.22 to 0.31)
Central Europe	0.02 (0.01–0.07)	23.43 (5.7–64.73)	0.05 (0.01–0.13)	23.02 (5.54–59.38)	−0.1 (−0.12 to −0.08)
Eastern Europe	0.04 (0.01–0.11)	20.29 (4.82–55.98)	0.07 (0.02–0.2)	21.55 (5.25–57.33)	0.2 (0.16 to 0.24)
Australasia	0 (0–0.01)	22.57 (5.6–59.73)	0.01 (0–0.03)	22.08 (5.71–56.53)	−0.09 (−0.11 to −0.07)
High-income Asia Pacific	0.03 (0.01–0.09)	21.86 (5.4–58.45)	0.18 (0.05–0.44)	27 (7.46–65.44)	0.96 (0.81 to 1.12)
High-income North America	0.08 (0.02–0.21)	21.38 (5.41–56.38)	0.16 (0.04–0.4)	20.87 (5.34–52.13)	−0.26 (−0.35 to −0.17)
Southern Latin America	0.01 (0–0.02)	20.86 (5.05–56.22)	0.02 (0–0.05)	21.03 (5.13–56.08)	0.08 (0.06 to 0.11)
Western Europe	0.12 (0.03–0.32)	22.27 (5.52–57.2)	0.26 (0.07–0.68)	21.31 (5.37–54.62)	−0.17 (−0.2 to −0.14)
Andean Latin America	0 (0–0.01)	21.48 (5.26–58.33)	0.01 (0–0.03)	21.76 (5.35–55.53)	0.08 (0.05 to 0.1)
Caribbean	0 (0–0.01)	20.59 (5.04–55.72)	0.01 (0–0.03)	20.75 (5.26–51.95)	0.05 (0.04 to 0.07)
Central Latin America	0.01 (0–0.04)	23.44 (5.76–63.72)	0.05 (0.01–0.13)	23.84 (6.04–60.61)	0.02 (0 to 0.05)
Tropical Latin America	0.01 (0–0.04)	26.64 (6.57–71.11)	0.06 (0.01–0.14)	25.57 (6.51–66.21)	−0.08 (−0.11 to −0.05)
North Africa and Middle East	0.02 (0.01–0.07)	26.12 (6.35–72.64)	0.07 (0.02–0.19)	25.52 (6.34–67.06)	−0.06 (−0.09 to −0.02)
South Asia	0.04 (0.01–0.11)	16.79 (3.83–48.06)	0.16 (0.04–0.44)	19.17 (4.62–52.06)	0.44 (0.35 to 0.53)
East Asia	0.1 (0.02–0.27)	23.3 (5.39–63.68)	0.33 (0.08–0.89)	23.19 (5.63–61.19)	0.14 (0.06 to 0.22)
Oceania	0 (0–0)	22.17 (5.12–60.7)	0 (0–0)	21.52 (5.08–58.96)	−0.14 (−0.17 to −0.11)
Southeast Asia	0.03 (0.01–0.09)	22.83 (5.48–62.35)	0.09 (0.02–0.24)	23.62 (5.72–60.72)	0.12 (0.1 to 0.14)
Central Sub-Saharan Africa	0 (0–0.01)	23.66 (5.51–67.36)	0.01 (0–0.02)	26.57 (6.67–71.45)	0.43 (0.4 to 0.45)
Eastern Sub-Saharan Africa	0.01 (0–0.02)	22.95 (5.4–62.79)	0.02 (0.01–0.06)	26.25 (6.52–69.47)	0.54 (0.5 to 0.58)
Southern Sub-Saharan Africa	0 (0–0.01)	21.95 (5.17–59.26)	0.01 (0–0.02)	22.87 (5.49–62.53)	0.12 (0.07 to 0.17)
Western Sub-Saharan Africa	0.01 (0–0.03)	22.9 (5.5–65.94)	0.03 (0.01–0.07)	25.63 (6.31–68.48)	0.51 (0.44 to 0.58)

ASDR, age-standardized death rate; EAPC, estimated annual percent change; SDI, sociodemographic index.

In 2019, the ASDRs (per 100,000 population) were highest in the middle-SDI (ASDR = 23.21; 95% UI, 5.69 to 60.43) and low-SDI (ASDR = 23.03; 95% UI, 5.64 to 62.49) regions (Table 3, Figure 3B). The low-SDI regions had the largest increase in the ASDR (per 100,000 population) (EAPC, 0.4; 95% CI, 0.36 to 0.43). There was no obvious relationship between the SDI and the ASDR (per 100,000 population) values in each region (Supplement Figure 3C). Supplement Figure 5 shows the joinpoint regression analysis of the ASDRs (per 100,000 population) for each SDI region from 1990 to 2019. The global ASDR (per 100,000 population) increased significantly from 1990 to 2009 and then decreased significantly after 2010. Overall, the ASDR (per 100,000 population) in the low-SDI regions remained stable from 1990 to 1996 and increased sharply from 1996 to 2019. The high-SDI regions experienced a significant increase in the ASDR (per 100,000 population) from 1990 to 1995 and from 2000 to 2011 and a sharp decline from 1995 to 2000 and from 2015 to 2019; the overall increase in the ASDR (per 100,000 population) was modest (EAPC, 0.1; 95% CI, 0.056 to 0.139). The lower the SDI, the higher the proportion of younger people who died due to dementia (Supplement Figure 3E).

The regions with the highest ASDRs (per 100,000 population) of dementia in 2019 were high-income Asia-Pacific (27; 95% UI, 7.46 to 65.44), Central Sub-Saharan Africa (26.57; 95% UI, 6.67 to 71.45), and Eastern Sub-Saharan Africa (26.25; 95% UI, 6.52 to 69.47), while the lowest ASDRs were in South Asia (19.17; 95% UI, 4.62 to 52.06), the Caribbean (20.75; 95% UI, 5.26 to 51.95) and high-income North America (20.87; 95% UI, 5.34 to 52.13) (Table 3). The ASDRs (per 100,000 population) of dementia increased the most in high-income Asia-Pacific (EAPC, 0.96; 95% CI, 0.81 to 1.12), high-income North America (EAPC, −0.26; 95% CI, −0.35 to −0.17), and Western Europe (EAPC, −0.17; 95% CI, −0.2 to −0.14) decreased the most.

The countries with the highest ASDRs (per 100,000 population) of dementia in 2019 were Kiribati and Afghanistan, while the countries with the lowest ASDRs (per 100,000 population) were Bangladesh, India, and Luxembourg (Supplement Table 3, Figure 3C). The countries with the largest increase in the ASDRs (per 100,000 population) of dementia were Eritrea (EAPC, 1.37,; 95% CI, 1.19 to 1.55) and Rwanda (EAPC, 1.14; 95% CI, 0.99 to 1.28) (Figure 3D). Most countries, notably Germany (EAPC, −0.94, 95% CI, −1.08 to −0.81), Guam (EAPC, −0.71, 95% CI, −0.82 to −0.61), and the Philippines (EAPC, −0.7, 95% CI, −0.89 to −0.51), showed a downward trend in the ASDRs (per 100,000 population).

### DALYs trends from 1990 to 2019

**Figure 4:**
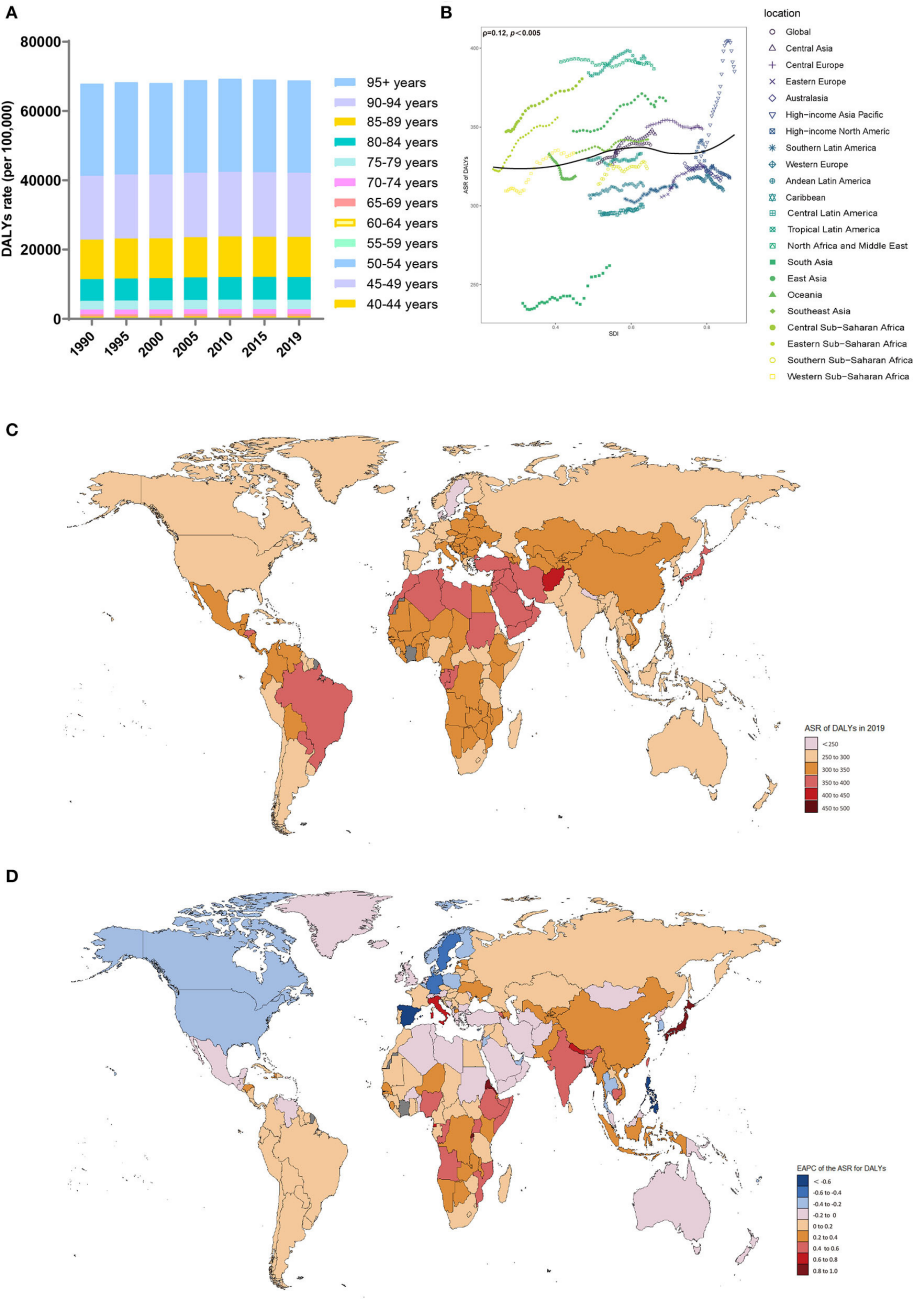
Distribution of DALYs due to Alzheimer's disease and other dementias in 204 countries and territories from 1990 to 2019 by age group and SDI region. **(A)** DALYs rate in each age group; **(B)** Correlations between the ASR of DALYs due to Alzheimer's disease and other dementias and SDI regions in 2019; associations were calculated with Pearson correlation analysis; **(C)** ASR of DALYs of 204 countries and territories; **(D)** EAPCs in the ASR of DALYs in 204 countries and territories. DALYs, disability-adjusted life-years; SDI, sociodemographic index; ASR, age-standardized rate; EAPC, estimated annual percent change.

Between 1990 and 2019, the number of DALYs due to Alzheimer's disease and other forms of dementia worldwide increased from 9.66 million (95% UI, 4.23 to 21.37) to 25.28 million (95% UI, 11.2 to 54.56) (Table 4). The ASR also increased (EAPC, 0.15; 95% CI, 0.13 to 0.16). In 2019, the burden of disease attributed to dementia was consistently higher in women than in men. However, the increase was larger in men (EAPC, men: 0.21; 95% CI, 0.2 to 0.23; women: 0.15; 95% CI, 0.13 to 0.17) (Table 4, Supplement Figure 4A). Among the age groups, the DALYs rate increased with age (Figure 4A). From 1990 to 2019, the number of DALYs in the 80–84 age group showed the fastest growth over time (Supplement Figure 4B).

**Table 4:** The DALYs cases and ASR of DALYs of Alzheimer's disease and other dementias in 1990 and 2019 and its EAPC.

	**1990**		**2019**		**1990–2019**
**Characteristics**	DALYs (*10^∧^6) **(95% UI)**	**ASR of DALYs (per 100,000)** **(95% UI)**	DALYs (*10^∧^6) **(95% UI)**	**ASR of DALYs (per 100,000)** **(95% UI)**	**EAPC of ASR of DALYs** **(95% CI)**
Global	9.66 (4.23–21.37)	326.71 (143.33–731.03)	25.28 (11.2–54.56)	338.64 (151.02–731.27)	0.15 (0.13 to 0.16)
**Gender**					
Males	3.39 (1.45–7.83)	289.24 (123.24–660.87)	9.37 (4.02–21.1)	304.47 (130.97–679.76)	0.21 (0.2 to 0.23)
Females	6.27 (2.77–13.77)	347.87 (154.52–765.02)	15.9 (7.16–33.81)	361.2 (162.8–767.72)	0.15 (0.13 to 0.17)
**Sociodemographic index**					
Low	0.39 (0.16–0.91)	295.19 (121.35–694.71)	1.04 (0.43–2.45)	315.76 (129.02–742.9)	0.28 (0.26 to 0.3)
Low-middle	1.01 (0.43–2.41)	287.22 (118.6–672.6)	3.07 (1.28–7.06)	304.76 (126.52–702.3)	0.2 (0.18 to 0.23)
Middle	2.2 (0.92–5.13)	335.33 (139.18–770.43)	6.69 (2.84–15.09)	346.11 (148.62–770.18)	0.14 (0.12 to 0.16)
High-middle	2.71 (1.19–5.92)	335.16 (148.39–736.45)	6.81 (3.08–14.82)	348.46 (157.71–754.37)	0.18 (0.13 to 0.22)
High	3.35 (1.53–7.31)	324.07 (147.34–705.64)	7.66 (3.56–15.74)	332.4 (155–688.29)	0.11 (0.08 to 0.13)
**21 GBD regions**					
Central Asia	0.12 (0.06–0.27)	326.32 (149.38–721.24)	0.16 (0.07–0.35)	345.47 (155.28–775.08)	0.19 (0.16 to 0.21)
Central Europe	0.42 (0.19–0.94)	349.87 (156.52–778.96)	0.79 (0.37–1.69)	348.75 (160.87–734.47)	−0.04 (−0.05 to −0.02)
Eastern Europe	0.68 (0.31–1.51)	305.52 (140.31–679.46)	1.14 (0.53–2.49)	324.8 (149.89–705.23)	0.23 (0.21 to 0.25)
Australasia	0.07 (0.03–0.15)	326.93 (147.01–726.32)	0.18 (0.08–0.39)	319.88 (146.31–681.98)	−0.09 (−0.11 to −0.07)
High-income Asia Pacific	0.54 (0.24–1.18)	322.6 (143.99–710.51)	2.37 (1.1–4.85)	385.38 (179.3–791.75)	0.81 (0.7 to 0.92)
High-income North America	1.24 (0.59–2.63)	330.78 (158–700.92)	2.27 (1.1–4.7)	317.66 (154.02–653.15)	−0.22 (−0.27 to −0.17)
Southern Latin America	0.12 (0.05–0.26)	304.44 (137–674.68)	0.27 (0.12–0.59)	311.86 (142.12–674.17)	0.13 (0.11 to 0.14)
Western Europe	1.85 (0.84–4.05)	317.02 (141.51–693.85)	3.58 (1.64–7.58)	309.71 (141.53–663.69)	−0.08 (−0.1 to −0.06)
Andean Latin America	0.05 (0.02–0.12)	306.69 (127.33–718.29)	0.16 (0.07–0.36)	312.67 (133.67–691.68)	0.09 (0.08 to 0.11)
Caribbean	0.07 (0.03–0.15)	295.98 (122.92–684.98)	0.16 (0.07–0.34)	299.45 (126.93–661.71)	0.06 (0.05 to 0.07)
Central Latin America	0.21 (0.09–0.49)	329.17 (133.63–765.75)	0.73 (0.3–1.63)	333.23 (136.24–747.25)	0.03 (0.01 to 0.04)
Tropical Latin America	0.24 (0.1–0.55)	384.41 (163.84–871.32)	0.87 (0.38–1.91)	390.88 (172.01–855.99)	0.11 (0.08 to 0.15)
North Africa and Middle East	0.44 (0.19–0.98)	391.32 (170.34–883.07)	1.21 (0.53–2.67)	386.96 (171.95–848.51)	−0.03 (−0.04 to −0.01)
South Asia	0.73 (0.3–1.73)	238.2 (96.13–575.6)	2.62 (1.06–6.23)	262.09 (105.55–617.38)	0.32 (0.25 to 0.39)
East Asia	1.91 (0.79–4.46)	347.44 (143.95–798.77)	6.2 (2.78–13.58)	366.61 (164.75–789.1)	0.25 (0.21 to 0.3)
Oceania	0.01 (0–0.01)	332.94 (136.7–765.18)	0.01 (0.01–0.03)	318.77 (133.77–726.91)	−0.18 (−0.21 to −0.15)
Southeast Asia	0.57 (0.23–1.32)	333.58 (136.46–764.32)	1.55 (0.63–3.43)	342.18 (141.51–765.11)	0.09 (0.07 to 0.1)
Central Sub-Saharan Africa	0.04 (0.02–0.09)	347.02 (149.92–796.7)	0.11 (0.05–0.26)	380.57 (165.07–857.23)	0.35 (0.33 to 0.37)
Eastern Sub-Saharan Africa	0.14 (0.06–0.31)	323.31 (135.15–746.55)	0.35 (0.15–0.82)	356 (144.97–829.93)	0.4 (0.37 to 0.43)
Southern Sub-Saharan Africa	0.06 (0.03–0.15)	313.71 (135.09–712.18)	0.13 (0.06–0.3)	323.81 (138.29–740.97)	0.1 (0.06 to 0.13)
Western Sub-Saharan Africa	0.17 (0.07–0.42)	309.39 (124–769.07)	0.4 (0.16–0.93)	333.53 (129.48–774.76)	0.35 (0.3 to 0.4)

DALYs, disability-adjusted life-years; ASR, age-standardized rate; EAPC, estimated annual percent change; SDI, sociodemographic index.

From 1990 to 2019, the ASR of DALYs (per 100,000 population) due to dementia trended upward in all SDI regions, especially in the low-SDI regions (EAPC, 0.28; 95% CI, 0.26 to 0.3) (Table 4, Supplement Figure 4C). In 2019, the DALY ASR (per 100,000 population) was highest in the high-middle-SDI regions (ASR of DALYs = 348.46; 95% UI, 157.71 to 754.37). In 2019, the proportion of the ASR of DALYs in women was higher than that in men in all SDI and GBD territories (Supplement Figure 4D). Patients >70 years of age accounted for a considerable percentage of DALYs due to dementia, and the proportion increased with increasing SDI (Supplement Figure 4E).

In 2019, the GBD region with the highest ASR of DALYs (per 100,000 population) due to dementia was tropical Latin America (ASR of DALYs = 390.88; 95% UI, 172.01 to 855.99), while the region with the lowest ASR of DALYs was South Asia (ASR of DALYs = 262.09; 95% UI, 105.55 to 617.38) (Table 4). The ASR of DALYs due to dementia increased in most regions from 1990 to 2019, with the largest increase in high-income Asia-Pacific (EAPC, 0.81; 95% CI, 0.7 to 0.92). The ASR of DALYs declined in high-income North America (EAPC, −0.22; 95% CI, −0.27 to −0.17). The ASRs of DALYs in each region in 2019 were positively related to the SDI (ρ = 0.12, *p* < 0.005) (Figure 4B).

In 2019, the highest ASR of DALYs (per 100,000 population) was observed in Kiribati, Afghanistan, and Oman (Supplement Table 4, Figure 4C). In contrast, the countries with the lowest ASR of DALYs (per 100,000 population) were India and Bangladesh. Since 1990, the ASR of DALYs for Eritrea (EAPC, 0.94, 95% CI: 0.83 to 1.05) and Japan (EAPC, 0.93, 95% CI: 0.81 to 1.05) increased significantly (Figure 4D). In contrast, Spain (EAPC, −0.63, 95% CI: −0.77 to −0.48), the Philippines (EAPC, −0.6, 95% CI: −0.77 to −0.44), and Germany (EAPC, −0.58, 95% CI: −0.66 to −0.5) showed clear downward trends.

### Risk factors

Based on GBD2019, high fasting blood glucose, high body mass index [BMI], and smoking were the major risk factors for dementia. The ASDR and DALYs (per 100,000 population) caused by smoking were the highest, at 2.91 (95% UI, 0.64 to 8.06) and 51.30 (95% UI, 20.50 to 116.23), respectively (Supplement Table 5).

**Figure 5:**
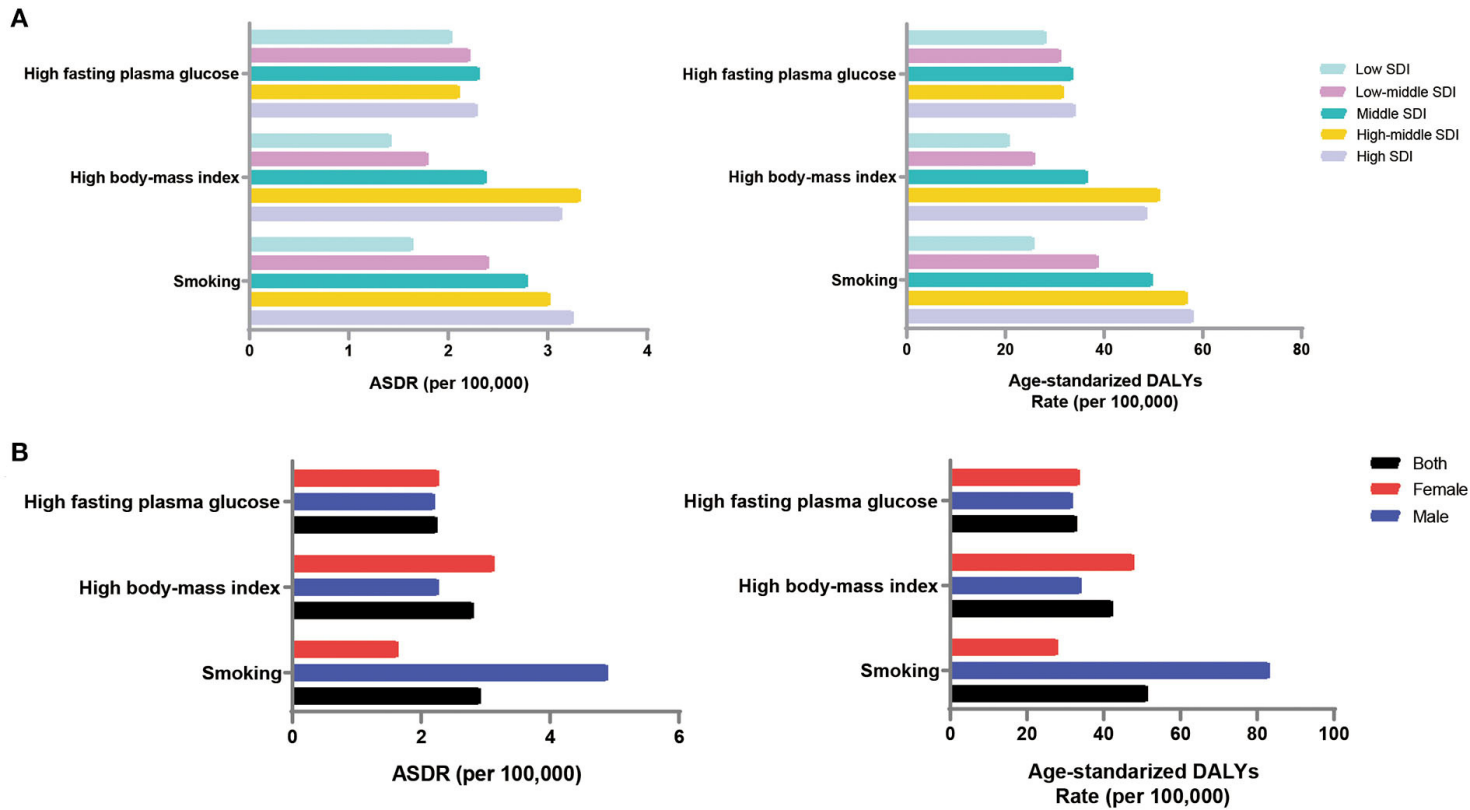
Impact of risk factors on the disease burden of Alzheimer's disease by SDI region and gender, 2019. **(A)** Contribution of three risk factors for death and DALYs in different SDI regions; **(B)** Contribution of three risk factors to death and DALYs in both sexes. ASDR, age-standardized death rate; DALYs, disability-adjusted life-years; SDI, sociodemographic index.

In the high-middle-SDI group, risk factors had the highest impact on the dementia burden; the ASDR (per 100,000 population) was 7.49 (95% UI, 1.63 to 22.15), and the ASR of DALYs (per 100,000 population) was 122.65 (95% UI, 48.42 to 302.74) (Supplement Table 5). This was followed by the high-SDI group, with an ASDR (per 100,000 population) of 7.56 (95% UI, 1.69 to 21.41) and ASR of DALYs (per 100,000 population) of 120.88 (95% UI, 49.78 to 282.72). Risk factors had the least impact on the disease burden in the low-SDI regions, with a corresponding ASDR and ASR of DALYs (per 100,000 population) of 4.71 (95% UI, 0.96 to 14.78) and 68.65 (95% UI, 22.57 to 183.31), respectively. Smoking contributed the most to the disease burden in areas with the high-SDI group (Figure 5A). For the low-SDI region, the ASDR and ASR of DALYs (per 100,000 population) attributable to high fasting plasma glucose were the highest. The burden of disease attributable to smoking increased gradually with increasing SDI.

Men were more affected by risk factors than women, with an ASDR of 8.14 (95% UI, 1.91 to 23.50) and ASR of DALYs (per 100,000 population) of 128.80 (95% UI, 51.81 to 303.90) (Supplement Table 5). Men are more susceptible to behavioral risk factors, such as smoking, while women are more susceptible to metabolic risk factors, such as a high BMI (Figure 5B).

## Discussion

Globally, the incidence (147.95%), prevalence (160.84%), and number of deaths (189.29%) due to dementia increased dramatically over the study period. Globally, the incidence is predicted to be 152.8 million in 2050 (GBD 2019 Dementia Forecasting Collaborators, 2022). Correspondingly, the global ASIR and ASPR (per 100,000 population) showed consistently increasing patterns over the 30-year study period. It is predicted that by 2050, the proportion of the population over the age of 60 years will increase by 22%, and they will experience the fastest population increase (Kanasi et al., 2016). Population growth and an aging population are causing an increase in the prevalence of the disease, and future medical costs for Alzheimer's disease and other dementias will continue to rise.

Dementia is an age-related disease, and its incidence has increased significantly worldwide with increasing life expectancy (Power et al., 2019). Our analysis supports this observation that the disease burden caused by dementia mainly affects elderly individuals. The incidence, prevalence, death, and DALYs of dementia increase with age. Moreover, the most significant increase in incidence, prevalence, death, and DALYs rates over time was observed in the 70+ age group. However, due to their shortened life expectancy, people over 90 years of age do not lose many life-years due to premature death and disability. During this period, the global DALY value and the ASR increased by 161.7 and 3.65%, respectively. The 2010 World Report on Alzheimer's Disease mentioned that the social cost of dementia was as high as those of tumors, heart disease, and stroke (Alzheimer's Disease International, 2010). According to the World Health Organization (WHO), dementia has become the seventh leading cause of death worldwide (Alzheimer's Association, 2022). Generally, the global burden of dementia has increased over time, and this trend will continue in the future with the aging population.

The ASIRs, ASPRs, ASDRs, and DALYs (per 100,000 population) were consistently higher in women than in men during this period among all the SDI regions. This finding suggests that the disease burden in women is higher than in men. The differences in the prevalence pattern between sexes may be due to reproductive capacity, hormone levels, genetic susceptibility, and mental status (Ngo et al., 2014). Women are more likely to develop structural and functional disorders of the nervous system (Ardekani et al., 2016), and they are two times as likely to suffer from psychological problems, such as depression, than men (Eid et al., 2019). All these conditions are risk factors for Alzheimer's disease. In addition, female brains are inherently more prone to Alzheimer's disease, which is influenced by sex hormones (Zhu et al., 2021). Moreover, women have a longer life expectancy than men (Tower, 2017), thus accounting for a considerable proportion of the aging population. Therefore, the prevention and treatment of dementia and interventions targeting female patients should be strengthened. Interestingly, the EAPCs in the ASIR, ASPR, ASDR, and DALYs (per 100,000 population) were larger in men than in women, suggesting that the disease burden in men is increasing at a faster rate than in women. Higher rates of smoking and drinking in men may explain this phenomenon (Wu et al., 2017).

Except for the ASIRs (per 100,000 population) in the low- and low-middle-SDI regions, the ASIRs, ASPRs, and DALYs (per 100,000 population) have gradually increased since 1995. In the low-SDI regions, the ASDR and ASR of DALYs (per 100,000 population) showed the largest increase. This phenomenon indicates that dementia is not an issue restricted to relatively low-income countries. In most areas, the disease burden of dementia remains significant. We found that the incidence, prevalence, and ASR of DALYs were positively related to the SDI, but the disease burden in young adults was negatively related to the SDI. This may be caused by the higher life expectancy in high-SDI regions and a greater degree of population aging over time (Kontis et al., 2017). However, high-SDI regions have better medical services and higher budgets for the care of people with dementia. Detecting dementia-related risk factors at an early stage greatly reduces the risk of death and the disease burden in people with dementia. In addition, high-SDI regions place greater emphasis on investing in educational attainment and social wellbeing, which can help improve physical, mental, and cognitive health and thus control the risk factors for the disease at an earlier stage (Gao and Liu, 2021). The decline in the ASIRs and ASPRs (per 100,000 population people) in some low-SDI regions may be related to the lower life expectancy of citizens in low-income countries (Chetty et al., 2016). Differences in the economic levels, policies, and cultures among countries may also be potential reasons. However, it is difficult to propose a uniform explanation for these results.

There has been no effective therapy to reverse dementia thus far, and drug treatment has substantial limitations (Gitlin et al., 2012). An effective strategy is to address the relative risk factors to prevent the development of the condition. Studies have shown that with increasing sustenance levels and quality of life, the incidence of metabolic diseases (high systolic blood pressure, high BMI, and diabetes) increases significantly (Charlot et al., 2021). The rapid development in the social economy has intensified the social competition and life pressure experienced by the people; this stress is often accompanied by harmful living habits (smoking, excessive alcohol consumption, and lack of physical activity). Our data showed that regions with higher SDIs were more affected by these risk factors than regions with low SDIs. In other words, high-income countries and regions have a higher risk of obesity, social stress, and lack of physical activity, and thus a higher risk of dementia. There has also been a decline in the incidence of dementia in some European countries. This may be due to effective interventions targeting cardiovascular, metabolic, cognitive, behavioral, and other factors related to dementia in recent years (Solomon et al., 2014). In 2019, the WHO formed a panel to reduce dementia risk, highlighting interventions that may help reduce the incidence of dementia (GBD 2019 Dementia Forecasting Collaborators, 2022). Relevant departments should improve medical services for dementia patients as soon as possible and raise public awareness. Early screening for risk factors and early diagnosis and treatment of associated diseases should be implemented. The government should integrate various resources and facilities in the region to promote improvement in the elderly care service system and the elderly healthcare system.

With the spread of COVID-19 in the last 3 years, some studies have found an increased risk of Alzheimer's disease in COVID-19 patients (Xia et al., 2021). Studies have shown that patients with severe SARS-CoV-2 infection exhibit cognitive decline and may eventually develop AD. This may be due to the viral involvement of the central nervous system directly, combined with the long-term accumulation of pro-inflammatory cytokines, which induces or accelerates the neurodegenerative process; patients are at a higher risk of subsequently developing AD (Heneka et al., 2013; Siu et al., 2019). Delirium is a common symptom of SARS-CoV-2 infection in patients with AD and is associated with a high short-term mortality rate (Poloni et al., 2020). Patients with AD are associated with increased permeability of the blood–brain barrier, which may facilitate the passage of SARS-CoV-2 across the blood–brain barrier (Mok et al., 2020). The virus may also enter the brainstem from the olfactory nerve, which may be responsible for the patient's respiratory failure (Netland et al., 2008). Once infected, patients with dementia are at increased risk of intracranial inflammation and increased mortality (Mok et al., 2020). However, it is unclear whether AD is a long-term complication of COVID-19, and further studies are needed to explain the contribution of COVID-19 to AD.

Conventional limitations have been reported in previous articles (Wang et al., 2021), and the limitations of this article are mainly as follows. To date, 12 risk factors connected with dementia have been reported (Livingston et al., 2020). However, GBD 2019 showed that only metabolic and behavioral factors were associated with dementia, but did not provide data on other risk factors. Second, dementia was not further classified by subtype; for example, dementia can be classified as vascular dementia or dementia with Lewy bodies. They differ in their disease burden and common risk factors, but the GBD 2019 currently has no data stratified by pathological subtype. In addition, the diagnostic criteria, biomarkers, medical records, and insurance codes for dementia globally have been updated over time, and there might be heterogeneity during the past three decades (GBD 2019 Diseases Injuries Collaborators, 2020; GBD 2019 Risk Factors Collaborators, 2020). The fallacies of the disease burden between our analysis and the real world are inevitable. Therefore, the results of our analysis need to be interpreted with caution, and further investigations are needed to perform.

## Conclusion

In conclusion, this study illustrates the global epidemiological trends of dementia from 1990 to 2019. Dementia has become a global public health problem as the population ages. The risk of developing dementia is proportional to age. The disease burden remains high in most countries and territories, while female and elderly individuals should be the focus of attention. More effective prevention and treatment measures are needed to reduce the disease burden caused by dementia.

## Data availability statement

The original contributions presented in the study are included in the article/Supplementary material, further inquiries can be directed to the corresponding author/s.

## Author contributions

XL and YL conceived and designed the study. XS and NH supervised the study. FH and XF performed the statistical analysis. XL drafted the manuscript. All authors contributed to the acquisition, analysis, interpretation of the data, revised the report, and approved the final version before submission.

## Funding

The present study was funded by the National Natural Science Foundation of China (Grant No. 82000755) and the Natural Science Foundation of Shandong Province (Grant No. ZR2020QH086).

## Conflict of interest

The authors declare that the research was conducted in the absence of any commercial or financial relationships that could be construed as a potential conflict of interest.

## Publisher's note

All claims expressed in this article are solely those of the authors and do not necessarily represent those of their affiliated organizations, or those of the publisher, the editors and the reviewers. Any product that may be evaluated in this article, or claim that may be made by its manufacturer, is not guaranteed or endorsed by the publisher.
