# Supplementary material for: Global, regional, and national burden of Alzheimer's disease and other dementias, 1990–2019
Source: Front Aging Neurosci. 2022 Oct 10;14:937486. doi: 10.3389/fnagi.2022.937486 (PMC9588915; doi:10.3389/fnagi.2022.937486)
Supplement: Supplementary file 1 [file Data_Sheet_1.docx]

Supplementary Material


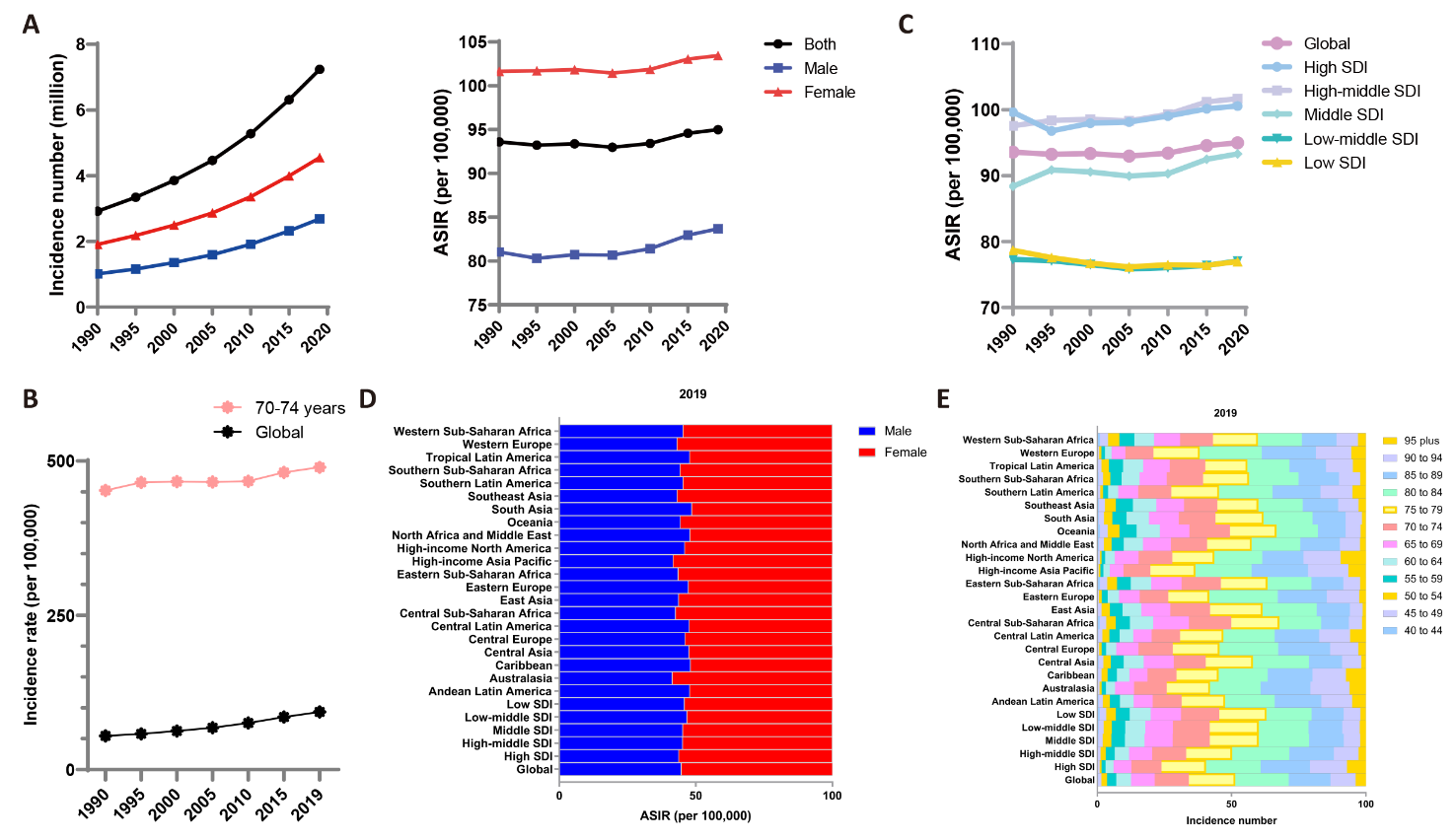


**Supplement Figure 1** Sex and age distribution of ASIR in Alzheimer's disease and other dementias. **(A)** Trends of gender incidence cases and ASIR in Alzheimer's disease and other dementias from 1990 to 2019; (**B**) Trends in incidence rates in the 70-74 age group from 1990 to 2019; (**C)** Trend of ASIR in each SDI region; (**D)** Gender distribution of ASIR by SDI region in 2019; (**E)** Age distribution of incidence number by SDI region in 2019. SDI, sociodemographic index; ASIR, age-standardized incidence rate.


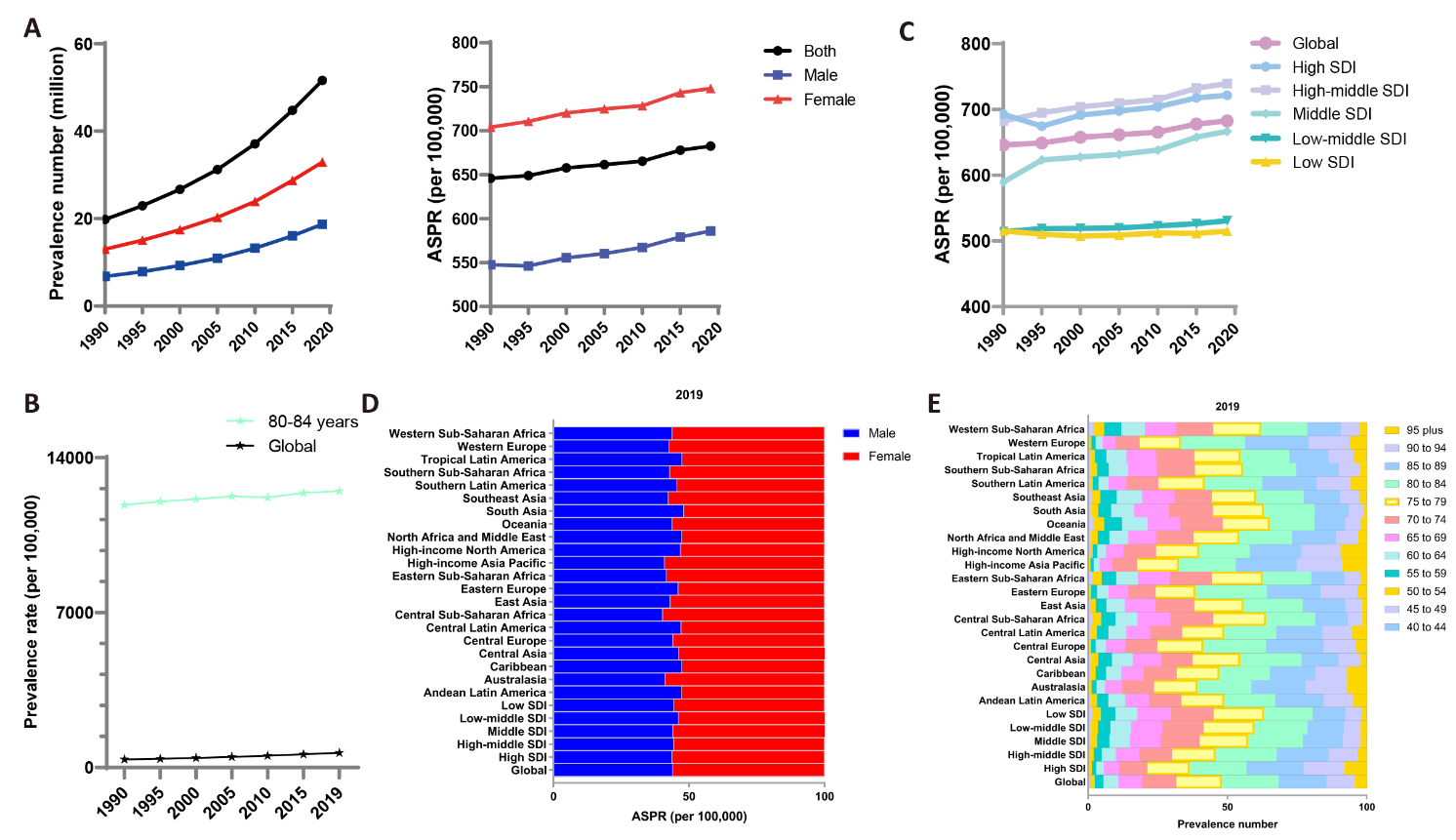


**Supplement Figure 2** Sex and age distribution of ASPR in Alzheimer's disease and other dementias. **(A)** Trends of gender prevalence cases and ASPR in Alzheimer's disease and other dementias from 1990 to 2019; **(B)** Trends in prevalence rates in the 80-84 age group from 1990 to 2019; **(C)** Trend of ASPR in each SDI region; **(D)** Gender distribution of ASPR by SDI area in 2019; **(E)** Age distribution of prevalence number by SDI region in 2019. SDI, sociodemographic index; ASPR, age-standardized prevalence rate.


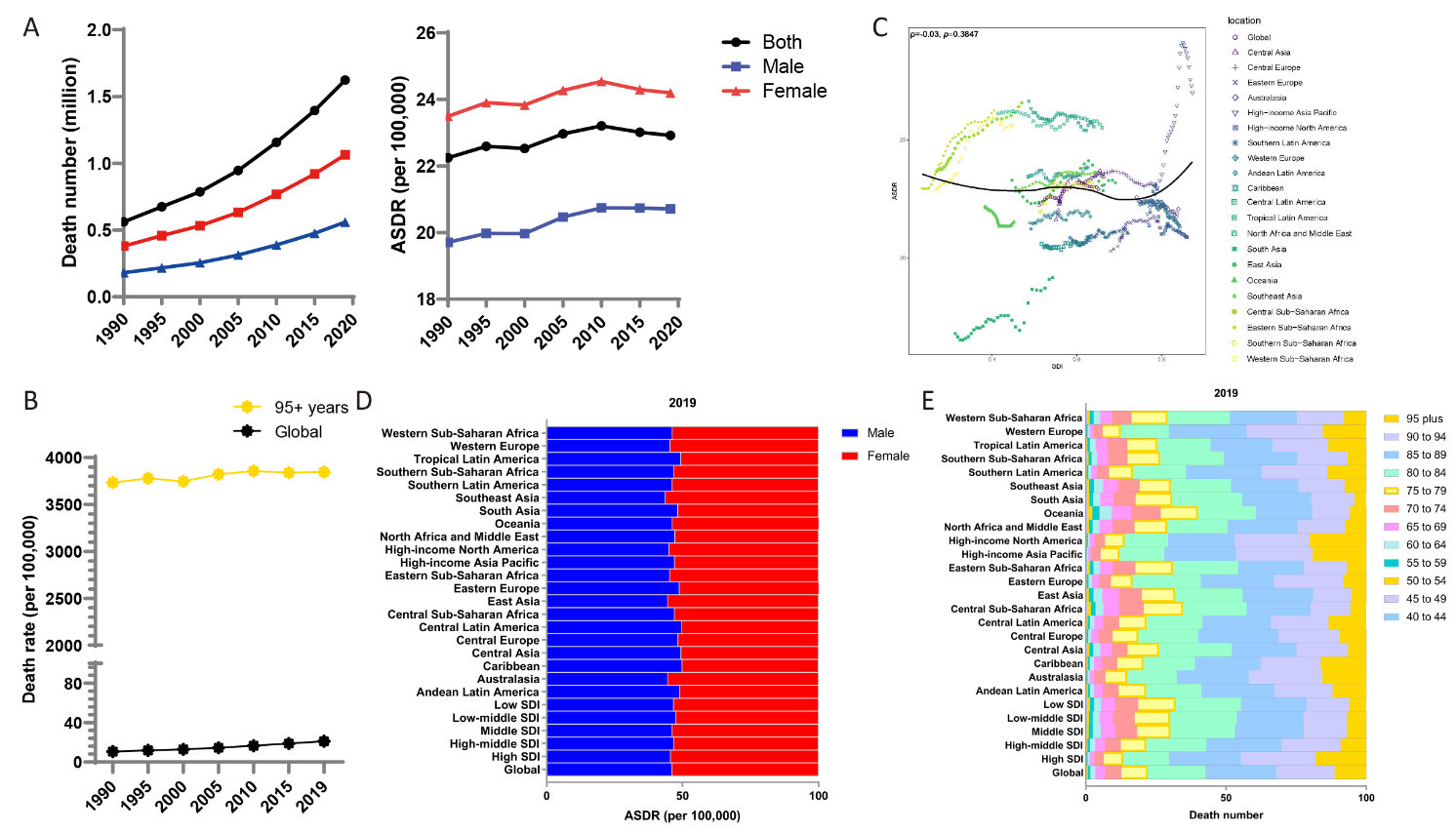


**Supplement Figure 3** Sex and age distribution of ASDR in Alzheimer's disease and other dementias. **(A)** Trends of gender death cases and ASDR in Alzheimer's disease and other dementias from 1990 to 2019; **(B)** Trends in death rates in the 95+ age group from 1990 to 2019; **(C)** The correlation between ASDR of Alzheimer's disease and other dementias and SDI regions in 2019; The association was calculated with Pearson correlation analysis; **(D)** Gender distribution of ASDR by SDI area in 2019; **(E)** Age distribution of death number by SDI region in 2019. SDI, sociodemographic index; ASDR, age-standardized deaths rate.


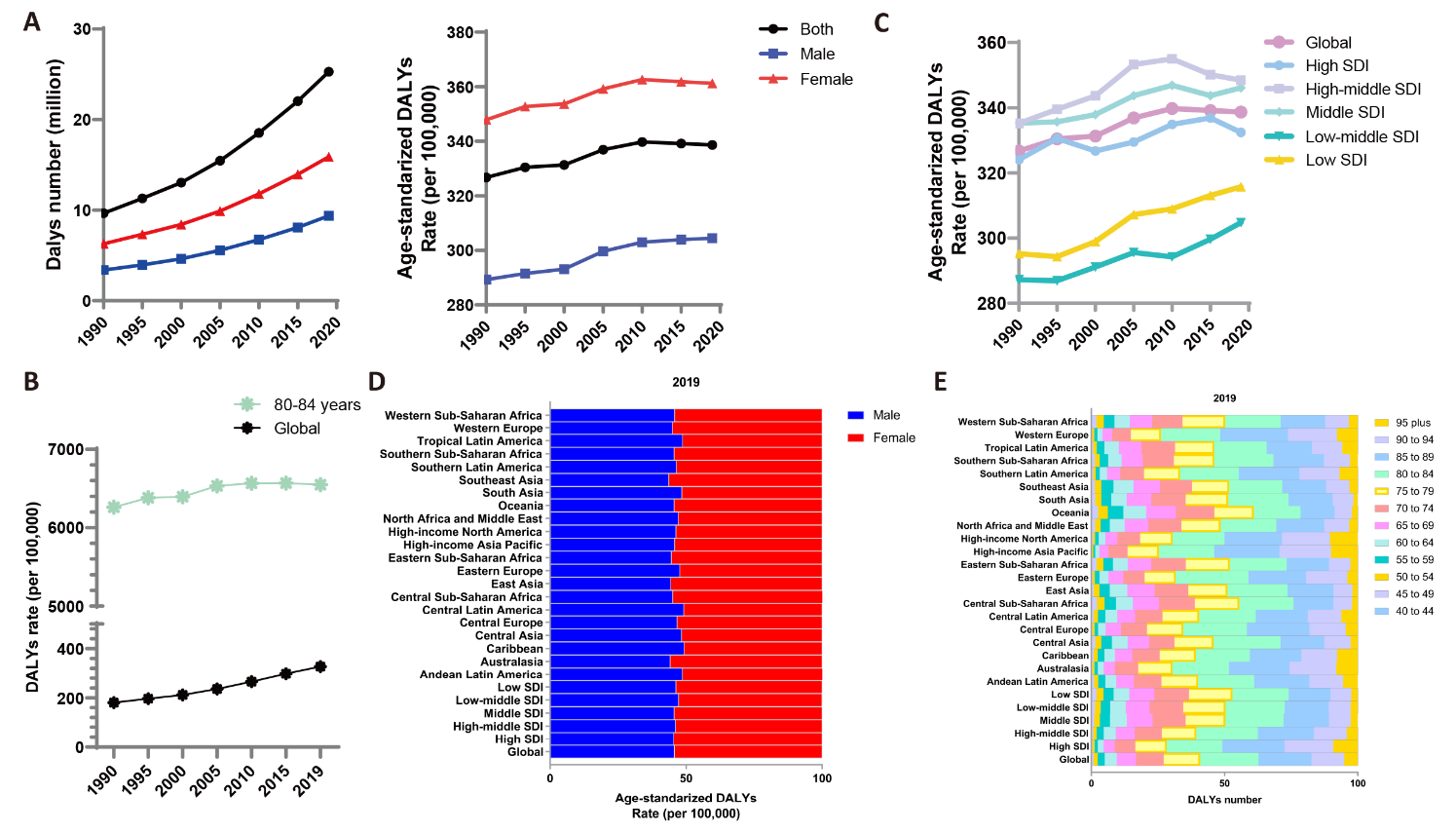


**Supplement Figure 4** Sex and age distribution of ASR of DALYs in Alzheimer's disease and other dementias. **(A)** Trends of gender DALYs and ASR of DALYs in Alzheimer's disease and other dementias from 1990 to 2019; **(B)** Trends in DALYs rates in the 80-84 age group from 1990 to 2019; **(C)** Trend of ASR of DALYs in each SDI region; **(D)** Gender distribution of ASR of DALYs by SDI area in 2019; **(E)** Age distribution of DALYs number by SDI region in 2019. DALYs, disability-adjusted life years; SDI, sociodemographic index; ASR, age-standardized rate.


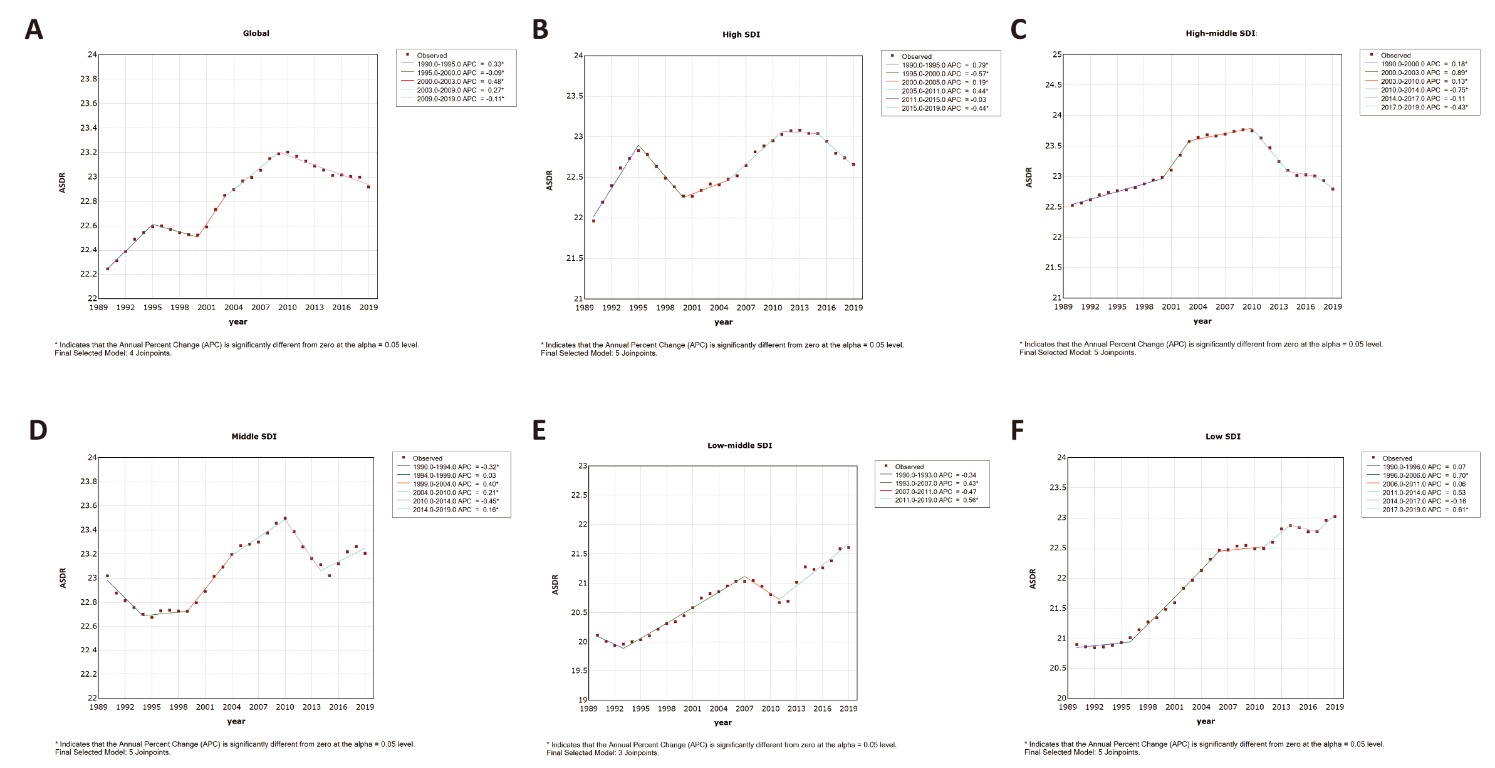


**Supplement Figure 5.** Joinpoint regression analysis of the ASDR for Alzheimer’s disease and other dementias in global and SDI regions from 1990 to 2019. SDI, sociodemographic index; ASDR, age-standardized deaths rate.

**Supplement Table 1.** Incidence cases and age-standardized incidence rates (ASIR) of Alzheimer's disease and other dementias in 1990 and 2019 and the temporal trends from 1990 to 2019 in 204 countries.

|  | **1990** | | **2019** | | **EAPC of ASIR from 1990 to 2019** |
| --- | --- | --- | --- | --- | --- |
| **location** | **Incidence cases (*10^3)** | **ASIR (*10^3)** | **Incidence cases (*10^3)** | **ASIR (*10^3)** |  |
| Afghanistan | 5.45 (4.59-6.31) | 0.11 (0.09-0.13) | 8.46 (7.1-9.79) | 0.11 (0.09-0.13) | -0.09 (-0.14 to -0.05) |
| Albania | 1.73 (1.47-1.99) | 0.11 (0.09-0.12) | 4.5 (3.77-5.22) | 0.11 (0.09-0.12) | 0.05 (0.03 to 0.06) |
| Algeria | 8.74 (7.23-10.4) | 0.11 (0.09-0.13) | 28.17 (23.65-32.8) | 0.11 (0.09-0.13) | -0.01 (-0.04 to 0.01) |
| American Samoa | 0.01 (0.01-0.01) | 0.09 (0.07-0.1) | 0.03 (0.03-0.04) | 0.09 (0.07-0.1) | 0 (0 to 0.01) |
| Andorra | 0.04 (0.03-0.04) | 0.09 (0.08-0.1) | 0.13 (0.12-0.15) | 0.09 (0.07-0.1) | -0.03 (-0.04 to -0.01) |
| Angola | 2.09 (1.79-2.42) | 0.1 (0.08-0.11) | 6.4 (5.52-7.36) | 0.1 (0.08-0.11) | 0.03 (0 to 0.06) |
| Antigua | 0.05 (0.04-0.05) | 0.08 (0.07-0.09) | 0.07 (0.06-0.08) | 0.08 (0.07-0.09) | 0.05 (0.03 to 0.07) |
| Argentina | 26.46 (22.08-31.03) | 0.09 (0.08-0.11) | 53.88 (45.64-61.93) | 0.1 (0.08-0.11) | 0.08 (0.07 to 0.09) |
| Armenia | 2.03 (1.71-2.36) | 0.1 (0.09-0.12) | 4.12 (3.47-4.78) | 0.1 (0.09-0.12) | 0.14 (0.12 to 0.17) |
| Australia | 17.72 (15.11-20.28) | 0.1 (0.08-0.11) | 43.97 (37.68-49.87) | 0.09 (0.08-0.11) | -0.06 (-0.08 to -0.04) |
| Austria | 11.6 (9.61-13.66) | 0.09 (0.08-0.11) | 18.83 (15.9-21.54) | 0.09 (0.07-0.1) | -0.13 (-0.15 to -0.11) |
| Azerbaijan | 4.27 (3.65-4.86) | 0.1 (0.09-0.12) | 5.91 (4.96-6.94) | 0.1 (0.09-0.12) | 0.02 (-0.01 to 0.06) |
| Bahamas | 0.1 (0.08-0.11) | 0.08 (0.07-0.09) | 0.26 (0.22-0.29) | 0.08 (0.07-0.09) | 0.03 (0.01 to 0.05) |
| Bahrain | 0.1 (0.09-0.12) | 0.11 (0.09-0.13) | 0.57 (0.49-0.66) | 0.11 (0.1-0.13) | 0.07 (0.06 to 0.08) |
| Bangladesh | 24.46 (20.88-28.27) | 0.07 (0.06-0.08) | 76.67 (65.45-88.99) | 0.07 (0.06-0.08) | -0.01 (-0.04 to 0.03) |
| Barbados | 0.25 (0.2-0.29) | 0.08 (0.07-0.09) | 0.39 (0.34-0.45) | 0.08 (0.07-0.09) | 0.06 (0.04 to 0.07) |
| Belarus | 12.23 (10.19-14.2) | 0.1 (0.09-0.12) | 17.25 (14.46-19.85) | 0.1 (0.09-0.12) | 0.1 (0.06 to 0.13) |
| Belgium | 15.32 (12.89-17.94) | 0.1 (0.08-0.11) | 24.63 (20.68-28.28) | 0.09 (0.08-0.1) | -0.28 (-0.3 to -0.25) |
| Belize | 0.07 (0.06-0.08) | 0.08 (0.07-0.09) | 0.18 (0.16-0.21) | 0.08 (0.07-0.09) | 0.01 (-0.02 to 0.04) |
| Benin | 1.17 (1-1.34) | 0.08 (0.06-0.09) | 2.49 (2.14-2.86) | 0.07 (0.06-0.08) | -0.02 (-0.07 to 0.03) |
| Bermuda | 0.04 (0.04-0.05) | 0.08 (0.07-0.09) | 0.12 (0.1-0.13) | 0.08 (0.07-0.09) | 0.09 (0.08 to 0.09) |
| Bhutan | 0.1 (0.09-0.12) | 0.07 (0.06-0.08) | 0.31 (0.27-0.36) | 0.07 (0.06-0.08) | 0.01 (-0.03 to 0.04) |
| Bolivia | 1.86 (1.59-2.17) | 0.08 (0.07-0.09) | 5.81 (4.95-6.66) | 0.08 (0.07-0.1) | 0.08 (0.04 to 0.11) |
| Bosnia and Herzegovina | 3 (2.52-3.51) | 0.11 (0.09-0.12) | 5.85 (4.82-6.83) | 0.1 (0.09-0.12) | -0.03 (-0.05 to -0.01) |
| Botswana | 0.33 (0.28-0.38) | 0.09 (0.08-0.1) | 0.78 (0.67-0.89) | 0.09 (0.08-0.1) | 0 (-0.03 to 0.02) |
| Brazil | 67.67 (57.95-77.56) | 0.1 (0.09-0.12) | 231.85 (201.82-262.88) | 0.1 (0.09-0.12) | 0.09 (0.07 to 0.1) |
| Brunei | 0.05 (0.04-0.06) | 0.1 (0.08-0.11) | 0.15 (0.13-0.18) | 0.1 (0.08-0.11) | -0.03 (-0.11 to 0.04) |
| Bulgaria | 9.9 (8.05-11.84) | 0.1 (0.08-0.12) | 16.11 (13.34-18.76) | 0.1 (0.09-0.12) | 0.09 (0.07 to 0.1) |
| Burkina Faso | 2.18 (1.86-2.54) | 0.08 (0.07-0.09) | 4.83 (4.19-5.51) | 0.08 (0.07-0.09) | 0 (-0.03 to 0.03) |
| Burundi | 1.4 (1.19-1.63) | 0.09 (0.07-0.1) | 2.36 (2.05-2.68) | 0.08 (0.07-0.1) | -0.08 (-0.1 to -0.06) |
| Cape Verde | 0.19 (0.16-0.22) | 0.08 (0.07-0.09) | 0.32 (0.28-0.37) | 0.08 (0.07-0.09) | 0.07 (0.05 to 0.08) |
| Cambodia | 2.55 (2.17-2.94) | 0.09 (0.07-0.1) | 7.54 (6.45-8.61) | 0.09 (0.07-0.1) | 0 (-0.04 to 0.04) |
| Cameroon | 2.13 (1.82-2.45) | 0.07 (0.06-0.09) | 5.76 (4.96-6.6) | 0.08 (0.06-0.09) | 0.06 (0.03 to 0.09) |
| Canada | 32.83 (27.82-37.93) | 0.1 (0.09-0.12) | 74.73 (63.65-85.04) | 0.1 (0.08-0.11) | -0.26 (-0.34 to -0.19) |
| Central African Republic | 0.64 (0.55-0.75) | 0.1 (0.09-0.12) | 1.18 (1.02-1.35) | 0.1 (0.09-0.12) | 0.02 (-0.01 to 0.04) |
| Chad | 1.63 (1.39-1.88) | 0.08 (0.07-0.09) | 2.85 (2.46-3.27) | 0.08 (0.06-0.09) | -0.08 (-0.11 to -0.05) |
| Chile | 7.19 (6.01-8.41) | 0.09 (0.07-0.1) | 22.45 (18.97-25.74) | 0.09 (0.08-0.11) | 0.23 (0.19 to 0.27) |
| China | 512.83 (435.06-596.01) | 0.09 (0.08-0.1) | 1800.33 (1517.95-2081.95) | 0.1 (0.09-0.12) | 0.33 (0.27 to 0.4) |
| Colombia | 11.43 (9.82-13.17) | 0.09 (0.07-0.1) | 48.67 (41.7-55.44) | 0.09 (0.07-0.1) | 0.09 (0.07 to 0.11) |
| Comoros | 0.14 (0.12-0.16) | 0.09 (0.07-0.1) | 0.33 (0.29-0.38) | 0.08 (0.07-0.1) | 0 (-0.03 to 0.02) |
| Republic of Congo | 0.62 (0.52-0.73) | 0.1 (0.08-0.11) | 1.63 (1.45-1.82) | 0.1 (0.09-0.11) | 0.17 (0.11 to 0.24) |
| Cook Islands | 0.01 (0.01-0.01) | 0.09 (0.07-0.1) | 0.02 (0.02-0.02) | 0.09 (0.07-0.1) | -0.01 (-0.02 to 0.01) |
| Costa Rica | 1.31 (1.11-1.52) | 0.08 (0.07-0.1) | 4.42 (3.8-5.04) | 0.09 (0.07-0.1) | 0.06 (0.05 to 0.07) |
| Croatia | 5.71 (4.71-6.73) | 0.1 (0.09-0.12) | 10.34 (8.64-12) | 0.11 (0.09-0.12) | 0.08 (0.07 to 0.1) |
| Cuba | 7.27 (6.15-8.48) | 0.08 (0.07-0.09) | 15.96 (13.72-18.17) | 0.08 (0.07-0.09) | 0.03 (0.01 to 0.05) |
| Cyprus | 0.59 (0.48-0.71) | 0.09 (0.07-0.1) | 1.64 (1.37-1.93) | 0.09 (0.08-0.1) | 0.09 (0.07 to 0.1) |
| Czech Republic | 13.33 (10.95-15.67) | 0.1 (0.09-0.12) | 24.31 (20.63-27.91) | 0.11 (0.09-0.12) | 0.18 (0.17 to 0.19) |
| Ivory Coast | 1.59 (1.36-1.82) | 0.07 (0.06-0.09) | 4.84 (4.17-5.53) | 0.08 (0.06-0.09) | 0.03 (0 to 0.07) |
| North Korea | 9.98 (8.51-11.51) | 0.09 (0.08-0.11) | 25.31 (21.17-29.59) | 0.09 (0.08-0.1) | -0.14 (-0.18 to -0.1) |
| Democratic Republic of the Congo | 8.59 (7.3-10.02) | 0.1 (0.08-0.11) | 22.35 (19.29-25.51) | 0.1 (0.08-0.11) | 0.09 (0.08 to 0.1) |
| Denmark | 8.12 (6.87-9.43) | 0.09 (0.08-0.1) | 11.01 (9.4-12.68) | 0.08 (0.07-0.1) | -0.27 (-0.3 to -0.24) |
| Djibouti | 0.06 (0.05-0.07) | 0.09 (0.07-0.1) | 0.28 (0.25-0.32) | 0.08 (0.07-0.1) | -0.09 (-0.12 to -0.06) |
| Dominica | 0.06 (0.05-0.07) | 0.08 (0.07-0.09) | 0.07 (0.06-0.08) | 0.08 (0.07-0.09) | -0.02 (-0.05 to 0) |
| Dominican Republic | 2.26 (1.92-2.63) | 0.08 (0.07-0.09) | 6.53 (5.57-7.49) | 0.08 (0.07-0.09) | -0.01 (-0.04 to 0.02) |
| Ecuador | 3.54 (2.99-4.1) | 0.08 (0.07-0.09) | 11.21 (9.54-12.91) | 0.08 (0.07-0.1) | 0.09 (0.07 to 0.11) |
| Egypt | 20.23 (17.17-23.3) | 0.1 (0.09-0.12) | 44.04 (37.5-50.79) | 0.11 (0.09-0.12) | 0.16 (0.12 to 0.2) |
| El Salvador | 2.3 (1.95-2.65) | 0.09 (0.07-0.1) | 5.67 (4.86-6.5) | 0.09 (0.07-0.1) | 0.02 (0 to 0.05) |
| Equatorial Guinea | 0.12 (0.1-0.14) | 0.1 (0.08-0.11) | 0.32 (0.27-0.37) | 0.1 (0.08-0.11) | 0.06 (0.04 to 0.08) |
| Eritrea | 0.43 (0.37-0.5) | 0.09 (0.08-0.1) | 1.28 (1.1-1.47) | 0.09 (0.08-0.1) | -0.1 (-0.13 to -0.07) |
| Estonia | 1.89 (1.55-2.23) | 0.1 (0.08-0.11) | 3.33 (2.8-3.86) | 0.11 (0.09-0.12) | 0.28 (0.26 to 0.31) |
| Swaziland | 0.17 (0.15-0.2) | 0.09 (0.08-0.1) | 0.33 (0.28-0.38) | 0.09 (0.08-0.1) | -0.01 (-0.06 to 0.04) |
| Ethiopia | 9.42 (8.06-10.93) | 0.09 (0.08-0.1) | 25.37 (21.91-29.05) | 0.09 (0.07-0.1) | -0.07 (-0.12 to -0.01) |
| Fiji | 0.18 (0.16-0.21) | 0.09 (0.07-0.1) | 0.41 (0.35-0.48) | 0.09 (0.07-0.1) | -0.01 (-0.03 to 0.01) |
| Finland | 6.76 (5.67-7.95) | 0.09 (0.08-0.11) | 12.77 (10.79-14.68) | 0.09 (0.07-0.1) | -0.24 (-0.26 to -0.21) |
| France | 77.17 (65.56-89.3) | 0.09 (0.07-0.1) | 153.52 (129.67-175.43) | 0.09 (0.08-0.1) | 0.22 (0.15 to 0.29) |
| Gabon | 0.4 (0.34-0.46) | 0.1 (0.08-0.11) | 0.73 (0.63-0.84) | 0.1 (0.08-0.11) | 0.02 (0 to 0.04) |
| Gambia | 0.17 (0.14-0.19) | 0.08 (0.06-0.09) | 0.54 (0.46-0.63) | 0.08 (0.06-0.09) | 0 (-0.03 to 0.03) |
| Georgia | 5.07 (4.27-5.94) | 0.1 (0.09-0.12) | 7.04 (6.01-8.03) | 0.1 (0.09-0.12) | 0.01 (0 to 0.03) |
| Germany | 132.5 (117.17-148.73) | 0.1 (0.09-0.11) | 215.09 (182.74-249.24) | 0.09 (0.08-0.11) | -0.14 (-0.19 to -0.09) |
| Ghana | 2.8 (2.4-3.21) | 0.07 (0.06-0.08) | 8.03 (6.9-9.2) | 0.08 (0.06-0.09) | 0.06 (0.03 to 0.09) |
| Greece | 13.25 (11.03-15.49) | 0.09 (0.08-0.1) | 26.81 (22.41-31.19) | 0.09 (0.08-0.1) | -0.04 (-0.06 to -0.02) |
| Greenland | 0.02 (0.02-0.02) | 0.1 (0.09-0.12) | 0.05 (0.04-0.06) | 0.1 (0.09-0.12) | 0 (-0.04 to 0.04) |
| Grenada | 0.07 (0.06-0.08) | 0.08 (0.07-0.09) | 0.07 (0.06-0.08) | 0.08 (0.07-0.09) | 0.04 (0.03 to 0.05) |
| Guam | 0.04 (0.03-0.05) | 0.09 (0.07-0.1) | 0.16 (0.13-0.18) | 0.09 (0.07-0.1) | -0.03 (-0.04 to -0.01) |
| Guatemala | 2 (1.7-2.31) | 0.09 (0.07-0.1) | 8.19 (6.92-9.61) | 0.09 (0.07-0.1) | 0.04 (0.02 to 0.06) |
| Guinea | 1.85 (1.58-2.13) | 0.08 (0.06-0.09) | 3.19 (2.73-3.65) | 0.08 (0.06-0.09) | -0.01 (-0.03 to 0.01) |
| Guinea-Bissau | 0.18 (0.16-0.21) | 0.08 (0.06-0.09) | 0.33 (0.28-0.38) | 0.08 (0.07-0.09) | 0.04 (0 to 0.07) |
| Guyana | 0.21 (0.18-0.24) | 0.08 (0.07-0.09) | 0.36 (0.31-0.41) | 0.08 (0.07-0.09) | 0.05 (0.02 to 0.08) |
| Haiti | 1.59 (1.35-1.86) | 0.08 (0.07-0.09) | 3.65 (3.11-4.23) | 0.08 (0.07-0.09) | -0.03 (-0.08 to 0.02) |
| Honduras | 1.36 (1.17-1.58) | 0.08 (0.07-0.1) | 3.99 (3.4-4.64) | 0.09 (0.07-0.1) | -0.01 (-0.04 to 0.03) |
| Hungary | 13.69 (11.21-16.15) | 0.1 (0.09-0.12) | 22.72 (18.99-26.25) | 0.11 (0.09-0.12) | 0.19 (0.18 to 0.2) |
| Iceland | 0.28 (0.23-0.32) | 0.09 (0.08-0.1) | 0.56 (0.47-0.64) | 0.09 (0.07-0.1) | -0.07 (-0.08 to -0.06) |
| India | 158.97 (135.78-184.03) | 0.06 (0.05-0.07) | 541.46 (464.09-623.6) | 0.06 (0.05-0.07) | 0.03 (-0.02 to 0.08) |
| Indonesia | 54.02 (46.55-61.62) | 0.08 (0.07-0.1) | 123.57 (105.72-141.12) | 0.08 (0.07-0.09) | -0.05 (-0.1 to 0) |
| Iran | 17.34 (14.9-19.96) | 0.11 (0.09-0.13) | 67.89 (57.89-77.45) | 0.11 (0.09-0.13) | 0 (-0.03 to 0.02) |
| Iraq | 6.59 (5.57-7.63) | 0.11 (0.09-0.12) | 17.48 (14.95-20) | 0.11 (0.09-0.12) | 0.08 (0.05 to 0.11) |
| Ireland | 3.54 (2.93-4.22) | 0.09 (0.08-0.1) | 6.94 (5.88-7.98) | 0.09 (0.07-0.1) | -0.05 (-0.07 to -0.04) |
| Israel | 4.08 (3.36-4.88) | 0.09 (0.07-0.1) | 11.17 (9.51-12.84) | 0.09 (0.08-0.1) | 0.01 (-0.01 to 0.03) |
| Italy | 90.75 (74.77-106.63) | 0.1 (0.08-0.12) | 186.11 (156.69-214.29) | 0.1 (0.09-0.12) | 0.06 (0.02 to 0.09) |
| Jamaica | 1.45 (1.22-1.68) | 0.08 (0.07-0.09) | 2.65 (2.26-3.03) | 0.08 (0.07-0.09) | 0.03 (0.01 to 0.05) |
| Japan | 146.81 (124.5-170.76) | 0.1 (0.08-0.11) | 538.79 (460.91-613.07) | 0.11 (0.1-0.13) | 0.58 (0.53 to 0.62) |
| Jordan | 0.9 (0.76-1.03) | 0.11 (0.09-0.12) | 4.71 (4.02-5.45) | 0.11 (0.09-0.12) | 0.07 (0.05 to 0.1) |
| Kazakhstan | 9.99 (8.38-11.63) | 0.1 (0.08-0.12) | 12.98 (10.82-15.17) | 0.1 (0.09-0.12) | 0.06 (0.03 to 0.1) |
| Kenya | 4.85 (4.18-5.6) | 0.09 (0.07-0.1) | 12.16 (10.56-13.84) | 0.09 (0.07-0.1) | 0.03 (0 to 0.05) |
| Kiribati | 0.02 (0.02-0.02) | 0.09 (0.07-0.1) | 0.03 (0.03-0.04) | 0.09 (0.07-0.1) | -0.06 (-0.09 to -0.03) |
| Kuwait | 0.42 (0.36-0.47) | 0.11 (0.09-0.13) | 2.05 (1.8-2.32) | 0.11 (0.1-0.13) | 0.03 (0.01 to 0.05) |
| Kyrgyzstan | 2.79 (2.35-3.23) | 0.1 (0.09-0.12) | 3.59 (3.08-4.1) | 0.1 (0.09-0.12) | 0.14 (0.1 to 0.17) |
| Laos | 1.12 (0.95-1.31) | 0.09 (0.07-0.1) | 2.57 (2.21-2.93) | 0.08 (0.07-0.1) | -0.06 (-0.12 to -0.01) |
| Latvia | 3.38 (2.77-3.97) | 0.1 (0.08-0.11) | 4.84 (4.02-5.63) | 0.1 (0.09-0.12) | 0.15 (0.13 to 0.17) |
| Lebanon | 1.83 (1.55-2.1) | 0.11 (0.09-0.12) | 5.62 (4.79-6.49) | 0.11 (0.1-0.13) | 0.05 (0.04 to 0.07) |
| Lesotho | 0.64 (0.55-0.74) | 0.09 (0.08-0.1) | 0.73 (0.62-0.84) | 0.09 (0.08-0.1) | -0.07 (-0.12 to -0.03) |
| Liberia | 0.57 (0.49-0.67) | 0.07 (0.06-0.09) | 1.06 (0.92-1.2) | 0.08 (0.06-0.09) | 0.05 (0.03 to 0.08) |
| Libya | 1.64 (1.41-1.89) | 0.11 (0.09-0.13) | 4.41 (3.77-5.01) | 0.11 (0.09-0.12) | -0.08 (-0.11 to -0.06) |
| Lithuania | 4.49 (3.75-5.21) | 0.1 (0.09-0.12) | 7.08 (5.87-8.23) | 0.1 (0.09-0.12) | 0.07 (0.04 to 0.1) |
| Luxembourg | 0.49 (0.41-0.58) | 0.09 (0.08-0.1) | 0.9 (0.76-1.03) | 0.08 (0.07-0.09) | -0.42 (-0.52 to -0.33) |
| Madagascar | 2.89 (2.49-3.34) | 0.08 (0.07-0.1) | 5.24 (4.54-5.98) | 0.08 (0.07-0.09) | 0.03 (0.01 to 0.06) |
| Malawi | 2.03 (1.74-2.36) | 0.09 (0.07-0.1) | 4.32 (3.75-4.94) | 0.09 (0.07-0.1) | 0.03 (0.01 to 0.05) |
| Malaysia | 5.93 (5.07-6.81) | 0.08 (0.07-0.1) | 17.86 (15.44-20.38) | 0.08 (0.07-0.1) | 0.01 (-0.01 to 0.03) |
| Maldives | 0.04 (0.04-0.05) | 0.08 (0.07-0.09) | 0.21 (0.18-0.23) | 0.09 (0.07-0.1) | 0.18 (0.15 to 0.2) |
| Mali | 2 (1.71-2.3) | 0.08 (0.07-0.09) | 4.56 (3.94-5.23) | 0.08 (0.07-0.09) | -0.01 (-0.04 to 0.02) |
| Malta | 0.35 (0.29-0.41) | 0.09 (0.08-0.1) | 0.92 (0.78-1.06) | 0.09 (0.08-0.1) | -0.04 (-0.06 to -0.02) |
| Marshall Islands | 0.01 (0.01-0.01) | 0.09 (0.07-0.1) | 0.01 (0.01-0.02) | 0.08 (0.07-0.1) | -0.15 (-0.2 to -0.11) |
| Mauritania | 0.53 (0.45-0.61) | 0.07 (0.06-0.09) | 1.21 (1.03-1.38) | 0.07 (0.06-0.09) | 0.02 (0 to 0.05) |
| Mauritius | 0.47 (0.4-0.54) | 0.09 (0.07-0.1) | 1.34 (1.15-1.51) | 0.09 (0.07-0.1) | 0 (-0.02 to 0.02) |
| Mexico | 27.33 (23.13-31.8) | 0.08 (0.07-0.1) | 82.91 (70.48-95.27) | 0.08 (0.07-0.09) | -0.05 (-0.11 to 0.01) |
| Micronesia | 0.02 (0.02-0.03) | 0.09 (0.07-0.1) | 0.03 (0.03-0.04) | 0.09 (0.07-0.1) | 0 (-0.02 to 0.03) |
| Monaco | 0.08 (0.06-0.09) | 0.09 (0.08-0.1) | 0.1 (0.09-0.12) | 0.09 (0.07-0.1) | -0.11 (-0.13 to -0.09) |
| Mongolia | 0.78 (0.66-0.91) | 0.1 (0.09-0.12) | 1.42 (1.21-1.65) | 0.1 (0.09-0.12) | 0.07 (0.03 to 0.1) |
| Montenegro | 0.58 (0.48-0.67) | 0.11 (0.09-0.12) | 0.98 (0.82-1.14) | 0.11 (0.09-0.12) | 0.05 (0.04 to 0.06) |
| Morocco | 10.65 (9.01-12.33) | 0.11 (0.09-0.13) | 25.59 (21.79-29.49) | 0.11 (0.09-0.12) | -0.05 (-0.08 to -0.02) |
| Mozambique | 3.22 (2.76-3.72) | 0.09 (0.07-0.1) | 6.1 (5.27-6.97) | 0.09 (0.07-0.1) | -0.01 (-0.04 to 0.01) |
| Myanmar | 13.38 (11.41-15.39) | 0.09 (0.07-0.1) | 30.74 (26.37-35.37) | 0.09 (0.07-0.1) | -0.02 (-0.06 to 0.03) |
| Namibia | 0.44 (0.37-0.52) | 0.09 (0.07-0.1) | 0.97 (0.83-1.11) | 0.09 (0.08-0.1) | 0.07 (0.03 to 0.12) |
| Nauru | 0 (0-0) | 0.09 (0.07-0.1) | 0 (0-0) | 0.08 (0.07-0.1) | -0.08 (-0.12 to -0.05) |
| Nepal | 4.08 (3.49-4.69) | 0.07 (0.06-0.08) | 11.49 (9.91-13.19) | 0.07 (0.06-0.08) | -0.02 (-0.06 to 0.02) |
| Netherlands | 19.73 (17.35-21.97) | 0.09 (0.08-0.1) | 35.16 (30.12-40.11) | 0.09 (0.08-0.11) | 0.01 (-0.02 to 0.03) |
| New Zealand | 3.7 (3.13-4.28) | 0.1 (0.08-0.11) | 8.46 (7.2-9.67) | 0.1 (0.08-0.11) | -0.02 (-0.03 to -0.01) |
| Nicaragua | 1.04 (0.89-1.19) | 0.09 (0.07-0.1) | 3 (2.57-3.46) | 0.09 (0.08-0.1) | 0.04 (0.02 to 0.07) |
| Niger | 1.24 (1.06-1.43) | 0.08 (0.07-0.09) | 3.73 (3.22-4.27) | 0.08 (0.07-0.09) | 0.02 (-0.02 to 0.06) |
| Nigeria | 22.65 (19.45-26.05) | 0.07 (0.06-0.09) | 41.78 (36.08-47.57) | 0.07 (0.06-0.08) | -0.27 (-0.38 to -0.15) |
| Niue | 0 (0-0) | 0.09 (0.08-0.1) | 0 (0-0) | 0.09 (0.07-0.1) | -0.06 (-0.09 to -0.03) |
| North Macedonia | 1.44 (1.2-1.68) | 0.1 (0.08-0.12) | 2.74 (2.26-3.21) | 0.11 (0.09-0.12) | 0.14 (0.13 to 0.16) |
| Northern Mariana Islands | 0.01 (0.01-0.01) | 0.09 (0.07-0.1) | 0.03 (0.02-0.03) | 0.09 (0.07-0.1) | 0.06 (0.05 to 0.07) |
| Norway | 7.34 (6.23-8.53) | 0.1 (0.08-0.11) | 9.91 (8.46-11.3) | 0.09 (0.08-0.1) | -0.18 (-0.22 to -0.15) |
| Oman | 0.42 (0.36-0.48) | 0.11 (0.09-0.13) | 1.02 (0.87-1.17) | 0.11 (0.09-0.13) | -0.03 (-0.04 to -0.02) |
| Pakistan | 30.33 (25.84-35.17) | 0.07 (0.06-0.08) | 48.73 (41.9-55.79) | 0.07 (0.06-0.08) | -0.02 (-0.07 to 0.04) |
| Palau | 0.01 (0-0.01) | 0.09 (0.07-0.1) | 0.01 (0.01-0.01) | 0.09 (0.07-0.1) | -0.01 (-0.04 to 0.01) |
| Palestine | 0.75 (0.64-0.85) | 0.11 (0.09-0.12) | 1.77 (1.51-2.02) | 0.11 (0.09-0.12) | 0.02 (0 to 0.04) |
| Panama | 1.13 (0.96-1.3) | 0.08 (0.07-0.1) | 3.68 (3.15-4.23) | 0.09 (0.07-0.1) | 0.05 (0.04 to 0.07) |
| Papua New Guinea | 0.9 (0.76-1.04) | 0.09 (0.07-0.1) | 2.3 (1.96-2.64) | 0.09 (0.07-0.1) | -0.12 (-0.15 to -0.09) |
| Paraguay | 1.89 (1.62-2.17) | 0.1 (0.08-0.11) | 5.02 (4.33-5.69) | 0.1 (0.08-0.11) | 0.02 (-0.01 to 0.05) |
| Peru | 8.13 (6.97-9.38) | 0.08 (0.07-0.09) | 27.03 (23.33-30.77) | 0.08 (0.07-0.1) | 0.12 (0.09 to 0.14) |
| Philippines | 17.16 (14.65-19.98) | 0.08 (0.07-0.1) | 48.04 (41.35-54.65) | 0.08 (0.07-0.1) | -0.02 (-0.06 to 0.02) |
| Poland | 42.43 (35.22-49.65) | 0.11 (0.09-0.12) | 81.84 (69.08-93.65) | 0.11 (0.09-0.12) | 0.01 (-0.01 to 0.02) |
| Portugal | 11.73 (9.82-13.79) | 0.09 (0.08-0.1) | 26.42 (22.26-30.44) | 0.09 (0.08-0.1) | 0.01 (-0.01 to 0.04) |
| Puerto Rico | 2.72 (2.3-3.16) | 0.08 (0.07-0.09) | 6.98 (5.97-8.03) | 0.08 (0.07-0.09) | 0.07 (0.06 to 0.08) |
| Qatar | 0.05 (0.05-0.06) | 0.11 (0.09-0.12) | 0.43 (0.36-0.51) | 0.11 (0.09-0.12) | 0.11 (0.1 to 0.12) |
| South Korea | 20.49 (17.61-23.62) | 0.11 (0.09-0.12) | 83.49 (72.59-95.84) | 0.1 (0.09-0.11) | -0.13 (-0.17 to -0.1) |
| Moldova | 3.34 (2.79-3.92) | 0.1 (0.09-0.12) | 6.03 (5.13-6.9) | 0.1 (0.09-0.12) | 0.11 (0.09 to 0.12) |
| Romania | 23.13 (18.88-27.54) | 0.1 (0.09-0.12) | 43.07 (35.89-49.81) | 0.11 (0.09-0.12) | 0.13 (0.11 to 0.14) |
| Russia | 145.09 (118.15-172.62) | 0.1 (0.08-0.11) | 240.59 (200.6-277.66) | 0.1 (0.08-0.12) | 0.17 (0.13 to 0.22) |
| Rwanda | 1.56 (1.34-1.8) | 0.09 (0.07-0.1) | 3.42 (2.98-3.91) | 0.09 (0.08-0.1) | 0.07 (0.04 to 0.1) |
| Saint Kitts | 0.03 (0.02-0.03) | 0.08 (0.07-0.09) | 0.04 (0.03-0.04) | 0.08 (0.07-0.09) | 0 (-0.03 to 0.02) |
| Saint Lucia | 0.06 (0.05-0.07) | 0.08 (0.07-0.09) | 0.16 (0.14-0.18) | 0.08 (0.07-0.09) | 0.05 (0.03 to 0.07) |
| Saint Vincent | 0.05 (0.04-0.06) | 0.08 (0.07-0.09) | 0.1 (0.08-0.11) | 0.08 (0.07-0.09) | -0.02 (-0.04 to 0) |
| Samoa | 0.06 (0.05-0.07) | 0.09 (0.07-0.1) | 0.1 (0.09-0.12) | 0.09 (0.07-0.1) | -0.08 (-0.1 to -0.05) |
| San Marino | 0.03 (0.02-0.03) | 0.09 (0.08-0.1) | 0.07 (0.06-0.08) | 0.09 (0.07-0.1) | -0.02 (-0.04 to -0.01) |
| Sao Tome and Principe | 0.03 (0.03-0.04) | 0.07 (0.06-0.09) | 0.06 (0.05-0.06) | 0.08 (0.06-0.09) | 0.02 (-0.01 to 0.05) |
| Saudi Arabia | 4.32 (3.69-4.99) | 0.11 (0.09-0.13) | 10.23 (8.86-11.65) | 0.11 (0.09-0.12) | -0.05 (-0.07 to -0.04) |
| Senegal | 1.69 (1.44-1.95) | 0.08 (0.06-0.09) | 4.19 (3.61-4.79) | 0.08 (0.07-0.09) | 0.04 (0.01 to 0.07) |
| Serbia | 9.32 (7.75-10.86) | 0.1 (0.08-0.12) | 15.67 (12.73-18.58) | 0.1 (0.09-0.12) | 0.08 (0.07 to 0.1) |
| Seychelles | 0.05 (0.04-0.05) | 0.09 (0.07-0.1) | 0.08 (0.07-0.09) | 0.09 (0.07-0.1) | -0.05 (-0.07 to -0.03) |
| Sierra Leone | 1.09 (0.92-1.27) | 0.08 (0.06-0.09) | 1.92 (1.64-2.2) | 0.08 (0.06-0.09) | 0.02 (-0.01 to 0.05) |
| Singapore | 1.41 (1.21-1.64) | 0.09 (0.08-0.1) | 6.55 (5.7-7.45) | 0.09 (0.08-0.11) | 0.1 (0.06 to 0.15) |
| Slovakia | 5.63 (4.65-6.63) | 0.1 (0.09-0.12) | 9.64 (8.14-11.15) | 0.11 (0.09-0.12) | 0.11 (0.09 to 0.13) |
| Slovenia | 2.44 (2.02-2.85) | 0.11 (0.09-0.12) | 5.51 (4.67-6.32) | 0.11 (0.09-0.12) | 0.11 (0.1 to 0.11) |
| Solomon Islands | 0.06 (0.05-0.07) | 0.09 (0.07-0.1) | 0.14 (0.12-0.16) | 0.09 (0.07-0.1) | -0.02 (-0.06 to 0.01) |
| Somalia | 1.21 (1.04-1.38) | 0.09 (0.07-0.1) | 3.11 (2.67-3.59) | 0.09 (0.07-0.1) | 0.01 (-0.02 to 0.04) |
| South Africa | 15.11 (12.98-17.3) | 0.09 (0.08-0.1) | 31.6 (27.23-36.2) | 0.09 (0.08-0.1) | 0 (-0.05 to 0.05) |
| South Sudan | 1.46 (1.24-1.69) | 0.09 (0.07-0.1) | 2.18 (1.89-2.47) | 0.08 (0.07-0.1) | -0.11 (-0.15 to -0.07) |
| Spain | 55.58 (49.19-61.6) | 0.1 (0.09-0.11) | 104.96 (90.22-119.16) | 0.09 (0.08-0.1) | -0.31 (-0.37 to -0.26) |
| Sri Lanka | 6.54 (5.62-7.57) | 0.08 (0.07-0.1) | 18.43 (15.9-21.08) | 0.09 (0.07-0.1) | 0.11 (0.09 to 0.13) |
| Sudan | 7.19 (6.06-8.4) | 0.11 (0.09-0.13) | 14.9 (12.72-17.1) | 0.11 (0.09-0.12) | -0.06 (-0.1 to -0.03) |
| Suriname | 0.18 (0.16-0.21) | 0.08 (0.07-0.09) | 0.42 (0.36-0.49) | 0.08 (0.07-0.09) | 0.01 (-0.03 to 0.05) |
| Sweden | 16.16 (13.65-18.87) | 0.09 (0.08-0.11) | 21.37 (18.09-24.61) | 0.08 (0.07-0.1) | -0.27 (-0.32 to -0.22) |
| Switzerland | 10.5 (8.85-12.26) | 0.09 (0.08-0.11) | 18.7 (15.8-21.42) | 0.09 (0.08-0.1) | -0.06 (-0.07 to -0.05) |
| Syria | 4.29 (3.68-4.9) | 0.11 (0.09-0.13) | 9.41 (8-10.91) | 0.11 (0.09-0.13) | 0.03 (0.01 to 0.05) |
| Taiwan | 8.81 (7.42-10.18) | 0.08 (0.07-0.09) | 35.7 (30.85-39.86) | 0.09 (0.08-0.1) | 0.46 (0.36 to 0.56) |
| Tajikistan | 2.7 (2.3-3.08) | 0.1 (0.09-0.12) | 2.74 (2.35-3.16) | 0.1 (0.09-0.12) | 0.02 (-0.02 to 0.05) |
| Thailand | 21.52 (18.64-24.41) | 0.08 (0.07-0.09) | 85.48 (74.56-96.87) | 0.09 (0.08-0.1) | 0.25 (0.2 to 0.3) |
| Timor-Leste | 0.15 (0.13-0.17) | 0.09 (0.07-0.1) | 0.5 (0.42-0.57) | 0.08 (0.07-0.1) | -0.1 (-0.16 to -0.05) |
| Togo | 0.59 (0.51-0.68) | 0.08 (0.06-0.09) | 1.7 (1.48-1.96) | 0.08 (0.07-0.09) | 0.09 (0.06 to 0.13) |
| Tokelau | 0 (0-0) | 0.09 (0.07-0.1) | 0 (0-0) | 0.09 (0.07-0.1) | -0.05 (-0.06 to -0.04) |
| Tonga | 0.04 (0.03-0.04) | 0.09 (0.07-0.1) | 0.07 (0.06-0.08) | 0.09 (0.07-0.1) | -0.01 (-0.03 to 0.01) |
| Tobago | 0.53 (0.44-0.62) | 0.08 (0.07-0.09) | 1.36 (1.17-1.56) | 0.08 (0.07-0.09) | 0.08 (0.05 to 0.1) |
| Tunisia | 3.96 (3.34-4.63) | 0.11 (0.09-0.13) | 12.03 (10.26-13.81) | 0.11 (0.09-0.13) | 0 (-0.01 to 0.02) |
| Turkey | 31.64 (27.03-36.25) | 0.11 (0.09-0.13) | 90.96 (78.27-103.57) | 0.11 (0.1-0.13) | 0.06 (0.05 to 0.07) |
| Turkmenistan | 1.44 (1.23-1.66) | 0.1 (0.09-0.12) | 2.97 (2.55-3.38) | 0.1 (0.09-0.12) | 0.02 (0 to 0.05) |
| Tuvalu | 0 (0-0) | 0.09 (0.08-0.1) | 0.01 (0.01-0.01) | 0.09 (0.07-0.1) | -0.15 (-0.19 to -0.1) |
| Uganda | 3.71 (3.2-4.27) | 0.09 (0.07-0.1) | 8.19 (7.11-9.33) | 0.09 (0.07-0.1) | 0.02 (0 to 0.04) |
| Ukraine | 61.96 (50.48-73.77) | 0.1 (0.08-0.11) | 80.87 (67.04-94.58) | 0.1 (0.09-0.12) | 0.17 (0.16 to 0.19) |
| United Arab Emirates | 0.18 (0.15-0.21) | 0.1 (0.09-0.12) | 1.37 (1.11-1.62) | 0.1 (0.09-0.12) | -0.06 (-0.08 to -0.04) |
| UK | 82.34 (69.47-96.09) | 0.09 (0.07-0.1) | 120.42 (101.72-138.78) | 0.08 (0.07-0.1) | -0.07 (-0.1 to -0.04) |
| Tanzania | 6.02 (5.15-6.96) | 0.09 (0.07-0.1) | 14.6 (12.81-16.51) | 0.08 (0.07-0.09) | -0.1 (-0.12 to -0.07) |
| USA | 384.24 (324.5-440.43) | 0.11 (0.1-0.13) | 665.94 (592.55-732.32) | 0.11 (0.09-0.12) | -0.01 (-0.06 to 0.04) |
| Virgin Islands | 0.05 (0.04-0.06) | 0.08 (0.07-0.09) | 0.13 (0.11-0.15) | 0.08 (0.07-0.09) | 0.04 (0.02 to 0.06) |
| Uruguay | 3.61 (3.04-4.23) | 0.09 (0.08-0.11) | 6.19 (5.2-7.08) | 0.1 (0.08-0.11) | 0.06 (0.05 to 0.08) |
| Uzbekistan | 9.74 (8.28-11.18) | 0.1 (0.09-0.12) | 9.88 (8.29-11.58) | 0.1 (0.09-0.12) | 0.03 (-0.01 to 0.06) |
| Vanuatu | 0.03 (0.03-0.04) | 0.08 (0.07-0.1) | 0.1 (0.08-0.11) | 0.08 (0.07-0.1) | -0.04 (-0.08 to -0.01) |
| Venezuela | 6.66 (5.71-7.7) | 0.09 (0.07-0.1) | 22.38 (19.28-25.53) | 0.08 (0.07-0.1) | -0.05 (-0.07 to -0.02) |
| Vietnam | 28.85 (24.66-33.04) | 0.09 (0.07-0.1) | 65.44 (56.56-74.35) | 0.09 (0.07-0.1) | 0 (-0.03 to 0.03) |
| Yemen | 3.38 (2.85-3.9) | 0.11 (0.1-0.13) | 9.72 (8.28-11.15) | 0.11 (0.09-0.12) | -0.11 (-0.15 to -0.07) |
| Zambia | 1.47 (1.25-1.69) | 0.08 (0.07-0.09) | 3.58 (3.1-4.08) | 0.08 (0.07-0.1) | 0.09 (0.05 to 0.13) |
| Zimbabwe | 2.35 (2.02-2.72) | 0.09 (0.08-0.1) | 3.96 (3.42-4.55) | 0.09 (0.08-0.1) | -0.05 (-0.1 to 0) |

ASIR, age-standardized incidence rate; EAPC, estimated annual percent change;

**Supplement Table 2.** Prevalence numbers and age-standardized prevalence rates (ASPR) of Alzheimer's disease and other dementias in 1990 and 2019 and the temporal trends from 1990 to 2019 in 204 countries.

|  | **1990** | | **2019** | | **EAPC of ASPR from 1990 to 2019** |
| --- | --- | --- | --- | --- | --- |
| **location** | **Prevalence cases (*10^3)** | **ASPR (*10^3)** | **Prevalence cases (*10^3)** | **ASPR (*10^3)** |  |
| Afghanistan | 35.55 (29.74-41.49) | 0.75 (0.63-0.86) | 54.77 (45.89-63.56) | 0.74 (0.62-0.85) | -0.07 (-0.12 to -0.02) |
| Albania | 11.98 (10.18-13.79) | 0.75 (0.63-0.87) | 31.87 (26.65-37.2) | 0.77 (0.65-0.89) | 0.09 (0.08 to 0.09) |
| Algeria | 58.21 (48.15-68.88) | 0.77 (0.65-0.89) | 193.06 (162.81-224.02) | 0.78 (0.66-0.9) | 0.04 (0.02 to 0.06) |
| American Samoa | 0.08 (0.07-0.09) | 0.58 (0.48-0.67) | 0.22 (0.18-0.25) | 0.59 (0.49-0.68) | 0.06 (0.05 to 0.06) |
| Andorra | 0.25 (0.21-0.3) | 0.63 (0.53-0.73) | 0.98 (0.84-1.13) | 0.64 (0.54-0.74) | 0.04 (0.04 to 0.05) |
| Angola | 13.78 (11.72-16.01) | 0.67 (0.57-0.78) | 42.98 (36.65-49.81) | 0.69 (0.59-0.8) | 0.13 (0.1 to 0.15) |
| Antigua | 0.31 (0.26-0.36) | 0.53 (0.45-0.61) | 0.47 (0.4-0.54) | 0.54 (0.46-0.63) | 0.11 (0.1 to 0.12) |
| Argentina | 179.78 (149.56-211.08) | 0.64 (0.54-0.75) | 378.29 (320.88-439.58) | 0.67 (0.57-0.78) | 0.14 (0.14 to 0.15) |
| Armenia | 13.85 (11.74-16.09) | 0.7 (0.59-0.81) | 28.69 (24.06-33.31) | 0.74 (0.62-0.85) | 0.21 (0.19 to 0.22) |
| Australia | 124.89 (106.19-144.22) | 0.68 (0.58-0.78) | 318.72 (271.95-366.2) | 0.67 (0.57-0.78) | -0.03 (-0.04 to -0.01) |
| Austria | 80.07 (66.7-94.06) | 0.64 (0.54-0.74) | 135.84 (113.85-157.66) | 0.63 (0.53-0.73) | -0.05 (-0.06 to -0.04) |
| Azerbaijan | 29.38 (24.95-33.86) | 0.7 (0.59-0.81) | 39.64 (33.08-46.36) | 0.72 (0.6-0.83) | 0.06 (0.03 to 0.1) |
| Bahamas | 0.64 (0.55-0.74) | 0.52 (0.44-0.6) | 1.73 (1.48-2) | 0.54 (0.45-0.62) | 0.08 (0.07 to 0.09) |
| Bahrain | 0.68 (0.57-0.8) | 0.77 (0.65-0.9) | 3.76 (3.2-4.35) | 0.8 (0.68-0.93) | 0.15 (0.14 to 0.16) |
| Bangladesh | 165.58 (140.44-192.01) | 0.49 (0.41-0.56) | 539.35 (457.71-624.2) | 0.51 (0.43-0.58) | 0.14 (0.11 to 0.17) |
| Barbados | 1.65 (1.4-1.94) | 0.53 (0.45-0.61) | 2.7 (2.31-3.13) | 0.55 (0.47-0.63) | 0.12 (0.11 to 0.13) |
| Belarus | 84.93 (71.05-99.22) | 0.71 (0.6-0.83) | 123.39 (103.02-144.13) | 0.74 (0.62-0.86) | 0.18 (0.14 to 0.21) |
| Belgium | 106.91 (89.98-124.94) | 0.67 (0.57-0.78) | 178.61 (149.17-208.74) | 0.63 (0.54-0.74) | -0.25 (-0.29 to -0.22) |
| Belize | 0.46 (0.39-0.53) | 0.53 (0.45-0.61) | 1.23 (1.05-1.41) | 0.54 (0.46-0.62) | 0.07 (0.05 to 0.08) |
| Benin | 7.58 (6.47-8.77) | 0.48 (0.41-0.55) | 16.37 (14.15-18.82) | 0.48 (0.41-0.55) | 0.05 (0 to 0.11) |
| Bermuda | 0.3 (0.25-0.35) | 0.55 (0.47-0.63) | 0.82 (0.7-0.95) | 0.58 (0.49-0.66) | 0.19 (0.18 to 0.21) |
| Bhutan | 0.69 (0.59-0.79) | 0.45 (0.39-0.53) | 2.15 (1.84-2.47) | 0.48 (0.41-0.55) | 0.19 (0.17 to 0.22) |
| Bolivia | 12.08 (10.32-13.99) | 0.52 (0.45-0.61) | 38.72 (32.9-44.56) | 0.55 (0.47-0.64) | 0.2 (0.18 to 0.22) |
| Bosnia and Herzegovina | 20.4 (16.97-23.81) | 0.74 (0.61-0.86) | 40.95 (33.95-48.4) | 0.74 (0.62-0.87) | 0.07 (0.05 to 0.08) |
| Botswana | 2.19 (1.87-2.54) | 0.61 (0.52-0.7) | 5.23 (4.48-6.07) | 0.62 (0.53-0.72) | 0.07 (0.04 to 0.09) |
| Brazil | 472.67 (405.12-544.18) | 0.72 (0.62-0.82) | 1702.4 (1467.81-1939.72) | 0.77 (0.66-0.88) | 0.34 (0.29 to 0.38) |
| Brunei | 0.32 (0.27-0.38) | 0.65 (0.55-0.76) | 1 (0.84-1.17) | 0.66 (0.56-0.76) | 0.01 (-0.07 to 0.09) |
| Bulgaria | 67.17 (54.94-80.34) | 0.72 (0.59-0.83) | 113.39 (93.32-134.12) | 0.74 (0.62-0.86) | 0.13 (0.12 to 0.14) |
| Burkina Faso | 14.47 (12.26-16.83) | 0.51 (0.43-0.58) | 31.83 (27.28-36.64) | 0.52 (0.44-0.59) | 0.08 (0.06 to 0.09) |
| Burundi | 9.35 (7.95-10.93) | 0.58 (0.49-0.67) | 15.7 (13.57-18.02) | 0.57 (0.49-0.65) | -0.03 (-0.04 to -0.01) |
| Cape Verde | 1.23 (1.04-1.43) | 0.51 (0.44-0.59) | 2.18 (1.88-2.5) | 0.54 (0.46-0.61) | 0.18 (0.16 to 0.2) |
| Cambodia | 16.92 (14.36-19.63) | 0.57 (0.49-0.66) | 51.67 (43.76-59.24) | 0.59 (0.51-0.68) | 0.14 (0.1 to 0.17) |
| Cameroon | 13.87 (11.81-16.07) | 0.47 (0.4-0.54) | 38.03 (32.56-44) | 0.49 (0.42-0.56) | 0.15 (0.13 to 0.18) |
| Canada | 231.41 (195.97-268.97) | 0.74 (0.63-0.85) | 532.69 (452.31-612.09) | 0.69 (0.58-0.79) | -0.27 (-0.36 to -0.18) |
| Central African Republic | 4.25 (3.62-4.92) | 0.71 (0.6-0.82) | 7.93 (6.81-9.15) | 0.73 (0.62-0.84) | 0.06 (0.04 to 0.08) |
| Chad | 10.64 (9.08-12.37) | 0.49 (0.42-0.57) | 18.57 (15.95-21.34) | 0.49 (0.42-0.56) | -0.06 (-0.09 to -0.03) |
| Chile | 48.68 (41.15-56.26) | 0.61 (0.51-0.7) | 159.61 (134.48-185.15) | 0.67 (0.56-0.77) | 0.39 (0.35 to 0.43) |
| China | 3345.97 (2797.32-3890.88) | 0.61 (0.52-0.71) | 13143.95 (11021.21-15299.02) | 0.79 (0.66-0.91) | 0.66 (0.57 to 0.75) |
| Colombia | 75.38 (64.93-87.22) | 0.56 (0.48-0.64) | 328.72 (283.32-376.23) | 0.59 (0.51-0.68) | 0.2 (0.19 to 0.21) |
| Comoros | 0.92 (0.78-1.07) | 0.57 (0.48-0.65) | 2.22 (1.91-2.59) | 0.57 (0.49-0.66) | 0.06 (0.03 to 0.08) |
| Republic of Congo | 4.13 (3.46-4.87) | 0.67 (0.57-0.78) | 11.08 (9.84-12.3) | 0.71 (0.63-0.79) | 0.29 (0.19 to 0.38) |
| Cook Islands | 0.06 (0.05-0.06) | 0.59 (0.5-0.68) | 0.14 (0.12-0.16) | 0.6 (0.5-0.69) | 0.04 (0.04 to 0.05) |
| Costa Rica | 8.75 (7.52-10.17) | 0.56 (0.48-0.65) | 30.17 (25.95-34.52) | 0.59 (0.51-0.68) | 0.14 (0.14 to 0.15) |
| Croatia | 40.16 (33.43-47.32) | 0.75 (0.63-0.88) | 74.85 (62.15-88.27) | 0.77 (0.65-0.91) | 0.12 (0.11 to 0.13) |
| Cuba | 48.63 (41.55-56.29) | 0.51 (0.44-0.58) | 107.8 (93.23-123.03) | 0.52 (0.45-0.6) | 0.11 (0.11 to 0.12) |
| Cyprus | 3.82 (3.15-4.52) | 0.6 (0.51-0.7) | 11.42 (9.54-13.37) | 0.63 (0.54-0.73) | 0.26 (0.24 to 0.29) |
| Czech Republic | 93.88 (77.25-110.5) | 0.73 (0.61-0.85) | 175.25 (146.96-205.01) | 0.77 (0.65-0.9) | 0.21 (0.2 to 0.22) |
| Ivory Coast | 10.44 (8.87-12.1) | 0.48 (0.4-0.55) | 31.68 (26.92-36.4) | 0.49 (0.41-0.57) | 0.09 (0.06 to 0.12) |
| North Korea | 65.77 (55.69-76.43) | 0.65 (0.55-0.75) | 172.37 (145.16-201.66) | 0.63 (0.53-0.74) | -0.06 (-0.11 to -0.01) |
| Democratic Republic of the Congo | 56.13 (47.69-65.58) | 0.66 (0.56-0.76) | 151.88 (129.6-174.68) | 0.69 (0.59-0.8) | 0.2 (0.2 to 0.21) |
| Denmark | 56.75 (48.17-66.13) | 0.64 (0.54-0.74) | 77.24 (65.57-89.24) | 0.59 (0.51-0.69) | -0.27 (-0.3 to -0.25) |
| Djibouti | 0.41 (0.35-0.47) | 0.58 (0.5-0.68) | 1.91 (1.64-2.18) | 0.57 (0.49-0.65) | -0.05 (-0.08 to -0.01) |
| Dominica | 0.4 (0.33-0.46) | 0.53 (0.45-0.61) | 0.49 (0.41-0.57) | 0.53 (0.45-0.61) | 0.01 (0 to 0.01) |
| Dominican Republic | 14.97 (12.85-17.29) | 0.51 (0.44-0.59) | 43.8 (37.73-50.4) | 0.52 (0.45-0.6) | 0.08 (0.07 to 0.09) |
| Ecuador | 23.26 (19.83-27.1) | 0.54 (0.46-0.62) | 75.93 (64.81-87.77) | 0.57 (0.49-0.66) | 0.22 (0.21 to 0.23) |
| Egypt | 133.58 (113.58-154.05) | 0.71 (0.61-0.82) | 295.76 (250.88-342.57) | 0.75 (0.64-0.87) | 0.25 (0.21 to 0.3) |
| El Salvador | 14.89 (12.7-17.09) | 0.55 (0.47-0.64) | 37.79 (32.39-43.74) | 0.58 (0.5-0.67) | 0.14 (0.14 to 0.15) |
| Equatorial Guinea | 0.78 (0.66-0.91) | 0.67 (0.57-0.77) | 2.2 (1.87-2.55) | 0.7 (0.6-0.81) | 0.2 (0.18 to 0.22) |
| Eritrea | 2.8 (2.39-3.25) | 0.6 (0.51-0.69) | 8.5 (7.28-9.81) | 0.59 (0.51-0.68) | -0.06 (-0.08 to -0.03) |
| Estonia | 13.14 (10.87-15.62) | 0.69 (0.58-0.82) | 24.05 (20.14-28.06) | 0.75 (0.64-0.87) | 0.37 (0.34 to 0.4) |
| Swaziland | 1.14 (0.97-1.31) | 0.6 (0.51-0.7) | 2.18 (1.85-2.56) | 0.61 (0.52-0.7) | 0 (-0.06 to 0.06) |
| Ethiopia | 62.08 (52.81-72.33) | 0.59 (0.5-0.67) | 168.3 (144.58-193.19) | 0.57 (0.49-0.66) | -0.05 (-0.11 to 0.02) |
| Fiji | 1.19 (1-1.39) | 0.58 (0.49-0.67) | 2.72 (2.29-3.16) | 0.59 (0.5-0.68) | 0.04 (0.03 to 0.06) |
| Finland | 45.89 (38.63-53.69) | 0.65 (0.55-0.75) | 91.95 (77.26-107.11) | 0.62 (0.53-0.73) | -0.13 (-0.14 to -0.11) |
| France | 523.62 (453.77-601.71) | 0.58 (0.5-0.66) | 1124.12 (947.77-1308.98) | 0.64 (0.54-0.74) | 0.38 (0.31 to 0.46) |
| Gabon | 2.74 (2.32-3.18) | 0.69 (0.59-0.8) | 5.09 (4.35-5.87) | 0.7 (0.6-0.81) | 0.07 (0.04 to 0.1) |
| Gambia | 1.11 (0.94-1.29) | 0.49 (0.42-0.57) | 3.56 (3.02-4.13) | 0.5 (0.42-0.57) | 0.03 (0 to 0.06) |
| Georgia | 34.67 (29.15-40.54) | 0.71 (0.61-0.83) | 49.84 (42.02-57.75) | 0.72 (0.61-0.83) | 0.01 (0 to 0.02) |
| Germany | 875.62 (774.67-975.06) | 0.66 (0.59-0.73) | 1582.45 (1354.53-1837.74) | 0.67 (0.58-0.78) | 0.09 (0.04 to 0.13) |
| Ghana | 18.44 (15.8-21.36) | 0.47 (0.4-0.54) | 53.29 (45.72-61.35) | 0.49 (0.42-0.57) | 0.14 (0.12 to 0.17) |
| Greece | 91.36 (76.28-107.36) | 0.63 (0.53-0.73) | 193.78 (162.21-227.52) | 0.63 (0.53-0.73) | 0.01 (0 to 0.02) |
| Greenland | 0.14 (0.12-0.17) | 0.72 (0.6-0.84) | 0.37 (0.31-0.43) | 0.73 (0.61-0.86) | 0.07 (0.04 to 0.1) |
| Grenada | 0.44 (0.37-0.51) | 0.52 (0.44-0.6) | 0.49 (0.42-0.57) | 0.54 (0.46-0.62) | 0.12 (0.11 to 0.13) |
| Guam | 0.26 (0.22-0.3) | 0.59 (0.5-0.69) | 1.09 (0.92-1.25) | 0.6 (0.51-0.69) | 0 (-0.01 to 0.01) |
| Guatemala | 13.01 (11.05-15.06) | 0.54 (0.46-0.62) | 54.14 (46.1-62.84) | 0.57 (0.49-0.66) | 0.21 (0.21 to 0.22) |
| Guinea | 11.97 (10.23-13.79) | 0.48 (0.41-0.55) | 20.68 (17.6-23.68) | 0.49 (0.42-0.56) | 0.06 (0.04 to 0.08) |
| Guinea-Bissau | 1.2 (1.01-1.39) | 0.48 (0.41-0.55) | 2.15 (1.83-2.49) | 0.49 (0.42-0.57) | 0.11 (0.08 to 0.15) |
| Guyana | 1.39 (1.18-1.61) | 0.5 (0.42-0.58) | 2.41 (2.05-2.77) | 0.52 (0.44-0.6) | 0.11 (0.09 to 0.13) |
| Haiti | 10.42 (8.8-12.17) | 0.5 (0.42-0.58) | 23.96 (20.25-27.75) | 0.51 (0.43-0.59) | 0.03 (0 to 0.06) |
| Honduras | 8.8 (7.53-10.17) | 0.54 (0.46-0.63) | 26.4 (22.36-30.72) | 0.56 (0.48-0.65) | 0.07 (0.04 to 0.1) |
| Hungary | 95.31 (78.48-112.62) | 0.72 (0.6-0.84) | 163.72 (136.26-191.8) | 0.77 (0.64-0.89) | 0.23 (0.22 to 0.24) |
| Iceland | 1.93 (1.61-2.25) | 0.63 (0.53-0.73) | 4.05 (3.4-4.71) | 0.63 (0.53-0.73) | 0.01 (0 to 0.01) |
| India | 1062.26 (901.54-1226.96) | 0.4 (0.34-0.46) | 3692.64 (3132.56-4249.26) | 0.42 (0.35-0.48) | 0.15 (0.11 to 0.19) |
| Indonesia | 357.16 (303.97-411.98) | 0.55 (0.47-0.64) | 836.97 (710.07-967.34) | 0.56 (0.48-0.65) | 0.05 (0.01 to 0.09) |
| Iran | 116.63 (99.03-134.46) | 0.78 (0.66-0.9) | 470.95 (397.87-542.86) | 0.79 (0.67-0.91) | 0.03 (0.01 to 0.05) |
| Iraq | 44.76 (37.79-52.19) | 0.74 (0.62-0.86) | 118.87 (101.49-137.3) | 0.76 (0.65-0.88) | 0.15 (0.11 to 0.19) |
| Ireland | 24.02 (19.95-28.45) | 0.62 (0.52-0.72) | 49.49 (41.71-57.59) | 0.63 (0.53-0.73) | 0.05 (0.04 to 0.05) |
| Israel | 27.48 (22.9-32.66) | 0.61 (0.51-0.71) | 79.41 (67.21-91.99) | 0.62 (0.53-0.72) | 0.12 (0.11 to 0.13) |
| Italy | 649.19 (540.79-760.51) | 0.74 (0.62-0.85) | 1369.01 (1148.21-1604.01) | 0.74 (0.63-0.86) | -0.08 (-0.16 to -0.01) |
| Jamaica | 9.67 (8.25-11.27) | 0.53 (0.45-0.61) | 17.78 (15.31-20.49) | 0.54 (0.46-0.62) | 0.09 (0.08 to 0.09) |
| Japan | 971.16 (830.64-1130.66) | 0.64 (0.55-0.74) | 3934.72 (3322.35-4579.24) | 0.78 (0.67-0.91) | 0.77 (0.71 to 0.83) |
| Jordan | 6 (5.08-6.98) | 0.74 (0.62-0.85) | 32 (26.99-37.37) | 0.76 (0.65-0.89) | 0.16 (0.13 to 0.18) |
| Kazakhstan | 68.27 (57.35-79.92) | 0.69 (0.58-0.81) | 88.82 (74.62-104.23) | 0.71 (0.6-0.83) | 0.14 (0.1 to 0.17) |
| Kenya | 32.05 (27.45-36.92) | 0.56 (0.48-0.65) | 81.12 (69.93-92.96) | 0.58 (0.5-0.67) | 0.11 (0.09 to 0.12) |
| Kiribati | 0.11 (0.1-0.13) | 0.57 (0.49-0.67) | 0.2 (0.17-0.24) | 0.57 (0.48-0.66) | -0.01 (-0.03 to 0.01) |
| Kuwait | 2.85 (2.44-3.27) | 0.79 (0.67-0.91) | 14.28 (12.28-16.37) | 0.8 (0.68-0.92) | 0.06 (0.03 to 0.08) |
| Kyrgyzstan | 19.12 (16.14-22.32) | 0.69 (0.58-0.8) | 24.52 (20.92-28.37) | 0.72 (0.61-0.84) | 0.17 (0.15 to 0.2) |
| Laos | 7.46 (6.27-8.71) | 0.56 (0.48-0.65) | 17.33 (14.7-19.98) | 0.57 (0.48-0.65) | 0.05 (0 to 0.1) |
| Latvia | 23.46 (19.37-27.88) | 0.69 (0.57-0.81) | 34.58 (28.73-41.14) | 0.73 (0.61-0.85) | 0.23 (0.21 to 0.24) |
| Lebanon | 12.44 (10.55-14.4) | 0.76 (0.64-0.88) | 39.91 (34.07-46.45) | 0.79 (0.68-0.92) | 0.15 (0.13 to 0.16) |
| Lesotho | 4.28 (3.65-4.94) | 0.61 (0.52-0.71) | 4.84 (4.11-5.59) | 0.6 (0.51-0.69) | -0.09 (-0.14 to -0.04) |
| Liberia | 3.75 (3.18-4.37) | 0.47 (0.4-0.54) | 6.81 (5.88-7.79) | 0.49 (0.42-0.56) | 0.14 (0.12 to 0.16) |
| Libya | 11.33 (9.62-13.11) | 0.76 (0.65-0.89) | 30.49 (25.76-35.24) | 0.76 (0.64-0.87) | -0.05 (-0.07 to -0.03) |
| Lithuania | 31.19 (26.06-36.66) | 0.71 (0.6-0.83) | 50.43 (41.92-59.31) | 0.73 (0.62-0.85) | 0.13 (0.11 to 0.14) |
| Luxembourg | 3.37 (2.83-4) | 0.63 (0.53-0.73) | 6.33 (5.37-7.32) | 0.56 (0.47-0.64) | -0.43 (-0.54 to -0.31) |
| Madagascar | 19.14 (16.44-22.1) | 0.54 (0.47-0.63) | 34.62 (29.8-39.93) | 0.56 (0.48-0.64) | 0.08 (0.06 to 0.1) |
| Malawi | 13.45 (11.49-15.62) | 0.57 (0.49-0.65) | 29.07 (24.93-33.53) | 0.59 (0.5-0.68) | 0.13 (0.12 to 0.14) |
| Malaysia | 41.2 (35.09-47.42) | 0.59 (0.5-0.68) | 128.53 (109.25-148.17) | 0.61 (0.52-0.7) | 0.13 (0.12 to 0.14) |
| Maldives | 0.29 (0.25-0.34) | 0.55 (0.47-0.64) | 1.43 (1.23-1.64) | 0.61 (0.52-0.7) | 0.38 (0.36 to 0.41) |
| Mali | 13.17 (11.16-15.26) | 0.49 (0.42-0.57) | 29.9 (25.57-34.45) | 0.5 (0.43-0.57) | 0.04 (0.02 to 0.06) |
| Malta | 2.38 (2-2.8) | 0.62 (0.53-0.73) | 6.53 (5.5-7.62) | 0.63 (0.53-0.73) | 0.04 (0.03 to 0.05) |
| Marshall Islands | 0.06 (0.05-0.07) | 0.57 (0.48-0.66) | 0.1 (0.08-0.11) | 0.55 (0.46-0.64) | -0.13 (-0.18 to -0.09) |
| Mauritania | 3.46 (2.93-4.01) | 0.47 (0.41-0.55) | 7.94 (6.82-9.18) | 0.49 (0.42-0.56) | 0.09 (0.07 to 0.12) |
| Mauritius | 3.22 (2.72-3.72) | 0.59 (0.5-0.69) | 9.44 (8.08-10.8) | 0.61 (0.52-0.69) | 0.05 (0.04 to 0.06) |
| Mexico | 177.32 (150.82-205.65) | 0.53 (0.45-0.61) | 537.54 (461.3-618.29) | 0.51 (0.44-0.59) | 0.01 (-0.05 to 0.07) |
| Micronesia | 0.16 (0.13-0.19) | 0.57 (0.48-0.66) | 0.23 (0.19-0.27) | 0.58 (0.48-0.67) | 0.08 (0.06 to 0.1) |
| Monaco | 0.54 (0.45-0.63) | 0.63 (0.53-0.73) | 0.74 (0.62-0.86) | 0.62 (0.52-0.72) | -0.06 (-0.07 to -0.04) |
| Mongolia | 5.13 (4.32-6.04) | 0.69 (0.58-0.8) | 9.45 (7.93-11.01) | 0.71 (0.61-0.82) | 0.17 (0.14 to 0.2) |
| Montenegro | 4.05 (3.39-4.72) | 0.75 (0.63-0.87) | 6.86 (5.73-8.04) | 0.76 (0.64-0.88) | 0.07 (0.06 to 0.08) |
| Morocco | 71.27 (60.29-83.12) | 0.76 (0.64-0.88) | 174.58 (147.73-202.32) | 0.76 (0.64-0.88) | 0 (-0.03 to 0.04) |
| Mozambique | 21.28 (18.1-24.69) | 0.57 (0.49-0.66) | 40.91 (34.94-47.27) | 0.59 (0.5-0.67) | 0.08 (0.06 to 0.09) |
| Myanmar | 89.33 (75.56-104.06) | 0.57 (0.48-0.66) | 209.75 (177.49-241.23) | 0.59 (0.5-0.68) | 0.1 (0.07 to 0.14) |
| Namibia | 2.93 (2.48-3.43) | 0.58 (0.5-0.68) | 6.56 (5.59-7.61) | 0.61 (0.52-0.7) | 0.17 (0.12 to 0.21) |
| Nauru | 0.01 (0.01-0.01) | 0.57 (0.48-0.66) | 0.01 (0.01-0.01) | 0.57 (0.48-0.66) | -0.04 (-0.07 to -0.01) |
| Nepal | 26.89 (22.9-31.32) | 0.45 (0.39-0.52) | 78.4 (66.82-90.5) | 0.47 (0.4-0.54) | 0.11 (0.08 to 0.13) |
| Netherlands | 136.5 (119.82-152.12) | 0.66 (0.58-0.73) | 258.17 (220.33-296.58) | 0.68 (0.58-0.78) | 0.22 (0.17 to 0.27) |
| New Zealand | 25.74 (21.74-29.82) | 0.68 (0.58-0.79) | 60.62 (51.52-70.14) | 0.69 (0.59-0.8) | 0.04 (0.02 to 0.05) |
| Nicaragua | 6.84 (5.89-7.88) | 0.57 (0.49-0.66) | 20.36 (17.51-23.43) | 0.59 (0.51-0.68) | 0.14 (0.12 to 0.15) |
| Niger | 8.12 (6.89-9.4) | 0.5 (0.43-0.58) | 24.62 (21.26-28.37) | 0.51 (0.43-0.58) | 0.06 (0.02 to 0.11) |
| Nigeria | 148.26 (127.38-170.53) | 0.48 (0.41-0.55) | 266.56 (230.42-304.55) | 0.44 (0.38-0.5) | -0.34 (-0.48 to -0.19) |
| Niue | 0.01 (0.01-0.02) | 0.6 (0.51-0.7) | 0.01 (0.01-0.01) | 0.6 (0.51-0.69) | 0 (-0.02 to 0.02) |
| North Macedonia | 9.82 (8.18-11.52) | 0.71 (0.59-0.83) | 18.71 (15.53-22.08) | 0.75 (0.63-0.87) | 0.23 (0.21 to 0.24) |
| Northern Mariana Islands | 0.05 (0.04-0.06) | 0.59 (0.5-0.68) | 0.19 (0.16-0.22) | 0.6 (0.51-0.7) | 0.09 (0.08 to 0.09) |
| Norway | 51.04 (43.33-59.65) | 0.67 (0.57-0.77) | 71.11 (60.38-82.46) | 0.64 (0.54-0.74) | -0.13 (-0.15 to -0.1) |
| Oman | 2.77 (2.34-3.23) | 0.78 (0.66-0.91) | 6.75 (5.73-7.78) | 0.79 (0.67-0.91) | 0.02 (0 to 0.03) |
| Pakistan | 197.79 (168.4-228.75) | 0.44 (0.37-0.5) | 323.67 (275.9-373.43) | 0.45 (0.38-0.51) | 0.07 (0.03 to 0.12) |
| Palau | 0.04 (0.03-0.04) | 0.58 (0.49-0.68) | 0.08 (0.07-0.09) | 0.59 (0.5-0.68) | 0.02 (0 to 0.04) |
| Palestine | 5.1 (4.35-5.9) | 0.76 (0.64-0.88) | 12.04 (10.29-13.96) | 0.77 (0.65-0.89) | 0.06 (0.04 to 0.09) |
| Panama | 7.45 (6.4-8.56) | 0.56 (0.48-0.64) | 24.87 (21.41-28.71) | 0.58 (0.5-0.68) | 0.13 (0.12 to 0.15) |
| Papua New Guinea | 5.75 (4.82-6.71) | 0.58 (0.49-0.68) | 14.84 (12.52-17.25) | 0.57 (0.48-0.66) | -0.08 (-0.11 to -0.06) |
| Paraguay | 13.12 (11.24-15.12) | 0.68 (0.58-0.78) | 35.78 (30.9-40.96) | 0.7 (0.6-0.81) | 0.1 (0.08 to 0.12) |
| Peru | 52.35 (45.18-59.89) | 0.51 (0.44-0.59) | 180 (155.54-207.06) | 0.56 (0.48-0.64) | 0.27 (0.26 to 0.28) |
| Philippines | 115 (97.87-132.98) | 0.56 (0.48-0.65) | 326.27 (277.87-375.19) | 0.57 (0.49-0.66) | 0.05 (0.02 to 0.08) |
| Poland | 294.03 (245.63-345.01) | 0.76 (0.64-0.89) | 585.85 (491.38-683.16) | 0.77 (0.65-0.9) | 0.04 (0.03 to 0.05) |
| Portugal | 79.25 (66.61-93.03) | 0.63 (0.53-0.73) | 190.1 (160.26-221.88) | 0.64 (0.55-0.74) | 0.11 (0.1 to 0.12) |
| Puerto Rico | 18.56 (15.81-21.63) | 0.54 (0.46-0.62) | 48.41 (41.47-55.56) | 0.57 (0.49-0.65) | 0.17 (0.17 to 0.18) |
| Qatar | 0.34 (0.29-0.4) | 0.75 (0.63-0.87) | 2.7 (2.25-3.17) | 0.78 (0.65-0.91) | 0.17 (0.15 to 0.18) |
| South Korea | 134.16 (115.51-152.74) | 0.73 (0.63-0.84) | 597.51 (520.82-685.44) | 0.7 (0.61-0.81) | 0 (-0.05 to 0.04) |
| Moldova | 22.45 (18.79-26.35) | 0.7 (0.59-0.82) | 42.16 (35.88-48.98) | 0.73 (0.62-0.85) | 0.16 (0.15 to 0.17) |
| Romania | 157.42 (129.24-186.75) | 0.71 (0.59-0.83) | 306.85 (254.04-360.19) | 0.75 (0.63-0.87) | 0.21 (0.2 to 0.21) |
| Russia | 984.92 (815.52-1169.09) | 0.68 (0.57-0.8) | 1686.58 (1405.67-1975.66) | 0.71 (0.59-0.83) | 0.23 (0.19 to 0.28) |
| Rwanda | 10.36 (8.85-12.06) | 0.57 (0.49-0.66) | 23.09 (19.9-26.64) | 0.6 (0.51-0.69) | 0.2 (0.16 to 0.23) |
| Saint Kitts | 0.18 (0.15-0.21) | 0.53 (0.45-0.61) | 0.26 (0.22-0.3) | 0.54 (0.46-0.63) | 0.07 (0.06 to 0.09) |
| Saint Lucia | 0.4 (0.34-0.46) | 0.52 (0.45-0.6) | 1.09 (0.93-1.26) | 0.54 (0.46-0.62) | 0.12 (0.11 to 0.12) |
| Saint Vincent | 0.34 (0.29-0.4) | 0.53 (0.45-0.61) | 0.65 (0.55-0.75) | 0.53 (0.46-0.62) | 0.02 (0.02 to 0.03) |
| Samoa | 0.38 (0.32-0.44) | 0.59 (0.5-0.68) | 0.68 (0.58-0.79) | 0.58 (0.49-0.68) | -0.05 (-0.07 to -0.04) |
| San Marino | 0.2 (0.17-0.24) | 0.62 (0.52-0.73) | 0.5 (0.42-0.59) | 0.63 (0.53-0.73) | 0.06 (0.05 to 0.07) |
| Sao Tome and Principe | 0.23 (0.2-0.27) | 0.48 (0.41-0.56) | 0.37 (0.32-0.43) | 0.49 (0.42-0.57) | 0.09 (0.06 to 0.13) |
| Saudi Arabia | 29.07 (24.59-33.72) | 0.76 (0.65-0.89) | 67.81 (58.04-77.81) | 0.77 (0.65-0.89) | 0.03 (0.01 to 0.05) |
| Senegal | 11.04 (9.34-12.75) | 0.48 (0.41-0.56) | 27.57 (23.57-31.74) | 0.5 (0.42-0.57) | 0.1 (0.08 to 0.12) |
| Serbia | 64.09 (53.35-75.46) | 0.71 (0.59-0.83) | 109.02 (89.21-130) | 0.73 (0.6-0.86) | 0.15 (0.12 to 0.17) |
| Seychelles | 0.33 (0.28-0.38) | 0.59 (0.5-0.68) | 0.55 (0.47-0.63) | 0.6 (0.51-0.69) | 0.04 (0.03 to 0.05) |
| Sierra Leone | 7.08 (6.01-8.24) | 0.48 (0.41-0.55) | 12.55 (10.74-14.41) | 0.49 (0.42-0.57) | 0.09 (0.06 to 0.11) |
| Singapore | 9.2 (7.9-10.6) | 0.58 (0.5-0.67) | 45.03 (39.15-51.29) | 0.64 (0.55-0.73) | 0.22 (0.16 to 0.28) |
| Slovakia | 39.39 (32.69-46.38) | 0.73 (0.61-0.86) | 69 (57.74-80.49) | 0.76 (0.64-0.88) | 0.17 (0.15 to 0.18) |
| Slovenia | 17.35 (14.48-20.32) | 0.76 (0.64-0.88) | 40.73 (34.1-47.39) | 0.79 (0.66-0.91) | 0.17 (0.15 to 0.18) |
| Solomon Islands | 0.37 (0.31-0.43) | 0.57 (0.48-0.66) | 0.9 (0.76-1.05) | 0.57 (0.48-0.66) | 0.02 (-0.02 to 0.05) |
| Somalia | 7.87 (6.76-9.07) | 0.57 (0.5-0.66) | 20.44 (17.39-23.75) | 0.58 (0.5-0.67) | 0.07 (0.05 to 0.09) |
| South Africa | 100.03 (85.93-115.11) | 0.6 (0.52-0.69) | 211.85 (182.29-244.28) | 0.61 (0.52-0.7) | 0.05 (-0.01 to 0.1) |
| South Sudan | 9.74 (8.3-11.27) | 0.59 (0.5-0.68) | 14.33 (12.31-16.44) | 0.56 (0.48-0.64) | -0.14 (-0.17 to -0.11) |
| Spain | 392.24 (351.21-431.35) | 0.73 (0.65-0.8) | 761.03 (660.69-875.74) | 0.63 (0.55-0.72) | -0.32 (-0.38 to -0.26) |
| Sri Lanka | 44.95 (38.24-51.81) | 0.57 (0.49-0.66) | 131.43 (112.19-151.33) | 0.61 (0.53-0.7) | 0.25 (0.23 to 0.27) |
| Sudan | 47.45 (40.01-55.55) | 0.74 (0.63-0.86) | 100.53 (85.73-115.96) | 0.75 (0.63-0.86) | -0.02 (-0.05 to 0.02) |
| Suriname | 1.19 (1.03-1.37) | 0.52 (0.44-0.6) | 2.82 (2.43-3.27) | 0.53 (0.45-0.61) | 0.07 (0.05 to 0.1) |
| Sweden | 109.08 (91.97-128.1) | 0.64 (0.54-0.74) | 146.35 (123.66-170.21) | 0.57 (0.49-0.66) | -0.22 (-0.28 to -0.15) |
| Switzerland | 74.38 (63.01-87.02) | 0.65 (0.55-0.75) | 135.92 (114.91-157.37) | 0.64 (0.54-0.74) | -0.02 (-0.03 to -0.02) |
| Syria | 29.31 (25.01-33.8) | 0.75 (0.64-0.87) | 63.68 (53.66-73.95) | 0.77 (0.65-0.89) | 0.09 (0.08 to 0.11) |
| Taiwan | 57.29 (48.57-65.75) | 0.53 (0.45-0.61) | 255.04 (221.37-282.84) | 0.63 (0.54-0.69) | 0.78 (0.64 to 0.92) |
| Tajikistan | 18.4 (15.54-21.31) | 0.69 (0.59-0.8) | 18.07 (15.32-20.91) | 0.7 (0.59-0.82) | 0.05 (0.02 to 0.07) |
| Thailand | 147.3 (126.72-167.71) | 0.56 (0.49-0.64) | 614.5 (533.61-703.36) | 0.62 (0.54-0.72) | 0.51 (0.43 to 0.59) |
| Timor-Leste | 0.96 (0.83-1.11) | 0.57 (0.48-0.66) | 3.43 (2.91-3.96) | 0.57 (0.48-0.65) | 0 (-0.06 to 0.05) |
| Togo | 3.84 (3.27-4.45) | 0.48 (0.41-0.56) | 11.26 (9.68-12.99) | 0.5 (0.43-0.59) | 0.17 (0.14 to 0.2) |
| Tokelau | 0.01 (0.01-0.01) | 0.57 (0.48-0.66) | 0.01 (0.01-0.01) | 0.58 (0.49-0.68) | 0.06 (0.05 to 0.07) |
| Tonga | 0.24 (0.2-0.28) | 0.59 (0.5-0.68) | 0.44 (0.38-0.52) | 0.59 (0.5-0.69) | 0.04 (0.03 to 0.05) |
| Tobago | 3.53 (2.97-4.12) | 0.51 (0.43-0.59) | 9.22 (7.92-10.63) | 0.53 (0.45-0.62) | 0.15 (0.14 to 0.16) |
| Tunisia | 26.84 (22.56-31.31) | 0.78 (0.66-0.9) | 84.64 (72-97.97) | 0.79 (0.67-0.92) | 0.07 (0.06 to 0.09) |
| Turkey | 215.29 (184.01-248.44) | 0.77 (0.65-0.89) | 643.12 (550.26-741.76) | 0.81 (0.69-0.93) | 0.19 (0.17 to 0.2) |
| Turkmenistan | 9.69 (8.22-11.3) | 0.71 (0.6-0.83) | 20.26 (17.27-23.34) | 0.73 (0.62-0.84) | 0.09 (0.07 to 0.1) |
| Tuvalu | 0.03 (0.02-0.03) | 0.59 (0.5-0.69) | 0.04 (0.04-0.05) | 0.58 (0.49-0.67) | -0.11 (-0.15 to -0.08) |
| Uganda | 24.76 (21.17-28.7) | 0.57 (0.49-0.66) | 55.12 (47.7-63.48) | 0.59 (0.5-0.67) | 0.1 (0.08 to 0.11) |
| Ukraine | 421.09 (349.45-501.56) | 0.69 (0.57-0.8) | 561.48 (468.19-660.44) | 0.72 (0.6-0.84) | 0.22 (0.2 to 0.24) |
| United Arab Emirates | 1.1 (0.91-1.29) | 0.72 (0.61-0.85) | 8.09 (6.63-9.65) | 0.72 (0.6-0.84) | -0.04 (-0.06 to -0.03) |
| UK | 549.4 (464.92-643.44) | 0.58 (0.49-0.67) | 834.47 (705.94-974.95) | 0.57 (0.48-0.66) | 0.03 (-0.01 to 0.08) |
| Tanzania | 40.39 (34.63-46.99) | 0.57 (0.49-0.66) | 99.72 (87.49-112.78) | 0.56 (0.49-0.64) | -0.03 (-0.05 to -0.01) |
| USA | 2750.96 (2322.03-3181.76) | 0.8 (0.68-0.92) | 4902.7 (4404.15-5383.45) | 0.78 (0.7-0.86) | 0.11 (0.04 to 0.17) |
| Virgin Islands | 0.33 (0.28-0.38) | 0.52 (0.45-0.61) | 0.91 (0.77-1.05) | 0.54 (0.46-0.62) | 0.12 (0.11 to 0.12) |
| Uruguay | 24.86 (20.82-29.17) | 0.65 (0.55-0.76) | 44.14 (37.21-51.42) | 0.68 (0.57-0.78) | 0.12 (0.12 to 0.13) |
| Uzbekistan | 66.38 (55.97-77.43) | 0.7 (0.59-0.82) | 64.34 (53.97-74.87) | 0.71 (0.6-0.83) | 0.07 (0.04 to 0.09) |
| Vanuatu | 0.22 (0.18-0.26) | 0.55 (0.46-0.64) | 0.63 (0.52-0.74) | 0.55 (0.46-0.64) | -0.01 (-0.04 to 0.02) |
| Venezuela | 43.55 (37.54-50.43) | 0.56 (0.48-0.65) | 147.51 (128.06-167.93) | 0.55 (0.47-0.63) | 0 (-0.07 to 0.06) |
| Vietnam | 196.86 (167.02-226.89) | 0.59 (0.5-0.68) | 458.06 (394.05-526.42) | 0.61 (0.52-0.7) | 0.14 (0.11 to 0.16) |
| Yemen | 22.27 (18.72-26.1) | 0.77 (0.65-0.89) | 64.76 (54.82-74.75) | 0.75 (0.64-0.87) | -0.09 (-0.13 to -0.05) |
| Zambia | 9.68 (8.21-11.15) | 0.54 (0.46-0.62) | 23.94 (20.47-27.46) | 0.57 (0.49-0.65) | 0.19 (0.15 to 0.24) |
| Zimbabwe | 15.7 (13.38-18.24) | 0.6 (0.52-0.7) | 26.13 (22.42-30.21) | 0.59 (0.5-0.68) | -0.11 (-0.17 to -0.06) |

ASPR, age-standardized prevalence rate; EAPC, estimated annual percent change;

**Supplement Table 3.** Death numbers and age-standardized death rates (ASDR) of Alzheimer's disease and other dementias in 1990 and 2019 and the temporal trends from 1990 to 2019 in 204 countries.

|  | **1990** | | **2019** | |  |
| --- | --- | --- | --- | --- | --- |
| **location** | **Deaths cases** | **ASDR** | **Deaths cases** | **ASDR** | **EAPC of ASDR from 1990 to 2019** |
| Afghanistan | 1115.65 (266.16-3089.22) | 31.83 (7.82-84.77) | 1774.91 (422.05-4800.83) | 30.79 (7.46-82.34) | -0.14 (-0.17 to -0.12) |
| Albania | 360.31 (88.52-984.05) | 24.78 (6.1-67.39) | 916.71 (217.37-2373.58) | 24 (5.83-61.02) | -0.13 (-0.15 to -0.1) |
| Algeria | 1336.85 (297.87-3844.78) | 28.78 (6.74-81.6) | 5209.3 (1255.55-14967.61) | 26.97 (6.56-74.54) | -0.21 (-0.22 to -0.2) |
| American Samoa | 2.58 (0.6-7.05) | 24.21 (5.69-66.23) | 7.29 (1.72-19.52) | 23.03 (5.49-60.18) | -0.14 (-0.19 to -0.09) |
| Andorra | 5.55 (1.31-15.75) | 21.23 (5.18-58.45) | 36.35 (9.69-93.46) | 20.3 (5.29-52.94) | -0.11 (-0.15 to -0.07) |
| Angola | 292.96 (65.34-836.23) | 21.39 (4.81-61.11) | 1143.09 (278.4-3088.83) | 27.04 (6.85-72.33) | 0.83 (0.79 to 0.88) |
| Antigua | 12.34 (3.02-33.08) | 20.92 (5.13-55.63) | 16.46 (3.92-44.35) | 21.5 (5.15-57.82) | 0.11 (0.09 to 0.13) |
| Argentina | 4983.22 (1184.35-13857.34) | 20.94 (5.06-56.6) | 12044.64 (2933.15-31989.11) | 21.27 (5.18-56.58) | 0.08 (0.05 to 0.11) |
| Armenia | 359.21 (84.72-1010.07) | 21.2 (5.13-58.35) | 872.95 (206.08-2343.38) | 24.33 (5.82-64.76) | 0.6 (0.53 to 0.67) |
| Australia | 3669.46 (896.14-9852.3) | 22.53 (5.59-59.77) | 11240.24 (2929.19-28399.93) | 22.04 (5.71-55.88) | -0.08 (-0.1 to -0.06) |
| Austria | 2326.85 (564.89-6478.4) | 20.83 (5.17-55.84) | 4801.87 (1203.68-12513.17) | 20.12 (5.06-52.58) | -0.13 (-0.14 to -0.11) |
| Azerbaijan | 900.96 (223-2497.27) | 21.7 (5.37-60.13) | 940.7 (206.59-2726.08) | 25.12 (5.68-69.46) | 0.59 (0.54 to 0.63) |
| Bahamas | 21.9 (5.16-61.22) | 20.93 (5.05-56.38) | 60.91 (15.24-165.08) | 20.78 (5.13-56.31) | -0.04 (-0.07 to 0) |
| Bahrain | 15.52 (3.49-45.12) | 28.54 (6.69-78.86) | 73.71 (17.72-201.75) | 27.29 (6.58-72.03) | -0.07 (-0.14 to -0.01) |
| Bangladesh | 4545.89 (1055-13203.82) | 17.02 (3.96-49.33) | 15614.26 (3797.72-43400.84) | 18.23 (4.45-49.29) | -0.04 (-0.2 to 0.12) |
| Barbados | 58.14 (13.71-158.51) | 19.81 (4.74-53.36) | 100.47 (24.06-266.66) | 20.92 (5.03-55.41) | 0.21 (0.11 to 0.3) |
| Belarus | 2306.24 (541.1-6413.59) | 21.2 (5.1-58.47) | 3685.43 (897.58-9749.2) | 22.16 (5.39-58.39) | 0.16 (0.14 to 0.18) |
| Belgium | 3212.15 (776.06-8634.68) | 22.24 (5.46-58.39) | 6614.55 (1665.61-17204.69) | 20.66 (5.22-54.04) | -0.37 (-0.42 to -0.32) |
| Belize | 17.74 (4.29-47.48) | 21.2 (5.14-56.63) | 47.52 (11.59-123.23) | 21.83 (5.31-55.95) | 0.07 (-0.02 to 0.16) |
| Benin | 327.14 (78.57-901.04) | 24.91 (6.04-67.78) | 695.36 (170.29-1912.3) | 26.09 (6.47-71.02) | 0.01 (-0.05 to 0.06) |
| Bermuda | 10.25 (2.45-28.64) | 21.72 (5.31-59.15) | 31.84 (8.03-81.17) | 21.44 (5.4-54.75) | -0.03 (-0.08 to 0.02) |
| Bhutan | 18.19 (4.18-51.85) | 17.49 (3.94-49.02) | 80.55 (19.27-216.48) | 21.68 (5.25-57.41) | 0.76 (0.7 to 0.81) |
| Bolivia | 404.7 (95.64-1104.37) | 22.62 (5.42-61.22) | 1392.53 (327.31-3780.21) | 24.1 (5.65-64) | 0.2 (0.19 to 0.22) |
| Bosnia and Herzegovina | 508.75 (118.41-1423.63) | 23.65 (5.84-65.26) | 1079.65 (249.99-2939.5) | 22.63 (5.36-60.11) | -0.17 (-0.22 to -0.11) |
| Botswana | 57 (13.32-159.81) | 22.72 (5.36-64.01) | 152.76 (36.52-424.18) | 24.91 (6.13-68.07) | 0.31 (0.28 to 0.34) |
| Brazil | 13314 (3166.04-36721.32) | 26.79 (6.59-71.49) | 54594.03 (13841.8-140985.07) | 25.59 (6.52-66.35) | -0.09 (-0.13 to -0.06) |
| Brunei | 7.35 (1.68-20.88) | 22.44 (5.33-63.31) | 23.54 (5.53-64.69) | 24.35 (5.86-65.76) | 0.33 (0.27 to 0.39) |
| Bulgaria | 1463.64 (334.79-4303.34) | 22.79 (5.36-64.55) | 3146.04 (718.8-8837.79) | 22.75 (5.26-61.92) | -0.1 (-0.16 to -0.05) |
| Burkina Faso | 519.12 (121.25-1455.18) | 27.13 (6.61-77.34) | 1244.7 (301.88-3406.48) | 26.66 (6.46-72) | -0.12 (-0.17 to -0.07) |
| Burundi | 235.35 (56.44-665.34) | 20.47 (4.83-55.27) | 479.09 (113.74-1362.81) | 24.36 (5.9-67.78) | 0.74 (0.64 to 0.83) |
| Cape Verde | 45.09 (11-120.85) | 19.52 (4.78-52.16) | 99.34 (23.8-272.86) | 23.37 (5.6-64.59) | 0.5 (0.43 to 0.57) |
| Cambodia | 488.47 (115.04-1377.63) | 21.64 (5.08-60.68) | 1794.46 (429.83-4851.81) | 26.02 (6.31-68.77) | 0.72 (0.68 to 0.75) |
| Cameroon | 559.21 (132.52-1588.16) | 25.57 (6.16-70.46) | 1504.54 (369.09-4319.01) | 26.24 (6.48-73.11) | 0.08 (0.06 to 0.09) |
| Canada | 6456.44 (1606.31-16949.83) | 21.99 (5.45-56.76) | 17051.84 (4381.82-42584.68) | 20.5 (5.24-51.7) | -0.22 (-0.29 to -0.15) |
| Central African Republic | 88.56 (20.25-246.37) | 22.18 (5.2-61.18) | 160.14 (36.46-459.3) | 22.54 (5.2-63.85) | 0.21 (0.14 to 0.27) |
| Chad | 386.07 (90.19-1122.73) | 22.88 (5.47-66.73) | 715.07 (169.29-1996.57) | 24.43 (5.88-67.3) | 0.21 (0.19 to 0.24) |
| Chile | 1327.51 (317.98-3690.7) | 20.11 (4.9-53.86) | 4803.96 (1215.08-12768.35) | 20.48 (5.19-54.43) | 0.19 (0.14 to 0.25) |
| China | 93315.53 (21008.34-261872.35) | 23.41 (5.4-64.04) | 320714.97 (76156.43-843370.84) | 23.32 (5.66-61.31) | 0.15 (0.07 to 0.23) |
| Colombia | 2677.98 (651.68-7242.73) | 23.13 (5.72-60.74) | 13672.39 (3443.57-34028.86) | 23.09 (5.87-58.67) | 0.04 (-0.01 to 0.08) |
| Comoros | 26.81 (6.2-74.63) | 22.56 (5.27-60.16) | 85.26 (20.92-234.41) | 26.34 (6.62-71.73) | 0.59 (0.54 to 0.65) |
| Republic of Congo | 91.78 (21.04-261.2) | 24.53 (5.63-67.47) | 308.57 (74.3-850.2) | 27.63 (6.61-75.37) | 0.55 (0.48 to 0.62) |
| Cook Islands | 1.95 (0.46-5.22) | 24.64 (5.87-64.85) | 4.48 (1.09-11.78) | 20.98 (5.09-54.1) | -0.5 (-0.58 to -0.42) |
| Costa Rica | 343.73 (85.64-921.75) | 23.55 (5.88-62.98) | 1295.16 (332.44-3181.09) | 24.16 (6.15-59.8) | 0.01 (-0.04 to 0.07) |
| Croatia | 1012.86 (235.45-2836.01) | 23.24 (5.51-64.34) | 2165.52 (510.98-5732.58) | 23.42 (5.54-60.44) | 0.06 (0.03 to 0.08) |
| Cuba | 1746.71 (429.56-4858.41) | 20.56 (5.07-55.54) | 4503.23 (1140.19-11830.44) | 20.58 (5.13-54.53) | 0 (-0.04 to 0.04) |
| Cyprus | 96.13 (22.19-277) | 24.31 (5.77-66.23) | 324.13 (77.99-914.79) | 22.76 (5.6-61.82) | -0.23 (-0.26 to -0.19) |
| Czech Republic | 2430.71 (561.15-6904.37) | 22.17 (5.28-61.92) | 5120.76 (1243.62-13031.12) | 22.85 (5.55-57.93) | 0.14 (0.11 to 0.17) |
| Ivory Coast | 344.88 (79.89-941.12) | 24.92 (6.08-69.48) | 1184.14 (288.02-3297.52) | 25.36 (6.23-69) | 0.08 (0.04 to 0.12) |
| North Korea | 1913.41 (450.57-5174.9) | 23 (5.42-61) | 5207.89 (1271.93-14194.67) | 22.25 (5.42-60.13) | -0.08 (-0.11 to -0.05) |
| Democratic Republic of the Congo | 1223.28 (283.43-3507.66) | 24.16 (5.69-69.53) | 4384.27 (1087.56-11706.61) | 26.45 (6.55-71.16) | 0.33 (0.29 to 0.37) |
| Denmark | 1687.56 (413.1-4529.81) | 19.8 (4.83-52.73) | 2702.83 (685.83-7175.36) | 19.69 (4.96-52.76) | -0.16 (-0.25 to -0.08) |
| Djibouti | 10.24 (2.39-28.27) | 23.6 (5.66-64.61) | 58.3 (14.02-158.96) | 26.93 (6.63-73.97) | 0.54 (0.49 to 0.59) |
| Dominica | 14.84 (3.52-40.61) | 21.43 (5.1-57.12) | 19.24 (4.53-51.68) | 21.35 (5.01-56.71) | 0.03 (-0.02 to 0.09) |
| Dominican Republic | 512.19 (123.6-1398.61) | 21.06 (5.14-56.04) | 1629.08 (385.63-4317.3) | 20.72 (4.92-55.05) | 0.21 (0.12 to 0.3) |
| Ecuador | 797.23 (194.51-2154.89) | 21.98 (5.34-58.94) | 2638.32 (630.09-7427.26) | 22.67 (5.49-62.33) | 0.14 (0.11 to 0.18) |
| Egypt | 3282.06 (755.02-9441.7) | 24.02 (5.66-67.15) | 6918 (1553.87-18509.36) | 23.6 (5.39-63.05) | 0 (-0.07 to 0.07) |
| El Salvador | 590.81 (149.1-1566.11) | 22.42 (5.68-59.96) | 1695.12 (424.04-4238.16) | 23.64 (5.83-59.66) | 0.16 (0.13 to 0.2) |
| Equatorial Guinea | 16.69 (3.68-47.26) | 20.28 (4.71-58.12) | 64.96 (14.89-180.05) | 27.22 (6.57-74.72) | 1.1 (0.93 to 1.26) |
| Eritrea | 50.17 (10.86-142.44) | 17.69 (3.92-52.28) | 248.08 (56.43-701.33) | 26.99 (6.34-75.51) | 1.37 (1.19 to 1.55) |
| Estonia | 330.2 (76.79-921.99) | 20.18 (4.78-56.16) | 756.7 (187.06-2018.44) | 22.89 (5.73-60.42) | 0.4 (0.35 to 0.46) |
| Swaziland | 31.48 (7.39-87.35) | 21.92 (5.23-60.42) | 54.57 (12.74-155.12) | 22.42 (5.32-61.33) | 0.05 (-0.01 to 0.1) |
| Ethiopia | 1494.05 (333.59-4309.31) | 23.16 (5.28-66.28) | 6432.97 (1548.42-17480.71) | 27.82 (6.68-75.12) | 0.76 (0.69 to 0.82) |
| Fiji | 35.98 (8.38-104.24) | 24.36 (5.7-69.78) | 73.32 (17.36-200.14) | 22.68 (5.35-61.77) | -0.22 (-0.25 to -0.2) |
| Finland | 1379.85 (332.76-3709.71) | 21.75 (5.35-57.31) | 3280.17 (832.24-8278.19) | 20.1 (5.13-50.98) | -0.3 (-0.35 to -0.25) |
| France | 18823.01 (4598.25-49242.39) | 21.55 (5.32-55.11) | 46007.88 (11987.56-111471.67) | 21.99 (5.7-53.78) | 0.08 (0.02 to 0.14) |
| Gabon | 82.23 (19.27-220.48) | 27.53 (6.63-73.16) | 166.76 (40.29-452.64) | 28.59 (6.82-74.53) | 0.13 (0.12 to 0.15) |
| Gambia | 37.88 (9.02-107.11) | 24.91 (6.02-68.22) | 154.27 (37.3-433.99) | 26.79 (6.69-74.15) | 0.21 (0.17 to 0.24) |
| Georgia | 848.44 (196.56-2420.77) | 22.26 (5.25-61.95) | 1720.52 (413.66-4569.83) | 22.82 (5.49-61.45) | 0.15 (0.08 to 0.23) |
| Germany | 29984.22 (7269.33-78183.45) | 24.75 (6.05-63.15) | 49556.53 (12294.62-130251.91) | 20.2 (5.03-52.6) | -0.94 (-1.08 to -0.81) |
| Ghana | 616.19 (143.77-1707.98) | 22.57 (5.42-61.74) | 1941.7 (467.96-5296.55) | 23.81 (5.83-63.97) | 0.08 (0.02 to 0.14) |
| Greece | 2519.86 (605.13-7059.18) | 20.6 (5-55.5) | 6996.37 (1725.1-18506.39) | 20.38 (5.01-53.81) | -0.05 (-0.07 to -0.03) |
| Greenland | 3.23 (0.76-8.93) | 22.36 (5.43-60.02) | 8.98 (2.15-23.86) | 22.26 (5.34-58.23) | -0.06 (-0.09 to -0.03) |
| Grenada | 17.82 (4.21-49.2) | 20.12 (4.76-54.81) | 16.26 (3.91-43.53) | 21.29 (5.11-56.21) | 0.26 (0.22 to 0.31) |
| Guam | 7.32 (1.68-20.46) | 24.66 (5.87-66.1) | 38.33 (9.57-98.04) | 20.51 (5.08-52.48) | -0.71 (-0.82 to -0.61) |
| Guatemala | 418.75 (98.81-1202.92) | 24.54 (5.91-71.64) | 1940.93 (461.48-5244.48) | 24.43 (5.86-64.46) | -0.12 (-0.16 to -0.07) |
| Guinea | 465.11 (107.48-1326.5) | 23.67 (5.56-67.39) | 930.68 (228.36-2504.72) | 25.59 (6.29-69.86) | 0.27 (0.23 to 0.32) |
| Guinea-Bissau | 40.38 (9.21-117.67) | 23.73 (5.36-65.28) | 78.75 (18.87-215.98) | 25.57 (6.2-70.61) | 0.23 (0.21 to 0.25) |
| Guyana | 44.77 (10.43-123.71) | 20.53 (4.89-55.78) | 79.24 (18.9-211.73) | 21.05 (5.03-55.17) | -0.01 (-0.11 to 0.08) |
| Haiti | 280.14 (63.23-818.25) | 18.9 (4.31-55.01) | 766.6 (168.97-2163.82) | 21.31 (4.96-59.82) | 0.43 (0.39 to 0.47) |
| Honduras | 334.74 (82.53-920.64) | 23.78 (5.96-64.55) | 1057.97 (255.13-2875.89) | 27.44 (6.74-73.77) | 0.43 (0.36 to 0.5) |
| Hungary | 2390.45 (537.48-6682.6) | 22.27 (5.31-62.11) | 4849.69 (1148.49-12515.69) | 22.76 (5.39-58.37) | 0.09 (0.06 to 0.11) |
| Iceland | 64.95 (15.99-167.7) | 20.79 (5.11-53.83) | 150.66 (39.26-367.91) | 20.33 (5.26-50.46) | -0.09 (-0.13 to -0.06) |
| India | 24194.81 (5436.36-71115.45) | 15.8 (3.58-45) | 129011.25 (31163.78-360200.47) | 19.12 (4.57-52.01) | 0.76 (0.64 to 0.88) |
| Indonesia | 9424.68 (2229.95-26242.3) | 19.15 (4.47-53.22) | 24381.23 (5602.82-67814.48) | 22.46 (5.21-62.43) | 0.57 (0.52 to 0.62) |
| Iran | 2885.79 (673.25-7855.39) | 25.48 (6.04-70.18) | 13910.06 (3443.98-37233.1) | 25.34 (6.35-67.61) | -0.06 (-0.11 to -0.01) |
| Iraq | 1382.31 (333.5-3811.45) | 25.39 (6.25-70.7) | 3258.63 (779.02-8524.4) | 25.27 (6.15-65.74) | -0.06 (-0.07 to -0.05) |
| Ireland | 667 (158.99-1869.78) | 21.12 (5.15-56.84) | 1656.98 (409.13-4253.05) | 20.86 (5.16-53.47) | -0.01 (-0.02 to 0.01) |
| Israel | 728.23 (174.4-2032.14) | 20.2 (5-54.69) | 2776.86 (710.89-7145.69) | 20.2 (5.12-52.58) | -0.02 (-0.04 to -0.01) |
| Italy | 14440.11 (3595.07-38646.32) | 20.37 (5.19-51.94) | 48762.75 (12450.34-127992.56) | 23.75 (6.12-62.3) | 0.78 (0.59 to 0.97) |
| Jamaica | 366.38 (88.92-989.19) | 20.34 (4.97-54.16) | 780.13 (195.17-1970.88) | 20.42 (5.01-51.84) | 0.01 (-0.03 to 0.05) |
| Japan | 28440.26 (6906.59-78264.86) | 21.55 (5.3-57.67) | 164873.93 (46751.63-394127.23) | 27.48 (7.62-66.3) | 1.1 (0.94 to 1.27) |
| Jordan | 168 (39.93-464.62) | 25.97 (6.21-71.25) | 767.04 (188.09-2164.05) | 24 (5.95-61.97) | -0.38 (-0.43 to -0.33) |
| Kazakhstan | 1827.69 (441.98-5091.59) | 21.23 (5.17-58.69) | 2319.36 (531.54-6582.05) | 23.35 (5.62-64.43) | 0.21 (0.14 to 0.27) |
| Kenya | 1013.18 (241.4-2784.65) | 23.6 (5.67-62.92) | 2705.31 (655.76-7435.5) | 26.07 (6.38-70.6) | 0.35 (0.33 to 0.38) |
| Kiribati | 3.9 (0.89-11.24) | 28.92 (6.68-81.1) | 7.77 (1.84-21.29) | 33.29 (7.96-90.73) | 0.51 (0.47 to 0.55) |
| Kuwait | 78.75 (18.97-204.29) | 25.62 (6.26-65.95) | 425.12 (109.97-1085.66) | 25.08 (6.51-64.04) | -0.03 (-0.08 to 0.01) |
| Kyrgyzstan | 565.51 (139.96-1504.28) | 21.17 (5.22-56.13) | 722.74 (172.93-2029.48) | 23.47 (5.71-65.96) | 0.42 (0.3 to 0.54) |
| Laos | 186.29 (42.76-543.65) | 20.02 (4.62-58.23) | 528.85 (129.65-1503.34) | 22.35 (5.57-63.38) | 0.33 (0.31 to 0.34) |
| Latvia | 610.19 (139.79-1730.81) | 19.86 (4.68-54.94) | 1031.05 (246.36-2704.22) | 21.34 (5.12-55.41) | 0.23 (0.15 to 0.3) |
| Lebanon | 362.74 (86.17-980.24) | 27.49 (6.55-73.93) | 1255.24 (305.76-3374.97) | 26.79 (6.55-70.79) | -0.1 (-0.13 to -0.07) |
| Lesotho | 118.76 (28.16-336.82) | 21.56 (5.1-59.94) | 128.99 (30.93-367.38) | 22.42 (5.3-63.37) | 0.15 (0.11 to 0.19) |
| Liberia | 138.82 (32.62-401.93) | 24.25 (5.91-68.31) | 284.27 (69.75-771.15) | 24.99 (6.2-67.84) | 0.15 (0.09 to 0.22) |
| Libya | 417.01 (104.54-1097.36) | 29.67 (7.49-77.95) | 1093.91 (276.28-2788.64) | 28.55 (7.24-72.77) | -0.05 (-0.12 to 0.01) |
| Lithuania | 854.67 (198.71-2327.29) | 20.91 (5-56.61) | 1542.05 (377.83-4096.57) | 21.95 (5.39-57.52) | 0.11 (0.07 to 0.15) |
| Luxembourg | 93.98 (22.4-260.26) | 20.6 (5.04-54.53) | 241.48 (61.49-623.36) | 19.33 (4.88-50.16) | -0.29 (-0.35 to -0.23) |
| Madagascar | 498.73 (116.7-1385.25) | 18.86 (4.47-54.04) | 858.68 (200.57-2361.67) | 19.99 (4.64-55.16) | 0.2 (0.17 to 0.23) |
| Malawi | 366.75 (84.85-1061.01) | 23.51 (5.76-64.49) | 1019.19 (252.36-2756.74) | 26.35 (6.67-70.91) | 0.51 (0.46 to 0.57) |
| Malaysia | 1266.91 (299.64-3446.85) | 21.44 (5.17-57.73) | 3667.45 (855.08-9835.63) | 21.61 (5.15-57.12) | -0.05 (-0.15 to 0.04) |
| Maldives | 7.21 (1.66-21.07) | 20.99 (4.93-59.85) | 47.68 (11.76-122.92) | 22.7 (5.59-58.33) | 0.21 (0.18 to 0.25) |
| Mali | 463.97 (108.41-1275.05) | 24.97 (5.86-67.41) | 1226.26 (290.69-3360.3) | 25.96 (6.17-70.81) | 0.13 (0.09 to 0.17) |
| Malta | 65.02 (15.7-178.81) | 20.49 (5.09-55.46) | 219.63 (54.74-560.17) | 20.38 (5.07-52.2) | -0.05 (-0.08 to -0.03) |
| Marshall Islands | 1.98 (0.46-5.59) | 27.55 (6.35-75.87) | 3.01 (0.68-8.45) | 26.34 (6.3-73.02) | -0.11 (-0.18 to -0.05) |
| Mauritania | 136.49 (31.91-377.51) | 24.74 (5.89-68.23) | 347.22 (84.64-915.9) | 25.34 (6.22-65.5) | 0.06 (0.03 to 0.09) |
| Mauritius | 97.36 (23.21-272.43) | 22.67 (5.48-61.39) | 322.86 (78.9-829.31) | 22.34 (5.46-57.05) | -0.05 (-0.08 to -0.02) |
| Mexico | 6576.7 (1585.4-18338.47) | 23.62 (5.72-64.4) | 23656.85 (5772.81-62437.57) | 24.17 (5.98-63.76) | 0 (-0.06 to 0.07) |
| Micronesia | 5.18 (1.23-14.87) | 25.94 (6.09-72.69) | 7.61 (1.77-21.14) | 26.44 (6.37-70.74) | 0.04 (0.02 to 0.07) |
| Monaco | 16.71 (4.15-44.23) | 19.3 (4.78-50.74) | 28.29 (7.25-72.89) | 20.13 (5.13-51.01) | 0.23 (0.17 to 0.3) |
| Mongolia | 140.14 (33.36-408.54) | 24.49 (5.93-69.9) | 224.54 (52.58-646.61) | 24.1 (5.65-67) | -0.12 (-0.15 to -0.08) |
| Montenegro | 115.12 (27.55-318.57) | 23.38 (5.65-64.21) | 184.54 (42.64-491.37) | 23.95 (5.59-63.17) | 0.08 (0.04 to 0.12) |
| Morocco | 1888.22 (436.64-5270.6) | 25.19 (6.04-70.5) | 4944.85 (1121.68-13767.69) | 25.36 (5.85-70.18) | 0.04 (-0.02 to 0.09) |
| Mozambique | 632.28 (146.49-1778.43) | 25.58 (6.04-70.65) | 1529.33 (364.29-4152.54) | 29.74 (7.29-80.08) | 0.58 (0.55 to 0.61) |
| Myanmar | 2393.12 (565.1-6946.92) | 20.75 (5.05-57.44) | 6784.07 (1632.89-18742.06) | 22.7 (5.53-61.73) | 0.34 (0.27 to 0.4) |
| Namibia | 76.82 (17.74-220.24) | 21.77 (5.08-60.7) | 228.17 (54.97-622.81) | 24.96 (6.15-66.47) | 0.45 (0.42 to 0.48) |
| Nauru | 0.33 (0.07-0.95) | 26.62 (6.16-75.55) | 0.24 (0.05-0.7) | 24.42 (5.85-68.78) | -0.29 (-0.33 to -0.24) |
| Nepal | 631.34 (144.82-1852.04) | 15.09 (3.46-44.37) | 2448.14 (569.87-6844.83) | 19.51 (4.53-53.31) | 0.88 (0.86 to 0.9) |
| Netherlands | 4600.66 (1133.37-11779.37) | 23.73 (6.07-59.86) | 8714.56 (2202.51-21710.77) | 22.05 (5.57-55.18) | -0.34 (-0.4 to -0.28) |
| New Zealand | 770.38 (192.28-2071.5) | 22.76 (5.75-59.81) | 2066.43 (530.77-5360.48) | 22.26 (5.67-58.35) | -0.12 (-0.16 to -0.08) |
| Nicaragua | 244.02 (60.46-646.83) | 22.56 (5.67-58.93) | 706.16 (173.55-1889) | 24.7 (6.19-65.32) | 0.27 (0.19 to 0.34) |
| Niger | 247.19 (58.4-723.61) | 23.17 (5.53-66.84) | 853.53 (206.03-2340.31) | 25.63 (6.08-69.98) | 0.39 (0.35 to 0.43) |
| Nigeria | 4984.11 (1163.94-14603.46) | 21.62 (5.22-63.03) | 12113.4 (2919.45-32319.2) | 25.65 (6.24-67.46) | 0.85 (0.72 to 0.98) |
| Niue | 0.72 (0.17-1.97) | 28.64 (6.81-77.98) | 0.49 (0.12-1.34) | 24.9 (5.99-66.52) | -0.53 (-0.58 to -0.49) |
| North Macedonia | 252 (57.25-718.45) | 22.09 (5.19-63.15) | 436.57 (96.54-1208.91) | 23.98 (5.42-64.52) | 0.26 (0.23 to 0.29) |
| Northern Mariana Islands | 1.3 (0.3-3.63) | 23.96 (5.72-66.78) | 5.35 (1.31-14.42) | 23.24 (5.7-62.19) | -0.04 (-0.07 to 0) |
| Norway | 1706.49 (424.01-4529.32) | 22.55 (5.65-58.91) | 2633.3 (672.19-6620.91) | 20.92 (5.31-53.55) | -0.23 (-0.26 to -0.2) |
| Oman | 74.82 (17.39-209.24) | 29.89 (7.14-78.84) | 162.72 (37.34-446.38) | 28.84 (6.7-81.02) | -0.08 (-0.14 to -0.02) |
| Pakistan | 6928.18 (1567.27-19430.03) | 18.7 (4.28-53.4) | 10959.6 (2633.88-31888.69) | 20.98 (5.01-58.43) | 0.4 (0.36 to 0.44) |
| Palau | 1.04 (0.24-2.96) | 21.09 (5-58.49) | 1.88 (0.45-5.22) | 19.49 (4.61-52.86) | -0.3 (-0.34 to -0.25) |
| Palestine | 164.55 (41.15-447.37) | 27.52 (6.94-74.54) | 325.88 (75.66-925.72) | 25.85 (6.08-72.15) | -0.27 (-0.34 to -0.21) |
| Panama | 290.95 (73.33-778.49) | 22.75 (5.72-60.8) | 1053.72 (269.92-2585.17) | 23.4 (5.95-57.79) | 0.08 (0.04 to 0.12) |
| Papua New Guinea | 146.16 (33.19-398.27) | 20.63 (4.82-56.33) | 401.74 (92.68-1115.65) | 20.76 (4.77-56.96) | -0.01 (-0.03 to 0.01) |
| Paraguay | 429.39 (104.46-1137.69) | 23.6 (5.78-62.4) | 1260.88 (317.69-3160.01) | 24.65 (6.14-62.25) | 0.15 (0.09 to 0.21) |
| Peru | 2013.81 (485.68-5470.12) | 21.19 (5.16-57.77) | 7143.98 (1748.66-18218.91) | 21.13 (5.16-54.18) | 0.04 (0 to 0.07) |
| Philippines | 4051.99 (954.18-11218.46) | 26.73 (6.44-73.11) | 10473.1 (2390.21-29495.91) | 22.66 (5.34-63.62) | -0.7 (-0.89 to -0.51) |
| Poland | 8445.54 (2005.44-23014.25) | 25.52 (6.27-69.23) | 18468.54 (4440.62-49689.07) | 23.89 (5.76-63.79) | -0.32 (-0.36 to -0.28) |
| Portugal | 2022.11 (485.41-5654.81) | 20.24 (4.92-55.41) | 6601.87 (1645.24-17116.36) | 20.54 (5.15-53.13) | 0.09 (0.07 to 0.11) |
| Puerto Rico | 654.88 (159.41-1780.65) | 21.41 (5.21-56.57) | 2026.02 (514.45-5112.16) | 20.95 (5.26-52.89) | -0.08 (-0.11 to -0.06) |
| Qatar | 8.06 (1.83-21.84) | 26.32 (6.22-74.5) | 41.08 (9.67-119.73) | 27.86 (6.48-76.01) | 0.3 (0.25 to 0.35) |
| South Korea | 3364.03 (804.76-9038.49) | 25.72 (6.11-68.11) | 18450.93 (4756.66-47101.55) | 23.94 (6.16-60.27) | -0.36 (-0.47 to -0.24) |
| Moldova | 543.66 (126.84-1546.83) | 22.17 (5.31-61.7) | 1259.25 (307.17-3416.69) | 21.97 (5.37-59.32) | -0.13 (-0.2 to -0.06) |
| Romania | 3709.81 (827.92-10481.35) | 22.19 (5.17-61.94) | 8874.45 (2184.91-23542.45) | 22.07 (5.42-58.22) | -0.06 (-0.07 to -0.04) |
| Russia | 23706.28 (5468.93-65980.69) | 20.37 (4.86-56.08) | 48437.22 (11763.65-129759.6) | 21.17 (5.19-56.11) | 0.14 (0.11 to 0.17) |
| Rwanda | 261.37 (60.79-743.81) | 21.62 (5.05-60.06) | 772.2 (187.38-2128.84) | 27.28 (6.72-75.43) | 1.14 (0.99 to 1.28) |
| Saint Kitts | 5.58 (1.31-15.68) | 21.28 (5.07-59.64) | 8.14 (1.92-22.22) | 20.49 (4.87-55.57) | -0.01 (-0.04 to 0.02) |
| Saint Lucia | 13.08 (3.1-36.91) | 21.03 (4.96-58.01) | 40.89 (9.87-107.94) | 21.72 (5.27-57.1) | 0.05 (0.01 to 0.09) |
| Saint Vincent | 12.03 (2.83-33.71) | 21.92 (5.23-61.25) | 21.88 (5.24-58.7) | 20.58 (4.89-54.72) | -0.17 (-0.2 to -0.14) |
| Samoa | 15.29 (3.65-42.46) | 28.39 (6.82-78.59) | 28.17 (6.77-76.82) | 26.71 (6.43-72.42) | -0.25 (-0.29 to -0.22) |
| San Marino | 6.24 (1.57-16.77) | 21.66 (5.47-57.2) | 19.63 (4.95-50.45) | 20.98 (5.13-54.32) | -0.05 (-0.09 to -0.01) |
| Sao Tome and Principe | 7.73 (1.82-21.95) | 21.52 (5.13-58.66) | 14.23 (3.24-38.28) | 22.86 (5.4-60.52) | 0.21 (0.17 to 0.26) |
| Saudi Arabia | 828.22 (196.69-2255.7) | 26.85 (6.39-72.17) | 1707.46 (411.49-4518.3) | 26.29 (6.31-68.43) | -0.12 (-0.15 to -0.09) |
| Senegal | 436.97 (101.94-1215.36) | 25.28 (5.97-69.78) | 1245.94 (304.16-3352.57) | 27.58 (6.8-73.24) | 0.37 (0.3 to 0.44) |
| Serbia | 1721.83 (392.54-4789.53) | 21.5 (5-59.51) | 2567.35 (570.02-7227.26) | 21.57 (4.86-57.89) | -0.04 (-0.07 to 0) |
| Seychelles | 12.21 (2.88-33.05) | 23.09 (5.47-62.4) | 18.39 (4.52-47.35) | 22.65 (5.55-58.75) | -0.07 (-0.1 to -0.04) |
| Sierra Leone | 259.98 (59.45-749.38) | 22.39 (5.14-65.18) | 484.99 (118.54-1339.33) | 24.32 (5.97-67.01) | 0.35 (0.31 to 0.38) |
| Singapore | 282.56 (69.7-761.78) | 22.36 (5.61-59.45) | 1477.42 (384.9-3645.87) | 21.86 (5.69-53.92) | 0 (-0.09 to 0.09) |
| Slovakia | 1088.47 (258.59-3027.04) | 23.28 (5.63-64.18) | 1959.5 (456.32-4994.04) | 22.8 (5.31-57.47) | -0.02 (-0.05 to 0.01) |
| Slovenia | 479.1 (111.81-1283.13) | 23.63 (5.58-62.61) | 1336.65 (340.15-3406.75) | 23.93 (6-61.26) | 0.08 (0.03 to 0.12) |
| Solomon Islands | 8.04 (1.83-22.95) | 18.02 (4.11-51.97) | 22.11 (5.16-63.15) | 20.47 (4.9-58.05) | 0.39 (0.36 to 0.43) |
| Somalia | 192.29 (43.76-525.81) | 20.27 (4.73-56.02) | 506.19 (115.34-1486.34) | 23.37 (5.51-67.6) | 0.78 (0.68 to 0.88) |
| South Africa | 3168.26 (757.06-8639.31) | 21.62 (5.15-59.04) | 6603.06 (1529.71-18307.23) | 22.55 (5.36-61.8) | 0.11 (0.05 to 0.17) |
| South Sudan | 277.75 (64.09-761.94) | 22.09 (5.22-59.96) | 456.15 (107.78-1284.41) | 22.6 (5.44-63.67) | 0.15 (0.11 to 0.18) |
| Spain | 11872.05 (2904.98-30889.14) | 24.73 (6.16-63.01) | 29207.64 (7446.61-72045.3) | 20.87 (5.32-52.01) | -0.64 (-0.83 to -0.45) |
| Sri Lanka | 1423.08 (332.17-3984.99) | 23.8 (5.64-66.27) | 4140.85 (977.39-10874.13) | 23.53 (5.52-60.92) | 0.12 (0.05 to 0.19) |
| Sudan | 1210.54 (283.83-3477.74) | 25.13 (5.91-70.71) | 2814.81 (662.56-7999.78) | 23.86 (5.62-66.01) | -0.21 (-0.24 to -0.17) |
| Suriname | 47.76 (11.43-129.01) | 21.05 (5.02-57.58) | 109.14 (26.7-279.7) | 21.67 (5.32-54.99) | 0.04 (-0.03 to 0.11) |
| Sweden | 3732.63 (921.54-9937.13) | 22.62 (5.66-59.26) | 5637.41 (1413.76-14630.38) | 19.68 (4.89-51.83) | -0.49 (-0.55 to -0.42) |
| Switzerland | 2289.74 (568.38-6180.36) | 20.47 (5.14-54.65) | 5138.32 (1337.46-12863.36) | 21.07 (5.45-53.52) | 0.05 (-0.02 to 0.11) |
| Syria | 909.62 (214.53-2496.5) | 25.18 (5.95-69.32) | 1591.56 (368.14-4596.05) | 25.95 (6.26-70.29) | 0.08 (0.05 to 0.12) |
| Taiwan | 1627.34 (380.05-4455.85) | 19.61 (4.74-52.45) | 8754.97 (2137.17-22700.57) | 20.84 (5.1-53.76) | 0.36 (0.27 to 0.45) |
| Tajikistan | 591.9 (148.51-1549.18) | 21.53 (5.37-56.77) | 445.65 (103.75-1236.41) | 24.41 (5.83-67.75) | 0.42 (0.37 to 0.48) |
| Thailand | 5158.47 (1259.34-14135.9) | 24.47 (6.1-67.46) | 21552.73 (5396.02-54549.38) | 22.48 (5.65-56.76) | -0.39 (-0.46 to -0.32) |
| Timor-Leste | 27.73 (6.61-79.38) | 20.11 (4.77-57.01) | 101.89 (24.46-287.55) | 22.5 (5.39-63.08) | 0.42 (0.36 to 0.49) |
| Togo | 143.89 (32.88-409.53) | 24.93 (5.88-72.06) | 399.96 (97.31-1068.69) | 25.6 (6.36-69.1) | 0.08 (0.07 to 0.1) |
| Tokelau | 0.3 (0.07-0.83) | 27.79 (6.65-75.73) | 0.27 (0.06-0.72) | 24.66 (5.79-66.47) | -0.41 (-0.43 to -0.39) |
| Tonga | 8.62 (2.07-23.27) | 25.1 (6.11-65.3) | 18.05 (4.36-47.21) | 24.51 (5.92-64.04) | -0.04 (-0.08 to -0.01) |
| Tobago | 110.36 (25.64-311.02) | 20.41 (4.79-55.47) | 335.09 (81.49-882.91) | 20.19 (4.88-53.43) | -0.04 (-0.07 to -0.01) |
| Tunisia | 688.22 (161.5-1924.88) | 27.31 (6.63-74.46) | 2546.82 (596.99-6753.41) | 26.53 (6.32-70.28) | -0.12 (-0.14 to -0.11) |
| Turkey | 6788.4 (1654.1-17869.75) | 26.81 (6.6-70.04) | 19721.31 (5069.67-53643.16) | 25.79 (6.62-70.04) | -0.06 (-0.15 to 0.02) |
| Turkmenistan | 267.16 (63.93-739.94) | 23.35 (5.64-64.28) | 582.18 (143.1-1538.21) | 23.06 (5.65-61.44) | -0.12 (-0.15 to -0.08) |
| Tuvalu | 0.85 (0.2-2.34) | 27.54 (6.42-75.04) | 1.57 (0.37-4.43) | 25.53 (6.21-73.04) | -0.28 (-0.31 to -0.25) |
| Uganda | 680.7 (155.86-1897.75) | 22.27 (5.21-59.45) | 1854.44 (451.46-4943.63) | 25.74 (6.29-67.57) | 0.54 (0.5 to 0.59) |
| Ukraine | 10011.03 (2256.03-28257.59) | 19.84 (4.66-55.07) | 16370.36 (3886.61-45455.31) | 22.47 (5.31-61.85) | 0.42 (0.35 to 0.49) |
| United Arab Emirates | 23.17 (5.24-66.01) | 25.75 (6.02-72.25) | 138.19 (31.42-389.93) | 24.19 (5.86-64.25) | -0.27 (-0.35 to -0.19) |
| UK | 18423.33 (4477.16-50496.22) | 21.08 (5.16-55.99) | 32424.86 (8094.94-83575.29) | 20.77 (5.17-53.66) | -0.04 (-0.06 to -0.03) |
| Tanzania | 1179.55 (272.84-3306.53) | 25.06 (6.14-68.92) | 3644.44 (888.19-9833.42) | 25.76 (6.46-68.87) | 0.14 (0.11 to 0.17) |
| USA | 73079.18 (18399.56-194708.29) | 21.33 (5.4-56.35) | 143918.7 (37067.1-354959.38) | 20.92 (5.35-52.24) | -0.26 (-0.36 to -0.16) |
| Virgin Islands | 10.87 (2.6-30.59) | 22.39 (5.44-60.91) | 29.4 (7.14-77.35) | 21.38 (5.18-55.17) | -0.14 (-0.17 to -0.11) |
| Uruguay | 734.97 (180.66-2018.07) | 21.26 (5.23-57.09) | 1496.81 (371.6-3923.18) | 20.91 (5.18-54.89) | -0.01 (-0.04 to 0.03) |
| Uzbekistan | 1918.76 (473.83-5239.21) | 21.71 (5.34-58.88) | 1118.81 (264.18-3220.8) | 23.73 (5.6-66.88) | 0.35 (0.29 to 0.4) |
| Vanuatu | 6.6 (1.53-19.32) | 22.85 (5.33-68.36) | 19.25 (4.42-55.75) | 23.25 (5.36-63.77) | -0.02 (-0.08 to 0.04) |
| Venezuela | 1628.83 (400.42-4366.55) | 24.03 (5.94-64.19) | 6312.73 (1579.42-15794.73) | 23.81 (5.94-59.78) | -0.04 (-0.08 to 0) |
| Vietnam | 7764.59 (1873.44-20493.57) | 26.41 (6.35-69.08) | 19460.36 (4766.15-50845.09) | 29.21 (7.24-74.6) | 0.42 (0.38 to 0.46) |
| Yemen | 527.81 (124.34-1479.64) | 25.3 (6.14-68.98) | 1730.64 (402.63-4749.64) | 25.48 (6.24-71.05) | 0.02 (-0.01 to 0.04) |
| Zambia | 284.2 (65.63-792.93) | 23.12 (5.33-63.86) | 763.14 (183.57-2046.76) | 25.39 (6.26-67.97) | 0.45 (0.38 to 0.51) |
| Zimbabwe | 456.69 (105.42-1252.25) | 24.67 (5.75-66.37) | 811.59 (194.03-2254.66) | 25.01 (6.09-69.95) | 0.12 (0.06 to 0.17) |

ASDR, age-standardized deaths rate; EAPC, estimated annual percent change;

**Table 4.** DALYs and age-standardized rate of DALYs of Alzheimer's disease and other dementias in 1990 and 2019 and the temporal trends from 1990 to 2019 in 204 countries.

|  | **1990** | | **2019** | |  |
| --- | --- | --- | --- | --- | --- |
| **location** | **DALYs cases (*10^3)** | **ASR_DALYs (*10^3)** | **DALYs cases (*10^3)** | **ASR_DALYs (*10^3)** | **EAPC of ASR_DALYs from 1990 to 2019** |
| Afghanistan | 20.27 (8.18-47.97) | 0.45 (0.18-1.05) | 30.94 (12.73-73.8) | 0.43 (0.18-1) | -0.16 (-0.19 to -0.13) |
| Albania | 5.8 (2.56-12.73) | 0.37 (0.17-0.82) | 14.92 (6.83-32.07) | 0.36 (0.17-0.78) | -0.06 (-0.08 to -0.05) |
| Algeria | 29.06 (12.21-68.81) | 0.42 (0.18-0.99) | 95.57 (41.57-217.27) | 0.4 (0.18-0.91) | -0.18 (-0.19 to -0.16) |
| American Samoa | 0.05 (0.02-0.11) | 0.35 (0.14-0.81) | 0.12 (0.05-0.27) | 0.33 (0.14-0.75) | -0.14 (-0.18 to -0.1) |
| Andorra | 0.11 (0.05-0.25) | 0.3 (0.14-0.69) | 0.47 (0.22-1.01) | 0.29 (0.14-0.63) | -0.1 (-0.13 to -0.06) |
| Angola | 6.19 (2.77-14.42) | 0.33 (0.15-0.75) | 21.63 (9.28-49.22) | 0.38 (0.17-0.87) | 0.56 (0.54 to 0.59) |
| Antigua | 0.18 (0.07-0.41) | 0.3 (0.12-0.69) | 0.26 (0.11-0.58) | 0.31 (0.13-0.7) | 0.1 (0.08 to 0.11) |
| Argentina | 81.93 (37.39-181.65) | 0.31 (0.14-0.67) | 177.44 (80.93-389.21) | 0.31 (0.14-0.69) | 0.13 (0.11 to 0.15) |
| Armenia | 6.18 (2.77-13.63) | 0.32 (0.15-0.71) | 13.79 (6.17-30.18) | 0.36 (0.16-0.78) | 0.44 (0.4 to 0.49) |
| Australia | 58.21 (26.48-128.3) | 0.33 (0.15-0.73) | 154.29 (70.79-327.98) | 0.32 (0.15-0.68) | -0.09 (-0.1 to -0.07) |
| Austria | 36.6 (16.89-81.06) | 0.3 (0.14-0.67) | 64.77 (29.67-138.05) | 0.29 (0.13-0.62) | -0.09 (-0.09 to -0.08) |
| Azerbaijan | 13.68 (6.28-30.04) | 0.33 (0.15-0.73) | 18.27 (8.01-41.11) | 0.36 (0.16-0.83) | 0.37 (0.34 to 0.4) |
| Bahamas | 0.36 (0.15-0.82) | 0.3 (0.12-0.7) | 0.94 (0.4-2.17) | 0.3 (0.13-0.69) | -0.02 (-0.04 to 0) |
| Bahrain | 0.34 (0.14-0.81) | 0.42 (0.17-0.97) | 1.69 (0.73-3.91) | 0.4 (0.18-0.89) | -0.08 (-0.13 to -0.02) |
| Bangladesh | 81.77 (35.22-190.7) | 0.25 (0.11-0.6) | 265.39 (115.18-601.43) | 0.26 (0.11-0.59) | -0.05 (-0.14 to 0.04) |
| Barbados | 0.91 (0.38-2.13) | 0.29 (0.12-0.66) | 1.5 (0.63-3.36) | 0.3 (0.13-0.68) | 0.17 (0.1 to 0.23) |
| Belarus | 37.66 (17.47-83.15) | 0.32 (0.15-0.71) | 56.17 (25.84-123.45) | 0.34 (0.15-0.74) | 0.16 (0.14 to 0.17) |
| Belgium | 49.81 (22.74-110.25) | 0.32 (0.14-0.71) | 87.39 (40.01-189.39) | 0.3 (0.14-0.64) | -0.35 (-0.39 to -0.31) |
| Belize | 0.26 (0.11-0.6) | 0.31 (0.13-0.7) | 0.71 (0.3-1.58) | 0.31 (0.13-0.71) | 0.07 (0.01 to 0.13) |
| Benin | 4.96 (1.94-11.81) | 0.33 (0.13-0.79) | 10.75 (4.16-25.76) | 0.34 (0.13-0.8) | -0.02 (-0.07 to 0.03) |
| Bermuda | 0.17 (0.07-0.38) | 0.31 (0.13-0.72) | 0.45 (0.2-0.98) | 0.31 (0.14-0.68) | -0.01 (-0.05 to 0.03) |
| Bhutan | 0.34 (0.14-0.81) | 0.25 (0.1-0.59) | 1.25 (0.52-2.92) | 0.29 (0.12-0.67) | 0.58 (0.54 to 0.61) |
| Bolivia | 6.92 (2.79-16.13) | 0.32 (0.13-0.73) | 22.54 (9.07-51.22) | 0.34 (0.14-0.77) | 0.2 (0.19 to 0.21) |
| Bosnia and Herzegovina | 9.29 (4.19-20.45) | 0.35 (0.16-0.78) | 18.31 (8.33-39.41) | 0.34 (0.15-0.72) | -0.13 (-0.16 to -0.1) |
| Botswana | 1.08 (0.47-2.49) | 0.33 (0.14-0.75) | 2.72 (1.15-6.36) | 0.35 (0.15-0.8) | 0.21 (0.19 to 0.23) |
| Brazil | 235.36 (101.39-536.58) | 0.39 (0.16-0.88) | 855.33 (377.33-1874.37) | 0.39 (0.17-0.86) | 0.11 (0.07 to 0.14) |
| Brunei | 0.15 (0.06-0.35) | 0.32 (0.14-0.73) | 0.48 (0.2-1.08) | 0.34 (0.15-0.78) | 0.25 (0.19 to 0.31) |
| Bulgaria | 29.39 (13.53-65.98) | 0.34 (0.15-0.78) | 51.71 (23.39-112.55) | 0.34 (0.16-0.74) | -0.06 (-0.11 to -0.02) |
| Burkina Faso | 9.2 (3.55-22.48) | 0.36 (0.14-0.87) | 20.06 (7.97-46.45) | 0.35 (0.14-0.83) | -0.13 (-0.2 to -0.07) |
| Burundi | 4.42 (1.96-10.31) | 0.3 (0.13-0.69) | 8.37 (3.55-19.68) | 0.34 (0.14-0.8) | 0.52 (0.45 to 0.59) |
| Cape Verde | 0.68 (0.29-1.56) | 0.28 (0.12-0.64) | 1.34 (0.54-3.12) | 0.32 (0.13-0.76) | 0.37 (0.33 to 0.42) |
| Cambodia | 9.36 (3.83-22.43) | 0.33 (0.13-0.78) | 31.11 (12.4-72.61) | 0.37 (0.15-0.87) | 0.51 (0.48 to 0.54) |
| Cameroon | 9.08 (3.45-21.71) | 0.34 (0.13-0.82) | 24.51 (9.48-59.4) | 0.34 (0.13-0.85) | 0.08 (0.07 to 0.09) |
| Canada | 102.37 (47.58-218.52) | 0.33 (0.15-0.7) | 243.27 (114.63-505.2) | 0.31 (0.15-0.65) | -0.2 (-0.26 to -0.15) |
| Central African Republic | 1.87 (0.83-4.22) | 0.34 (0.15-0.76) | 3.43 (1.53-8.02) | 0.35 (0.15-0.79) | 0.16 (0.12 to 0.2) |
| Chad | 6.4 (2.56-15.79) | 0.32 (0.12-0.76) | 11.68 (4.66-27.63) | 0.33 (0.13-0.78) | 0.13 (0.11 to 0.14) |
| Chile | 22.87 (10.24-50.24) | 0.3 (0.13-0.66) | 72.83 (33.65-154.96) | 0.31 (0.14-0.65) | 0.19 (0.16 to 0.22) |
| China | 1845.99 (762.07-4320.54) | 0.35 (0.14-0.8) | 5977.04 (2678.98-13100.56) | 0.37 (0.17-0.8) | 0.26 (0.21 to 0.31) |
| Colombia | 42.9 (17.63-99.74) | 0.33 (0.14-0.75) | 186.82 (80.43-406.35) | 0.33 (0.14-0.73) | 0.07 (0.04 to 0.1) |
| Comoros | 0.47 (0.2-1.11) | 0.32 (0.13-0.73) | 1.32 (0.54-3.08) | 0.36 (0.15-0.83) | 0.46 (0.43 to 0.5) |
| Republic of Congo | 1.95 (0.84-4.56) | 0.36 (0.15-0.81) | 5.68 (2.43-13.07) | 0.39 (0.17-0.9) | 0.47 (0.42 to 0.53) |
| Cook Islands | 0.03 (0.01-0.08) | 0.35 (0.14-0.81) | 0.07 (0.03-0.16) | 0.31 (0.13-0.68) | -0.42 (-0.49 to -0.35) |
| Costa Rica | 5.14 (2.12-11.61) | 0.33 (0.14-0.76) | 17.59 (7.48-38) | 0.34 (0.15-0.74) | 0.04 (0.01 to 0.07) |
| Croatia | 18.02 (8.15-40.51) | 0.35 (0.16-0.79) | 34.11 (15.67-73.22) | 0.35 (0.16-0.76) | 0.05 (0.03 to 0.07) |
| Cuba | 27.78 (11.6-63.93) | 0.29 (0.12-0.67) | 61.34 (26.43-138.08) | 0.3 (0.13-0.67) | 0.01 (-0.02 to 0.04) |
| Cyprus | 1.89 (0.8-4.38) | 0.34 (0.14-0.8) | 5.38 (2.4-12.11) | 0.32 (0.14-0.71) | -0.23 (-0.26 to -0.21) |
| Czech Republic | 41.82 (18.87-95.14) | 0.34 (0.15-0.75) | 78.58 (36.66-166.69) | 0.35 (0.16-0.74) | 0.15 (0.12 to 0.18) |
| Ivory Coast | 6.23 (2.42-14.63) | 0.33 (0.13-0.77) | 19.54 (7.61-46.92) | 0.33 (0.13-0.79) | 0.06 (0.03 to 0.09) |
| North Korea | 36.46 (15.01-85.07) | 0.36 (0.15-0.81) | 91.87 (39.16-208.39) | 0.34 (0.15-0.77) | -0.13 (-0.16 to -0.1) |
| Democratic Republic of the Congo | 26.11 (11.2-61.13) | 0.35 (0.15-0.81) | 77.79 (33.59-177.58) | 0.38 (0.16-0.85) | 0.3 (0.28 to 0.33) |
| Denmark | 25.84 (11.81-56.42) | 0.29 (0.13-0.64) | 37.28 (16.94-80.24) | 0.28 (0.13-0.61) | -0.23 (-0.29 to -0.18) |
| Djibouti | 0.2 (0.09-0.48) | 0.33 (0.14-0.75) | 1.07 (0.44-2.53) | 0.36 (0.15-0.86) | 0.38 (0.35 to 0.42) |
| Dominica | 0.23 (0.1-0.54) | 0.31 (0.13-0.7) | 0.28 (0.12-0.64) | 0.3 (0.13-0.69) | -0.01 (-0.05 to 0.02) |
| Dominican Republic | 8.52 (3.5-19.69) | 0.3 (0.13-0.69) | 25.02 (10.51-56.84) | 0.3 (0.13-0.69) | 0.17 (0.11 to 0.22) |
| Ecuador | 13.06 (5.43-29.78) | 0.31 (0.13-0.71) | 41.34 (17.57-98.1) | 0.32 (0.13-0.74) | 0.13 (0.1 to 0.15) |
| Egypt | 63.85 (28.02-146.9) | 0.36 (0.16-0.82) | 135.57 (59.54-305.48) | 0.36 (0.16-0.79) | 0.07 (0.03 to 0.11) |
| El Salvador | 8.66 (3.58-19.44) | 0.33 (0.14-0.74) | 22.2 (9.36-47.35) | 0.34 (0.14-0.73) | 0.09 (0.07 to 0.12) |
| Equatorial Guinea | 0.34 (0.15-0.77) | 0.31 (0.14-0.71) | 1.12 (0.48-2.51) | 0.38 (0.16-0.88) | 0.74 (0.64 to 0.84) |
| Eritrea | 1.14 (0.52-2.57) | 0.28 (0.12-0.63) | 4.67 (1.91-11.06) | 0.37 (0.15-0.87) | 0.94 (0.83 to 1.05) |
| Estonia | 5.68 (2.63-12.57) | 0.31 (0.14-0.68) | 11.22 (5.2-24.37) | 0.35 (0.16-0.75) | 0.4 (0.37 to 0.43) |
| Swaziland | 0.56 (0.24-1.31) | 0.32 (0.14-0.74) | 1.04 (0.46-2.42) | 0.32 (0.13-0.74) | 0.02 (-0.03 to 0.08) |
| Ethiopia | 31.78 (13.17-75.16) | 0.33 (0.14-0.81) | 103.24 (41.35-244.88) | 0.38 (0.15-0.89) | 0.49 (0.45 to 0.53) |
| Fiji | 0.68 (0.27-1.62) | 0.35 (0.14-0.84) | 1.4 (0.58-3.32) | 0.32 (0.13-0.74) | -0.2 (-0.23 to -0.17) |
| Finland | 21.47 (9.64-46.99) | 0.31 (0.14-0.69) | 44.05 (20.33-94.21) | 0.29 (0.13-0.62) | -0.25 (-0.29 to -0.21) |
| France | 274.24 (120.35-610.52) | 0.3 (0.13-0.68) | 582.92 (265.28-1212.61) | 0.32 (0.14-0.66) | 0.13 (0.09 to 0.17) |
| Gabon | 1.43 (0.6-3.26) | 0.39 (0.16-0.89) | 2.74 (1.14-6.2) | 0.4 (0.17-0.91) | 0.1 (0.09 to 0.12) |
| Gambia | 0.66 (0.27-1.55) | 0.33 (0.13-0.78) | 2.4 (0.94-5.79) | 0.35 (0.14-0.83) | 0.18 (0.14 to 0.23) |
| Georgia | 15.52 (7.15-34.14) | 0.34 (0.15-0.76) | 24.13 (10.94-52.86) | 0.34 (0.15-0.75) | 0.09 (0.03 to 0.14) |
| Germany | 441.17 (196.98-957.64) | 0.34 (0.15-0.74) | 710.22 (333.6-1507.62) | 0.3 (0.14-0.64) | -0.58 (-0.66 to -0.5) |
| Ghana | 10.93 (4.33-25.74) | 0.31 (0.12-0.73) | 32.21 (12.78-75.71) | 0.32 (0.13-0.76) | 0.06 (0.02 to 0.09) |
| Greece | 41.84 (19.07-92.37) | 0.3 (0.13-0.67) | 95.47 (43.11-206.42) | 0.3 (0.13-0.65) | -0.03 (-0.04 to -0.02) |
| Greenland | 0.06 (0.03-0.14) | 0.33 (0.15-0.73) | 0.15 (0.07-0.34) | 0.33 (0.15-0.7) | -0.06 (-0.08 to -0.04) |
| Grenada | 0.25 (0.1-0.58) | 0.29 (0.12-0.67) | 0.26 (0.11-0.59) | 0.3 (0.13-0.69) | 0.19 (0.16 to 0.22) |
| Guam | 0.15 (0.06-0.35) | 0.36 (0.14-0.83) | 0.57 (0.25-1.23) | 0.31 (0.14-0.68) | -0.5 (-0.58 to -0.43) |
| Guatemala | 7.45 (2.97-17.65) | 0.34 (0.14-0.83) | 31.25 (12.78-71.84) | 0.34 (0.14-0.79) | -0.06 (-0.09 to -0.03) |
| Guinea | 7.54 (2.96-17.99) | 0.32 (0.13-0.77) | 13.73 (5.37-32.37) | 0.34 (0.13-0.79) | 0.18 (0.15 to 0.21) |
| Guinea-Bissau | 0.74 (0.29-1.78) | 0.32 (0.13-0.79) | 1.36 (0.54-3.3) | 0.34 (0.13-0.82) | 0.17 (0.16 to 0.19) |
| Guyana | 0.75 (0.31-1.74) | 0.29 (0.12-0.66) | 1.29 (0.54-2.99) | 0.3 (0.12-0.67) | 0.02 (-0.04 to 0.09) |
| Haiti | 5.42 (2.28-12.74) | 0.28 (0.12-0.67) | 13.39 (5.49-33.22) | 0.3 (0.12-0.72) | 0.28 (0.26 to 0.3) |
| Honduras | 5.34 (2.18-12.44) | 0.34 (0.14-0.79) | 17.03 (6.66-40.67) | 0.38 (0.15-0.89) | 0.33 (0.29 to 0.38) |
| Hungary | 41.87 (19.1-93.26) | 0.33 (0.15-0.74) | 73.98 (34.17-154.75) | 0.35 (0.16-0.73) | 0.17 (0.15 to 0.19) |
| Iceland | 0.93 (0.42-2.06) | 0.3 (0.14-0.66) | 1.97 (0.9-4.08) | 0.3 (0.14-0.6) | -0.1 (-0.12 to -0.08) |
| India | 520.52 (215.2-1248.01) | 0.23 (0.09-0.55) | 2125.54 (852.82-5117.4) | 0.26 (0.1-0.62) | 0.53 (0.44 to 0.63) |
| Indonesia | 178.58 (75.8-414.14) | 0.29 (0.12-0.67) | 441.93 (180.28-1024.96) | 0.32 (0.13-0.75) | 0.35 (0.32 to 0.39) |
| Iran | 55.07 (23.81-124.61) | 0.39 (0.17-0.88) | 228.13 (100.79-497.82) | 0.39 (0.17-0.84) | -0.04 (-0.07 to -0.01) |
| Iraq | 22.37 (9.79-51.46) | 0.37 (0.16-0.86) | 57.5 (25.21-128.18) | 0.38 (0.17-0.83) | 0.04 (0.03 to 0.05) |
| Ireland | 11.07 (5.02-24.71) | 0.3 (0.14-0.68) | 23.64 (10.76-50.63) | 0.3 (0.14-0.64) | -0.02 (-0.03 to -0.01) |
| Israel | 12.5 (5.71-27.64) | 0.29 (0.13-0.65) | 38.34 (17.59-82.43) | 0.29 (0.14-0.63) | 0 (-0.01 to 0.01) |
| Italy | 247.55 (123.33-509.06) | 0.3 (0.15-0.63) | 669.31 (306.3-1420.82) | 0.35 (0.16-0.75) | 0.72 (0.57 to 0.86) |
| Jamaica | 5.4 (2.3-12.47) | 0.29 (0.12-0.67) | 10.04 (4.23-21.98) | 0.3 (0.13-0.66) | 0.05 (0.01 to 0.08) |
| Japan | 471.38 (208.77-1032.31) | 0.32 (0.14-0.7) | 2060.94 (958.04-4206.26) | 0.39 (0.18-0.8) | 0.93 (0.81 to 1.05) |
| Jordan | 3.06 (1.3-6.99) | 0.39 (0.17-0.87) | 14.83 (6.6-32.94) | 0.37 (0.17-0.82) | -0.23 (-0.27 to -0.19) |
| Kazakhstan | 30.43 (13.89-67.47) | 0.32 (0.15-0.71) | 40.24 (18.34-90.15) | 0.34 (0.15-0.77) | 0.14 (0.1 to 0.19) |
| Kenya | 17.3 (7.17-40.18) | 0.33 (0.13-0.76) | 45.45 (18.71-105.92) | 0.35 (0.14-0.82) | 0.29 (0.26 to 0.31) |
| Kiribati | 0.08 (0.03-0.2) | 0.41 (0.15-1.03) | 0.15 (0.05-0.36) | 0.45 (0.17-1.08) | 0.32 (0.27 to 0.36) |
| Kuwait | 1.41 (0.61-3.08) | 0.4 (0.18-0.87) | 6.86 (3.08-14.69) | 0.39 (0.17-0.84) | -0.04 (-0.07 to -0.01) |
| Kyrgyzstan | 8.8 (4.04-19.17) | 0.32 (0.15-0.7) | 11.64 (5.34-25.92) | 0.35 (0.16-0.77) | 0.35 (0.28 to 0.41) |
| Laos | 3.88 (1.61-9.09) | 0.31 (0.13-0.74) | 9.4 (3.9-22.43) | 0.33 (0.14-0.78) | 0.16 (0.13 to 0.18) |
| Latvia | 10.19 (4.71-22.58) | 0.3 (0.14-0.68) | 15.6 (7.25-33.59) | 0.32 (0.15-0.7) | 0.22 (0.17 to 0.26) |
| Lebanon | 6.39 (2.72-14.67) | 0.41 (0.17-0.9) | 19.73 (8.59-43.39) | 0.4 (0.17-0.87) | -0.1 (-0.12 to -0.08) |
| Lesotho | 2.07 (0.92-4.89) | 0.31 (0.14-0.74) | 2.38 (1.02-5.5) | 0.32 (0.14-0.74) | 0.07 (0.03 to 0.11) |
| Liberia | 2.37 (0.94-5.92) | 0.32 (0.13-0.8) | 4.33 (1.72-10.48) | 0.33 (0.13-0.78) | 0.1 (0.05 to 0.15) |
| Libya | 6.35 (2.66-14.09) | 0.43 (0.18-0.97) | 16.6 (7.06-37.24) | 0.41 (0.18-0.93) | -0.11 (-0.16 to -0.07) |
| Lithuania | 13.93 (6.45-30.47) | 0.32 (0.15-0.7) | 23.16 (10.66-50.06) | 0.33 (0.15-0.71) | 0.09 (0.07 to 0.12) |
| Luxembourg | 1.54 (0.69-3.42) | 0.3 (0.13-0.66) | 3.18 (1.45-6.73) | 0.27 (0.12-0.58) | -0.37 (-0.41 to -0.32) |
| Madagascar | 8.96 (3.97-20.32) | 0.27 (0.12-0.62) | 16.15 (7.06-36.88) | 0.29 (0.12-0.65) | 0.17 (0.15 to 0.19) |
| Malawi | 6.83 (2.87-16.17) | 0.33 (0.14-0.78) | 16.4 (6.8-37.71) | 0.36 (0.15-0.83) | 0.4 (0.36 to 0.44) |
| Malaysia | 22.04 (9.2-51.16) | 0.32 (0.14-0.74) | 65.46 (27.41-151.6) | 0.33 (0.14-0.75) | -0.03 (-0.09 to 0.02) |
| Maldives | 0.16 (0.06-0.37) | 0.32 (0.13-0.76) | 0.78 (0.33-1.75) | 0.34 (0.15-0.75) | 0.18 (0.16 to 0.21) |
| Mali | 8.14 (3.18-19.37) | 0.34 (0.13-0.8) | 19.61 (7.67-47.42) | 0.35 (0.13-0.82) | 0.12 (0.09 to 0.14) |
| Malta | 1.09 (0.5-2.43) | 0.3 (0.13-0.67) | 3.11 (1.43-6.56) | 0.3 (0.14-0.63) | -0.03 (-0.05 to -0.01) |
| Marshall Islands | 0.04 (0.01-0.09) | 0.39 (0.15-0.94) | 0.06 (0.02-0.15) | 0.37 (0.14-0.88) | -0.18 (-0.24 to -0.11) |
| Mauritania | 2.29 (0.89-5.44) | 0.33 (0.13-0.79) | 5.18 (2.05-12.28) | 0.34 (0.13-0.78) | 0.02 (0 to 0.05) |
| Mauritius | 1.73 (0.72-3.97) | 0.33 (0.14-0.78) | 5.06 (2.14-11.38) | 0.33 (0.14-0.74) | -0.02 (-0.04 to -0.01) |
| Mexico | 105.96 (42.71-247.16) | 0.33 (0.13-0.77) | 340.14 (136.5-793.37) | 0.33 (0.13-0.76) | -0.01 (-0.06 to 0.04) |
| Micronesia | 0.1 (0.04-0.25) | 0.37 (0.14-0.9) | 0.14 (0.05-0.34) | 0.37 (0.15-0.88) | -0.02 (-0.05 to 0.02) |
| Monaco | 0.25 (0.11-0.52) | 0.28 (0.13-0.61) | 0.36 (0.16-0.75) | 0.29 (0.13-0.6) | 0.07 (0.04 to 0.1) |
| Mongolia | 2.49 (1.1-5.65) | 0.35 (0.16-0.81) | 4.32 (1.92-9.64) | 0.35 (0.16-0.79) | -0.09 (-0.11 to -0.06) |
| Montenegro | 1.87 (0.85-4.1) | 0.35 (0.16-0.77) | 3.13 (1.41-6.82) | 0.36 (0.16-0.77) | 0.06 (0.04 to 0.09) |
| Morocco | 34.9 (15-79.05) | 0.38 (0.17-0.87) | 86.18 (37.1-195.52) | 0.39 (0.17-0.88) | 0.02 (-0.02 to 0.06) |
| Mozambique | 11.25 (4.65-26.18) | 0.35 (0.14-0.81) | 24.61 (9.85-58.09) | 0.39 (0.15-0.91) | 0.47 (0.45 to 0.5) |
| Myanmar | 46.2 (19.33-108.94) | 0.31 (0.13-0.72) | 114.47 (47.29-259.37) | 0.33 (0.14-0.77) | 0.23 (0.19 to 0.28) |
| Namibia | 1.44 (0.61-3.35) | 0.31 (0.13-0.72) | 3.62 (1.53-8.55) | 0.35 (0.14-0.82) | 0.37 (0.35 to 0.39) |
| Nauru | 0.01 (0-0.02) | 0.38 (0.14-0.93) | 0.01 (0-0.01) | 0.35 (0.14-0.87) | -0.3 (-0.34 to -0.26) |
| Nepal | 12.27 (5.31-28) | 0.23 (0.1-0.53) | 41.6 (17.35-98.19) | 0.27 (0.11-0.64) | 0.63 (0.6 to 0.65) |
| Netherlands | 67.57 (31.05-145.76) | 0.33 (0.15-0.72) | 124.53 (56.71-264.12) | 0.32 (0.15-0.69) | -0.13 (-0.18 to -0.09) |
| New Zealand | 12.1 (5.42-26.74) | 0.33 (0.15-0.72) | 28.81 (13.21-62.14) | 0.33 (0.15-0.7) | -0.08 (-0.12 to -0.04) |
| Nicaragua | 3.87 (1.61-8.73) | 0.33 (0.14-0.74) | 11.5 (4.75-26.25) | 0.35 (0.14-0.78) | 0.16 (0.12 to 0.21) |
| Niger | 4.65 (1.84-11.47) | 0.32 (0.13-0.79) | 14.91 (5.98-35.97) | 0.34 (0.14-0.82) | 0.28 (0.25 to 0.3) |
| Nigeria | 82.93 (34.11-203.68) | 0.29 (0.12-0.73) | 180.76 (69.76-425.65) | 0.33 (0.12-0.78) | 0.58 (0.47 to 0.69) |
| Niue | 0.01 (0-0.02) | 0.4 (0.16-0.95) | 0.01 (0-0.02) | 0.36 (0.14-0.81) | -0.48 (-0.53 to -0.43) |
| North Macedonia | 4.42 (2-10.08) | 0.33 (0.15-0.76) | 8.26 (3.74-17.83) | 0.36 (0.16-0.77) | 0.23 (0.21 to 0.25) |
| Northern Mariana Islands | 0.03 (0.01-0.07) | 0.35 (0.14-0.8) | 0.1 (0.04-0.24) | 0.34 (0.14-0.78) | -0.04 (-0.07 to -0.02) |
| Norway | 24.91 (11.2-54.73) | 0.33 (0.15-0.72) | 34.51 (15.65-74.25) | 0.3 (0.14-0.64) | -0.22 (-0.25 to -0.2) |
| Oman | 1.46 (0.61-3.41) | 0.44 (0.18-0.99) | 3.31 (1.39-7.77) | 0.42 (0.18-0.97) | -0.09 (-0.13 to -0.04) |
| Pakistan | 110.87 (44.54-265.7) | 0.26 (0.1-0.62) | 183.78 (73.51-449.57) | 0.28 (0.11-0.68) | 0.27 (0.26 to 0.29) |
| Palau | 0.02 (0.01-0.05) | 0.32 (0.13-0.74) | 0.04 (0.02-0.09) | 0.3 (0.13-0.68) | -0.25 (-0.3 to -0.2) |
| Palestine | 2.68 (1.14-6.11) | 0.41 (0.17-0.92) | 5.82 (2.54-13.51) | 0.39 (0.17-0.9) | -0.18 (-0.23 to -0.13) |
| Panama | 4.3 (1.78-9.64) | 0.33 (0.13-0.73) | 14.3 (6.1-30.75) | 0.33 (0.14-0.72) | 0.07 (0.05 to 0.1) |
| Papua New Guinea | 3.09 (1.26-7.41) | 0.32 (0.13-0.73) | 7.82 (3.28-18.48) | 0.31 (0.13-0.73) | -0.12 (-0.15 to -0.1) |
| Paraguay | 6.73 (2.93-15.3) | 0.35 (0.15-0.81) | 18.55 (8.17-40.47) | 0.37 (0.16-0.8) | 0.13 (0.1 to 0.16) |
| Peru | 30.29 (12.59-71.27) | 0.3 (0.13-0.71) | 99.73 (43.07-220.07) | 0.31 (0.13-0.68) | 0.07 (0.04 to 0.09) |
| Philippines | 73.8 (28.56-176.82) | 0.38 (0.15-0.91) | 180.94 (73.06-419.38) | 0.33 (0.13-0.78) | -0.6 (-0.77 to -0.44) |
| Poland | 141.84 (62.33-319.26) | 0.38 (0.17-0.84) | 275.41 (125.94-609.35) | 0.36 (0.16-0.79) | -0.23 (-0.27 to -0.2) |
| Portugal | 34.92 (15.98-77.89) | 0.29 (0.13-0.66) | 91.12 (41.41-195.81) | 0.3 (0.14-0.64) | 0.1 (0.08 to 0.11) |
| Puerto Rico | 10.4 (4.37-23.65) | 0.31 (0.13-0.71) | 26.85 (11.59-58.85) | 0.31 (0.13-0.68) | -0.02 (-0.04 to -0.01) |
| Qatar | 0.17 (0.07-0.41) | 0.39 (0.16-0.89) | 1.2 (0.49-2.86) | 0.41 (0.17-0.95) | 0.2 (0.16 to 0.25) |
| South Korea | 62.63 (27.71-138.97) | 0.37 (0.16-0.81) | 286.94 (131.41-603.05) | 0.35 (0.16-0.72) | -0.2 (-0.3 to -0.11) |
| Moldova | 9.9 (4.54-21.89) | 0.33 (0.15-0.74) | 19.24 (8.95-41.58) | 0.33 (0.15-0.72) | -0.02 (-0.06 to 0.03) |
| Romania | 69.34 (31.23-157.15) | 0.33 (0.15-0.76) | 139.09 (64.27-300.86) | 0.34 (0.16-0.73) | 0.04 (0.03 to 0.05) |
| Russia | 422.39 (195.45-937.17) | 0.31 (0.14-0.68) | 755.1 (351.53-1619.56) | 0.32 (0.15-0.69) | 0.18 (0.16 to 0.19) |
| Rwanda | 4.98 (2.15-11.53) | 0.31 (0.13-0.72) | 12.97 (5.38-30.23) | 0.37 (0.15-0.86) | 0.85 (0.75 to 0.96) |
| Saint Kitts | 0.1 (0.04-0.23) | 0.3 (0.12-0.7) | 0.14 (0.06-0.31) | 0.3 (0.12-0.68) | 0.03 (0 to 0.05) |
| Saint Lucia | 0.22 (0.09-0.51) | 0.3 (0.12-0.7) | 0.61 (0.26-1.4) | 0.31 (0.13-0.71) | 0.05 (0.02 to 0.07) |
| Saint Vincent | 0.19 (0.08-0.46) | 0.31 (0.13-0.73) | 0.35 (0.15-0.8) | 0.3 (0.12-0.68) | -0.12 (-0.15 to -0.1) |
| Samoa | 0.25 (0.1-0.6) | 0.4 (0.16-0.96) | 0.44 (0.17-1.03) | 0.38 (0.15-0.89) | -0.25 (-0.29 to -0.21) |
| San Marino | 0.1 (0.04-0.22) | 0.31 (0.14-0.69) | 0.25 (0.11-0.53) | 0.3 (0.14-0.64) | -0.06 (-0.09 to -0.03) |
| Sao Tome and Principe | 0.13 (0.05-0.31) | 0.29 (0.12-0.69) | 0.23 (0.09-0.52) | 0.31 (0.12-0.72) | 0.21 (0.17 to 0.25) |
| Saudi Arabia | 14.61 (6.19-34.16) | 0.4 (0.17-0.91) | 32.53 (13.94-72.98) | 0.39 (0.17-0.86) | -0.1 (-0.12 to -0.08) |
| Senegal | 7.11 (2.79-17.34) | 0.34 (0.13-0.81) | 18.97 (7.29-44.17) | 0.36 (0.14-0.85) | 0.33 (0.27 to 0.4) |
| Serbia | 29 (13.02-64.27) | 0.32 (0.15-0.73) | 46.57 (21.58-100.77) | 0.33 (0.15-0.7) | 0.01 (-0.02 to 0.03) |
| Seychelles | 0.19 (0.08-0.43) | 0.34 (0.14-0.79) | 0.3 (0.12-0.66) | 0.33 (0.14-0.74) | -0.1 (-0.12 to -0.08) |
| Sierra Leone | 4.21 (1.7-10.03) | 0.3 (0.12-0.73) | 7.69 (3.05-18.34) | 0.32 (0.13-0.77) | 0.27 (0.25 to 0.3) |
| Singapore | 4.76 (2.03-10.63) | 0.32 (0.14-0.71) | 22.4 (10.24-46.93) | 0.32 (0.15-0.67) | 0.1 (0.03 to 0.17) |
| Slovakia | 18.37 (8.22-41.1) | 0.35 (0.16-0.78) | 31.07 (14.34-66.5) | 0.35 (0.16-0.74) | 0.02 (0 to 0.04) |
| Slovenia | 7.98 (3.6-17.17) | 0.36 (0.16-0.77) | 19.09 (8.7-40.93) | 0.36 (0.17-0.77) | 0.09 (0.05 to 0.12) |
| Solomon Islands | 0.19 (0.08-0.47) | 0.29 (0.12-0.69) | 0.48 (0.2-1.15) | 0.31 (0.13-0.73) | 0.18 (0.14 to 0.23) |
| Somalia | 3.73 (1.63-8.38) | 0.3 (0.13-0.67) | 10.15 (4.35-24) | 0.33 (0.14-0.78) | 0.56 (0.48 to 0.63) |
| South Africa | 49.94 (21.53-115.13) | 0.31 (0.13-0.7) | 106.64 (46.24-244.75) | 0.32 (0.14-0.73) | 0.09 (0.05 to 0.14) |
| South Sudan | 4.84 (2.07-11.14) | 0.31 (0.13-0.71) | 7.44 (3.12-17.15) | 0.31 (0.13-0.74) | 0.05 (0.03 to 0.08) |
| Spain | 189.23 (85.3-412.5) | 0.36 (0.16-0.78) | 382.6 (174.06-804.56) | 0.3 (0.14-0.64) | -0.63 (-0.77 to -0.48) |
| Sri Lanka | 25.74 (10.4-59.43) | 0.35 (0.14-0.81) | 70.27 (28.87-160.98) | 0.34 (0.14-0.77) | 0.08 (0.03 to 0.13) |
| Sudan | 23.15 (10.13-53.3) | 0.38 (0.17-0.88) | 48.14 (21.13-107.83) | 0.37 (0.16-0.84) | -0.16 (-0.19 to -0.13) |
| Suriname | 0.69 (0.28-1.59) | 0.3 (0.12-0.69) | 1.6 (0.66-3.5) | 0.31 (0.13-0.68) | 0.02 (-0.02 to 0.07) |
| Sweden | 54.26 (24.44-119.7) | 0.32 (0.14-0.7) | 73.66 (33.08-160.45) | 0.28 (0.13-0.61) | -0.41 (-0.46 to -0.36) |
| Switzerland | 34.75 (15.69-76.3) | 0.3 (0.14-0.67) | 67.11 (30.61-143.03) | 0.31 (0.14-0.64) | -0.02 (-0.07 to 0.02) |
| Syria | 14.48 (6.32-33.1) | 0.38 (0.17-0.87) | 30.69 (13.25-70.35) | 0.39 (0.17-0.91) | 0.1 (0.08 to 0.13) |
| Taiwan | 31 (12.66-71.77) | 0.29 (0.12-0.67) | 132.21 (58.19-291.58) | 0.32 (0.14-0.72) | 0.5 (0.42 to 0.58) |
| Tajikistan | 8.77 (4-18.93) | 0.33 (0.15-0.71) | 8.44 (3.69-19.17) | 0.36 (0.16-0.8) | 0.3 (0.27 to 0.33) |
| Thailand | 88.18 (35.47-205.46) | 0.35 (0.14-0.83) | 330.66 (142.03-717.9) | 0.34 (0.15-0.73) | -0.2 (-0.26 to -0.14) |
| Timor-Leste | 0.51 (0.21-1.24) | 0.31 (0.13-0.73) | 1.89 (0.77-4.52) | 0.33 (0.13-0.79) | 0.26 (0.19 to 0.33) |
| Togo | 2.42 (0.95-5.79) | 0.33 (0.13-0.81) | 6.88 (2.72-16.26) | 0.34 (0.14-0.8) | 0.09 (0.07 to 0.1) |
| Tokelau | 0 (0-0.01) | 0.39 (0.16-0.95) | 0 (0-0.01) | 0.35 (0.14-0.81) | -0.4 (-0.42 to -0.39) |
| Tonga | 0.15 (0.06-0.34) | 0.36 (0.15-0.84) | 0.26 (0.11-0.59) | 0.35 (0.15-0.79) | -0.07 (-0.1 to -0.04) |
| Tobago | 1.9 (0.79-4.43) | 0.29 (0.12-0.68) | 4.96 (2.11-11.23) | 0.29 (0.12-0.66) | 0.03 (0.01 to 0.05) |
| Tunisia | 13.45 (5.84-30.72) | 0.41 (0.18-0.91) | 42.02 (18.28-94.96) | 0.4 (0.17-0.89) | -0.08 (-0.09 to -0.08) |
| Turkey | 111.19 (48.2-246.84) | 0.4 (0.18-0.89) | 313.66 (142.25-713.15) | 0.4 (0.18-0.89) | -0.05 (-0.1 to 0.01) |
| Turkmenistan | 4.53 (2.03-9.99) | 0.35 (0.16-0.78) | 9.44 (4.29-20.65) | 0.35 (0.16-0.76) | -0.07 (-0.09 to -0.05) |
| Tuvalu | 0.02 (0.01-0.04) | 0.4 (0.16-0.93) | 0.03 (0.01-0.06) | 0.36 (0.14-0.86) | -0.34 (-0.38 to -0.3) |
| Uganda | 12.25 (5.29-28.39) | 0.31 (0.13-0.71) | 30.34 (12.92-68.14) | 0.35 (0.15-0.79) | 0.43 (0.4 to 0.45) |
| Ukraine | 177.53 (82.53-396.74) | 0.3 (0.14-0.67) | 260.91 (117.5-583.33) | 0.34 (0.15-0.76) | 0.4 (0.35 to 0.45) |
| United Arab Emirates | 0.58 (0.23-1.39) | 0.39 (0.16-0.92) | 4.08 (1.62-9.98) | 0.37 (0.16-0.83) | -0.24 (-0.3 to -0.18) |
| UK | 279.04 (122.02-633.51) | 0.3 (0.13-0.67) | 438.16 (191.86-960.16) | 0.29 (0.13-0.65) | -0.05 (-0.06 to -0.03) |
| Tanzania | 21.68 (8.97-51.46) | 0.34 (0.14-0.8) | 58.41 (23.86-137.97) | 0.35 (0.14-0.81) | 0.12 (0.09 to 0.15) |
| USA | 1135.56 (545.21-2411.34) | 0.33 (0.16-0.7) | 2026.88 (979.98-4180.76) | 0.32 (0.16-0.66) | -0.22 (-0.27 to -0.16) |
| Virgin Islands | 0.19 (0.08-0.45) | 0.32 (0.13-0.75) | 0.49 (0.21-1.12) | 0.31 (0.13-0.68) | -0.1 (-0.13 to -0.08) |
| Uruguay | 11.53 (5.22-25.58) | 0.31 (0.14-0.68) | 20.81 (9.58-45.04) | 0.31 (0.14-0.67) | 0.04 (0.02 to 0.06) |
| Uzbekistan | 30.65 (14.06-66.42) | 0.33 (0.15-0.71) | 27.01 (12.23-61.29) | 0.35 (0.16-0.8) | 0.26 (0.22 to 0.29) |
| Vanuatu | 0.13 (0.05-0.32) | 0.33 (0.14-0.81) | 0.37 (0.15-0.93) | 0.34 (0.13-0.81) | -0.05 (-0.12 to 0.02) |
| Venezuela | 25.39 (10.55-58.62) | 0.34 (0.14-0.77) | 87.92 (36.01-194.56) | 0.33 (0.14-0.74) | -0.03 (-0.07 to 0.01) |
| Vietnam | 122.55 (49.2-284.16) | 0.38 (0.15-0.86) | 297.06 (117.25-676.85) | 0.41 (0.16-0.94) | 0.32 (0.29 to 0.35) |
| Yemen | 10.52 (4.63-24.31) | 0.39 (0.17-0.87) | 31.78 (13.66-72) | 0.38 (0.17-0.89) | -0.01 (-0.04 to 0.02) |
| Zambia | 5.14 (2.13-11.74) | 0.32 (0.13-0.74) | 13.25 (5.55-30.57) | 0.34 (0.14-0.78) | 0.36 (0.31 to 0.42) |
| Zimbabwe | 8.16 (3.44-18.64) | 0.34 (0.14-0.78) | 14.07 (5.93-32.77) | 0.35 (0.15-0.8) | 0.07 (0.02 to 0.12) |

DALYs, disability-adjusted life years; ASR, age-standardized rate; EAPC, estimated annual percent change;

**Supplement Table 5.** ASDR and ASR of DALYs for Alzheimer's disease and other dementias associated risk factors in 2019.

| **Risk factors** | **Region** | **ASDR (per 100,000) (95% UI)** | **ASR of DALYs (per 100,000) (95% UI)** |
| --- | --- | --- | --- |
| **All risk factors** | Global | 7.07 (1.55-20.89) | 111.34 (43.2-273.86) |
|  | High SDI | 7.56 (1.69-21.41) | 120.88 (49.78-282.72) |
|  | High-middle SDI | 7.49 (1.63-22.15) | 122.65 (48.42-302.74) |
|  | Middle SDI | 6.72 (1.43-20.12) | 106.99 (39.56-269.38) |
|  | Low-middle SDI | 5.81 (1.23-17.74) | 86.38 (30.44-223.36) |
|  | Low SDI | 4.71 (0.96-14.78) | 68.65 (22.57-183.31) |
|  |  |  |  |
|  | Female | 6.34 (1.23-18.5) | 98.03 (33.91-238.23) |
|  | Male | 8.14 (1.91-23.5) | 128.8 (51.81-303.9) |
|  |  |  |  |
| **High body-mass index** | Global | 2.81 (0.46-8.42) | 42.22 (11.24-107.37) |
|  | High SDI | 3.14 (0.53-9.32) | 48.51 (13.77-119.05) |
|  | High-middle SDI | 3.32 (0.56-9.98) | 51.13 (14.09-128.38) |
|  | Middle SDI | 2.38 (0.39-7.3) | 36.54 (8.96-98.38) |
|  | Low-middle SDI | 1.79 (0.29-5.61) | 25.82 (5.94-70.31) |
|  | Low SDI | 1.42 (0.21-4.65) | 20.65 (4.29-59.46) |
|  |  |  |  |
|  | Female | 3.13 (0.43-9.76) | 47.78 (9.54-131.09) |
|  | Male | 2.26 (0.23-7.35) | 34.05 (5.72-93.66) |
|  |  |  |  |
| **High fasting plasma glucose** | Global | 2.24 (0.26-8.24) | 32.84 (5.53-105.88) |
|  | High SDI | 2.29 (0.26-8.38) | 34.05 (6.12-108.13) |
|  | High-middle SDI | 2.11 (0.23-7.88) | 31.64 (5.4-101.61) |
|  | Middle SDI | 2.31 (0.26-8.52) | 33.53 (5.51-108.94) |
|  | Low-middle SDI | 2.21 (0.25-8.18) | 31.1 (5.17-102.47) |
|  | Low SDI | 2.03 (0.22-7.75) | 28.12 (4.54-96.25) |
|  |  |  |  |
|  | Female | 2.27 (0.2-8.79) | 33.6 (4.79-111.46) |
|  | Male | 2.2 (0.2-8) | 31.79 (4.48-99.84) |
|  |  |  |  |
| **Smoking** | Global | 2.91 (0.64-8.06) | 51.3 (20.5-116.23) |
|  | High SDI | 3.25 (0.75-8.88) | 57.94 (23.72-127.69) |
|  | High-middle SDI | 3.02 (0.67-8.4) | 56.75 (23.05-125.49) |
|  | Middle SDI | 2.79 (0.64-7.83) | 49.67 (19.33-114.43) |
|  | Low-middle SDI | 2.4 (0.52-6.88) | 38.72 (14.67-92.3) |
|  | Low SDI | 1.64 (0.35-4.69) | 25.67 (8.96-62.13) |
|  |  |  |  |
|  | Female | 1.64 (0.37-4.55) | 27.9 (11.05-62.62) |
|  | Male | 4.89 (1.11-13.55) | 83.12 (32.82-189.42) |

ASDR, age-standardized deaths rate; ASR, age-standardized rate; DALYs, disability-adjusted life years.
